# Combination of different intervention strategies for malaria mosquito vector control in Uganda: A review of secondary data of two districts with moderate to high disease transmission

**DOI:** 10.1371/journal.pone.0352455

**Published:** 2026-07-06

**Authors:** Erick Jacob Okek, Winnie Nambatya, Loyce Nakalembe, Silvia Awor, Benson Musinguzi, Amos Drasiku, Sande James Obondo, Martin Lukindu, Julius Lutwama, Jonathan Kayondo, Moses Ocan

**Affiliations:** 1 Department of Immunology and Molecular Biology, School of Biomedical Sciences, College of Health Sciences, Makerere University, Kampala, Uganda; 2 Department of Entomology and Parasitology, Uganda Virus Research Institute, Entebbe, Uganda; 3 Department of Arbovirology and Zoonotic, Uganda Virus Research Institute, Entebbe, Uganda; 4 Department of Pharmacology and Therapeutics, School of Biomedical Sciences, College of Health Sciences, Makerere University, Kampala, Uganda; 5 Department of Pharmacology, Soroti University, Soroti, Uganda; 6 Department of Reproductive Health, Faculty of Medicine, Gulu University, Gulu, Uganda; 7 Department of Medical Laboratory Sciences, Faculty of Health Sciences, Muni University, Arua, Uganda; 8 Department of Epidemiology and Biostatistics, School of Medicine, College of Health Sciences, Makerere University, Kampala, Uganda; Virginia Commonwealth University, UNITED STATES OF AMERICA

## Abstract

**Background:**

Indoor residual spraying (IRS) and insecticide treated bed nets (ITNs) used either singly or in combination are the main mass mosquito vector control measures for malaria control. Despite their widespread use, malaria transmission rates remain high, the burden is unacceptably huge, and yet the disease is completely preventable. This study measured the impact of compounds used in different types and campaigns of IRS and ITNs, different permutations of IRS + ITNs on malaria test positivity rates in high disease transmission settings of Yumbe district and Gulu district.

**Method:**

The Ministry of Health’s District Health Information System 2 (DHIS 2) records on distribution of ITNs, IRS schedules and malaria cases by gender, age and geographical location (Gulu and Yumbe districts) collected over a five-year period (DHIS 2 records accessed on 11^th^ February 2025) were used in the final analysis. Data collection was done using a checklist developed in an Excel spreadsheet. Data on the following were extracted; socio-demographic characteristics, number of monthly malaria tests, monthly numbers of positive malaria tests, the type of Insecticide Treated Nets (ITNs) distributed, the type of Indoor Residual Spray (IRS) applied and the time points that the interventions were deployed. The data was exported into STATA ver 17.0,cleaned and additional variables generated prior to the interrupted time series and Difference in Difference analysis. The monthly malaria test positivity rate (TPR) was calculated while adjusting for variability in rainfall, temperature and relative humidity. A regression analysis and graphical plots using the Generalized Estimation Equation (GEE) population average models were performed.

**Results:**

After controlling for monthly variation in rainfall, temperature, and relative humidity, the deployment of malaria vector control interventions in Yumbe district led to a much faster reduction in TPR of 0.006 units/0.56% per month; p < 0.00 (about 5 times faster than Gulu district). The 2020 distribution of Yorkool ITNs in Yumbe did not significantly change the long-term trend in TPR of the district relative to Gulu that maintained distribution of PermaNets ITN (trend change difference = 0.0111, beta = −0.0054, 95% CI:0.1145, 0.1038, p = 0.923). Fludora Fusion demonstrated a profound impact. The difference-in-differences interaction term was highly statistically significant, showing an absolute 21.2% drop in the Malaria Test Positivity Rate in Yumbe District relative to Gulu district (beta = −0.2123, 95% CI: −0.2801, −0.1444, p < 0.001). Following a co-deployment of Actellic 300 CS IRS+ Royal Guard ITNs in Yumbe and distribution of PermaNet ITNs in Gulu, the difference-in-differences interaction term was highly statistically significant, demonstrating an absolute 25.1% drop in the Malaria Test Positivity Rate in Yumbe District compared to Gulu district (beta = −0.2508, 95%CI:-0.3279, −0.1737,p < 0.001).

**Conclusion:**

In areas of high malaria transmission, deployment of either ITNs alone, IRS alone, or in combination can be an effective tool for malaria case reduction. However, a more sustained and significant reduction is achieved through the simultaneous deployment of IRS and ITNs. Crucially, the efficacy of these combined interventions is highly shaped by the specific classes of insecticides and active compounds utilized within the deployed ITN types and IRS campaigns.

## Introduction

WHO projects that the key 2030 malaria indicator morbidity and mortality milestones will be missed by 89% and 88% respectively if changes in Malaria control are not implemented immediately [1]. To avert this looming disaster, they recommended series of actions; including a universal coverage of either Indoor residual spray (IRS) or Insecticide Treated Nets (ITNs) as core mass malaria mosquito vector control interventions [2]. Other methods that target reduction in parasite transmission and elimination are Intermittent Preventive Therapy (IPT), mass drug administration and recently vaccines have been added to the arsenal for malaria control [3].

Protective efficacy of ITNs has been reported to be greater in high transmission settings when utilized for a longer period of time [4]. A study [5] reported that malaria incidence in 64 sites in Uganda that received ITNs decreased from 827 cases per 1000 person-years in the pre-distribution phase to 538 cases per 1000 person years in the post distribution period [5] demonstrating the effectiveness of this intervention. In many malaria-endemic countries, ITNs are mostly distributed by authorities as part of a wider malaria control campaign [6]. Routinely, there are targeted distribution to pregnant mothers during antenatal visits and to infants during immunizations. With over 80% community coverage and at least one net per two persons, this intervention alone can sufficiently protect against new Malaria infections [7]. In 2010, the Ugandan Ministry of Health distributed ITNs to pregnant women only. In 2013–2014, 2017–2018 and 2020–2021, there was mass distribution of ITNs to all population at risk in all regions of the country [8] achieving a significantly higher coverage of up to 94.1% after the 2020/2021 distribution cycle.

IRS was re-introduced in Uganda in 2006 following decades of suspension. Ten districts in Northern Uganda with high perennial malaria transmission at the time (including Gulu and Yumbe) were selected for the first phase of implementation [9]. Between 2006–2014, three rounds of IRS using a combination of different compounds such as lambda cyhalothrin, alpha-cypermethrin, bendiocarb and pirimiphos-methyl were applied across the ten districts. Each round was followed by an immediate sharp reduction in cases then a dramatic resurgence at few months after the application. Mass IRS was eventually stopped in some districts due to logistical factors and non-satisfactory performance [10]. Among regions that sustained implementation of IRS was Eastern Uganda; specifically, Tororo district. Kamya MR et al 2024 [11] documented trends in selected malaria metrics at different time points of IRS implementation in Tororo district from October 2011 to September 2023. They reported a remarkable reduction in cases and parasite prevalence between 2015–2020 when Actellic 300 CS and Bendiocarb were the compounds in use for the IRS. This was followed by a dramatic resurgence between April 2020 to March 2023 when the compounds were changed to Fludora Fusion and Sumishield. When it was reverted to Actellic 300 CS, cases immediately reduced [11]. The trend clearly indicates that the effectiveness of IRS is dependent on many external factors, including the compound of choice and associated mosquito vector resistance pattern.

Many countries have chosen to simultaneously deploy a combination of ITNs and IRS in order to achieve the 2030 WHO malaria elimination target [12]. By January 2024, thirty-one out of 57 sub-Saharan African countries were implementing a co-deployment of IRS and ITNs for malaria control despite contrasting reports on the effectiveness of such a combination [1]. *Echodu.R. et al* reported that implementation of IRS combined with ITNs was associated with 97.6% decrease in malaria incidence and 96% decrease in parasite prevalence, both measured after several rounds of IRS. However, when chemoprophylaxis is added, there was an even more drastic decrease in malaria incidence of up to 99.5% [13]. To the contrary, a randomized controlled trial in Benin found no added protective effects in the deployment of IRS + ITNs over bed nets alone [14]. A four-year prospective study that evaluated the National Malaria control program in Eritrea found no added advantage of using IRS and ITNs as opposed to using either method alone [15]. An observational study conducted in Ethiopia reported that while both intervention arms (PBO nets alone versus Standard ITNs + IRS) were associated with reduced malaria cases, there was a difference in sustained effects; with ITNs + IRS lasting longer [16].

Despite conflicting reports on the impact of multiple interventions on Malaria control, Uganda is already administering R21 MM vaccines to children in areas with heavy deployment of both indoor residual spray and long-lasting insecticide treated nets. Simultaneous deployment of many interventions has a huge logistical and financial cost associated with it. The additional protection it offers should be commensurate to the implementation cost. Studies conducted in Uganda were clinical trials evaluating the impacts of single interventions. No study reviewed secondary data to compare the effectiveness of different combinations of malaria vector control measures in moderate to high transmission settings such as Gulu and Yumbe. This study measured the impact of compounds used in different formulations of IRS and ITNs, different permutations of IRS + ITNs on malaria test positivity rates in high disease transmission settings of Yumbe district and Gulu district.

## Materials and methods

### Study settings, population and deployment of malaria mosquito vector control interventions

This study reviewed Malaria related demographic and clinical data from District Health Information Systems (DHIS2) of Gulu and Yumbe districts.

### Yumbe district (IRS + ITNs)

Yumbe district is located in the West Nile Region of Uganda (Table 1). It directly shares border with the Republic of South Sudan on the Northern part and the Democratic Republic of Congo to the west (Table 1). It has 26 sub-counties, 7 town councils, 197 parishes, approximately 1,159 villages and harboring approximately 945,000 people. It is located approximately 600kms away from Kampala (The administrative and economic capital of Uganda). Malaria transmission is perennial in this area. Transmission peaks during rainy season (between April to October) and drops during dry season. According to the data from the Uganda metrological center, Yumbe received significantly higher rainfall intensity from the Month of May to December. The area has one of the highest entomological inoculation rates globally. Because of the complex population mixture (highest concentration of refugee camps), high transmission and strategic location, Government and partners have intensively invested in the deployment of different combination of malaria control interventions in the area; notably, Indoor Residual Spray (IRS) and mass distribution of Insecticide Treated Bed nets (ITNs).

**Table 1:** IRS and ITN distribution campaigns in Yumbe and Gulu.

	2020/21	2022/2023	2023/24	2024/25
Yumbe district	Yorkool ITNs	Fludora Fusion (IRS)	Actellic 300 CS(IRS) and Royal Guard ITNs	Actellic 300 CS, and Royal Guard ITNs
Gulu district	PermaNet ITNs(fresh distribution)	Inter-campaign period	PermaNet ITNs	Inter-campaign period

From January 2020 to December 2024, three rounds of IRS were sprayed and two rounds of ITNs were distributed in Yumbe district. In December 2020, there was mass distribution of ITNs in all sub-counties, including refugee camps. All the 18 sub-counties received standard ITNs called Yorkool. The Yorkool net is a G5 LN (WHO prequalified vector control product) model chemically composed of Alpha-cypermethrin and chlorfenapyr. In December 2022, there was a district wide application of indoor residual spray (IRS) using Fludora Fusion. Fludora is a mixture of Clothianidin (50% w/w or 500 g/kg) and deltamethrin (6.25% w/w or 62.5 g/kg). In December 2023, there was another mass distribution of ITNs; but the types were changed to Royal Guard. Royal Guard is chemically composed of alpha-cypermethrin and pyriproxyfen. In December 2023, another cycle of mass IRS was repeated but using a different compound; Actellic 300 CS (Table 1). Actellic 300 CS is an organophosphate majorly composed of pirimiphos-methyl. In December 2024, Actellic 300 CS was again applied in the same area. However, this study did not measure the impact of the last round of IRS application. These interventions were deployed on top of the ITNs given to babies and mothers during immunization and antenatal visits. In addition, gestational mothers were equally given ITNs alongside Intermittent Preventive Therapy in pregnancy (IPTp).

### Gulu District (ITNS only)

Gulu district is located in the Mid Northern part of Uganda. The city is the main administrative and economic center of the region. Because of its strategic location between Kampala (about 350 Kms) and Juba (about 700 kms), it has become an economic hub for Uganda and South Sudan. Acholi is the predominant indigenous ethnic group. Farming and trade are the main economic activity in the rural and city settings respectively. From 1990 to about 2010, the Acholi region went through turmoil from the armed conflicts by the Lord Resistance Army (LRA). Thousands were displaced and put in internally displaced People’s (IDPs) camps. The war reversed many gains in the health sector, especially malaria reduction and elimination achievements. Because of many swamps, low and flat lands and the nature of vegetations, the region has many fertile grounds for mosquito breeding. Just like Yumbe, the area has high perennial malaria transmission. It also has a similar rainfall pattern; with wet season stretching from April to October and dry spell after. According to the data from the Uganda metrological center, Gulu receive significantly higher rainfall intensity from the Month of May to December. Malaria burden in this region is one of the highest in Uganda and globally

In 2006, Gulu was among ten districts selected for the pilot roll out of IRS in Northern Uganda. The program went on consistently till 2014 when it was temporarily suspended due to non-satisfactory impact, logistical factors among others. From 2015 till date, Government of the republic of Uganda and partners embarked on mass distribution of ITNs as the sole intervention measure. After every three years, there is a large-scale distribution of ITNs. This is on top of the ones given to mothers during antenatal visits and babies during immunization schedules. From 2020 till when this data was abstracted, two rounds of ITNs were distributed in the communities. In December 2020, fifteen(15) sub-counties received PermaNet ITNs. PermaNet is a blend of Deltamethrin (approx. 4.0 g/kg) and Piperonyl Butoxide (PBO) (approx. 25 g/kg); (Table 1). In December 2023, another round of mass ITNs distribution was implemented using PermaNet brands (Table 1).

### Data collection and extraction

We briefly describe how DHIS 2 data was generated. When patients arrive at a Health facility, a clinical examination and clerkship is done. Following clinical assessment, patients are referred to the Laboratory for malaria blood tests, test conducted and results together with accompanying meta data entered into the Laboratory register. Routinely, health facilities organize outreaches to communities. Malaria tests using RDTs are usually conducted. Other community programs include integrated immunization and vaccination, mass malaria testing and treatment. Apart from outpatient departments, malaria tests are also done in the maternity wards, inpatients departments, antenatal clinics, special clinics among others. Data generated from all the different points are summarized into the Health Information Management system (HIMS) and submitted to respective districts. Districts consolidate all information and submit to the Ministry of Health through District Health Information System 2 (DHIS 2). This is the data that we reviewed, analyzed and reported in this article.

### Ethical approval/consideration

The study was approved by the Makerere University School of Biomedical Sciences Research and Ethics Committee (SBS-REC) with approval number SBS-2023-362. Study was also approved by Uganda National Council of Science and Technology (UNCST) with approval number HS5306ES. To access data, permission was obtained from the Director General of Health Services, Ministry of Health. Access to data was granted on 11^th^ February 2025 and data retrieval followed immediately (approval letter attached). There was no need to obtain consent from individual patients because the data was anonymized.

### Statistical analysis

We summarized the demographic and clinical information of participants in a table using Microsoft word. Using a trend plot in STATA ver 17, we visually displayed a five (5) years trend in malaria test positivity rates for Yumbe and Gulu district respectively. A regression analysis was performed to quantitatively determine the relationship between test positivity rate and deployment of mass malaria control intervention. The Harris-Tzavalis (HT) test informed the decision to use the stationary rates to perform a regression analysis as opposed to converting it to first difference. We utilized a Binomial family with a Logit link function and an Exchangeable correlation structure to account for the longitudinal nature of the data.Time was stratified into pre-intervention, intervention and post-interventions periods for the December 2020 ITN distribution phase, December 2022 IRS application in Yumbe, and December 2023 co-deployment of IRS+ITNS in Yumbe, ITNS distribution in Gulu district. Using Generalized Estimation Equation(GEE) population averaged model, we statistically determined the impact of different combinations of control interventions on TPR at different points after adjusting for rainfall, temperature and relative humidity. Because of low cluster, we used standard OLS regression with an interaction term to get the estimate. A parallel trends assumption was performed and parallel trend graphs plotted to address the same challenge of OLS underestimating the standard error in DiD that could potentially make the results look more significant than they actually are.

## Results

### Summary of demographic and clinical information

Yumbe had an average Test Positivity Rate (TPR) of 57.4%) while that of Gulu was 42.2% (Table 1). Household ITNs coverage was higher in Gulu (86%) compared to Yumbe (74.15%). Household IRS coverage in Yumbe was 74.15%. ITNs constituted 72.27% of total bed nets in Gulu (Table 2). For the period, a total of 2,851,218 malaria tests were done in Yumbe while 2,017,655 malaria tests were done in Gulu. For Yumbe, number of cases were highest in children aged 29 days to 4 years 512,970 (31.3%) while for Gulu, cases were highest in adults (Table 1). Malaria was more prevalent among gestational mothers in Gulu (3.07%) compared to Yumbe ((1.76%) (Table 2).

**Table 2:** Demographic and clinical characteristics of patients from Yumbe district and Gulu district.

Characteristics/variable	Yumbe district	Gulu district
Malaria control interventions	Indoor Residual Spraying (IRS), Insecticide Treated Nets (ITNs)	Insecticide Treated Nets (ITNs)
Intervention coverage in the communities	74.15% of households were sprayed with IRS,98.42% had at least one bed net	86% had at least one bed net, 72.7% being PBO nets
Total number of Malaria Tests done (Microscopy + RDTs)	2,851,218	2,017,655
Microscopy done	297,716	537,222
RDTs done	2,553,502	1,480,433
Malaria positivity by microscopy	158,603 (53.27%)	148,082(27.56%)
Malaria Positivity by RDTs	1,479,198(57.9%)	704,775(47.6%)
Malaria diagnosis by age category		
0 to 28 days	432 (0.1%)	780 (0.9%)
10 years to 19 years	423,133 (25.8%)	261,503 (30.6%)
20 + years	423,905 (25.8%)	276,425 (32.4%)
29 days to 4 years	512,970 (31.3%)	192,648 (22.6%)
5 years to 9 years	329,064 (20.1%)	151,232 (17.7%)
Malaria in pregnancy		
10 to 19 years	9620 (0.59%)	8373 (0.98%)
+ 20 years	19287 (1.17%)	17838 (2.09%)
Mean Parasite prevalence		
Below 15 years	428.35 parasites/ul	359.69 parasites/ul
Above 15 years	128.43 parasites/ul	190.9 parasites/ul

### January 2020 to December 2024 malaria monthly test positivity rates and trends with interventions in Gulu district and Yumbe district

**Fig 1:**
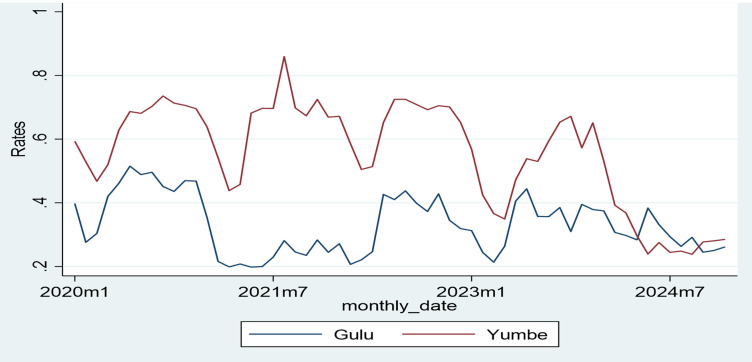
Combined graphical display of five-year (January 2020 to December 2024) rates and trends.

**Fig 2:**
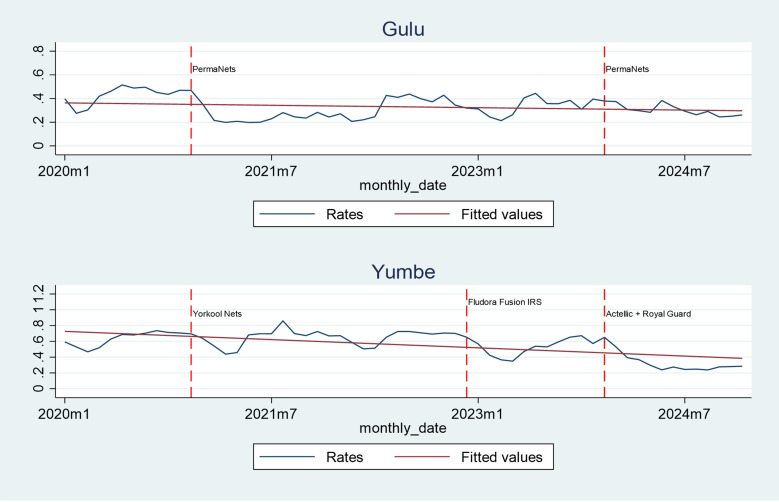
Graphical display of five-year (January 2020 to December 2024) rates and trends mapped with malaria control interventions.

From January 2020 to mid-2023, Yumbe district reported consistently high-test positivity rates compared to Gulu district. Both settings exhibited high seasonal fluctuations in test positivity rate. The noticeable peaks and troughs in both Yumbe and Gulu correspond with high rainfall intensity in the areas. Following the distribution of Yorkool ITNs in Yumbe and PermaNet ITNs in Gulu, there was a dramatic decline in TPR in both settings (Fig 1). In July 2021, Yumbe saw a peak reaching near rates of 0.85 while Gulu remained relatively low during the same period (Fig 2). Following the application of Fludora fusion IRS in Yumbe in December 2022 (Fig 2), there was a significant drop in rates; similar magnitude of reduction within the same time point was observed in Gulu district though there was no mass deployment of malaria control intervention (Figs 1 and 2). From around June 2023, the gaps between the two locations quickly narrowed and even became narrower throughout 2024 following mass distribution of PermaNets in Gulu district and deployment of a combination of Actellic 300 IRS CS+Royal Guard ITNs in Yumbe district in December 2023 (Figs 1 and 2).Summarily, Yumbe started with a much higher and volatile rates but by the end of the data collection period, both settings had declined and stabilized at lower rates.

**Fig 3:**
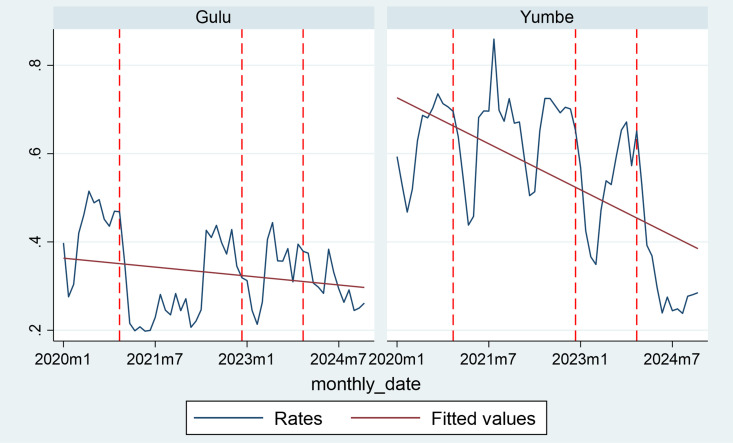
Graph comparing monthly rates and trends with interventions between Gulu and Yumbe districts.

We proceeded with the panel regression analysis using stationary rates because the P value from the Harris-Tzavalis (HT) test was 0.037 (significant) thus no need to convert our rates to first order before proceeding with the regression analysis. The dependent variable was the test positivity rates while the independent variables were time and locality (Table 3, Fig 3).We report a major difference in malaria test positivity rate between Gulu and Yumbe. There was downward trend in both settings though only one was statistically significant. For Gulu district, there was a weak non-significant co-efficient of −0.011; p = 0.093 (Table 3) with a very slow monthly unit dropping rate of only 0.001 making it hard to disprove the argument that the drop could be by chance than attributing it to the impact of interventions (Fig 3). For Yumbe district, we report a significant strong coefficient (−0.0058) with a much faster reduction rate of 0.006 units per month; p < 0.00 (about 5 times faster than Gulu district) (Fig 3, Table 3). About 38.5% of reduction rates can be attributed to the effects of deployment of control interventions. We conclude that the visual fitted values line in Yumbe represent a real reduction while for Gulu is mostly a mathematical average of noisy data (Fig 3).

**Table 3:** Regression analysis for a five year malaria monthly test positivity rates and trends with interventions in Gulu district and Yumbe district.

Location	Coefficient	R-square	t-value	P-value	95% CI
Gulu district	−0.001126	0.0478	−1.71	0.093	−0.0024411 to 0.000194
Yumbe district	−0.0057918	0.3846	−6.02	<0.00	−0.0077175 to −0.0038661

### Climate-adjusted baseline overall interaction trend model

To account for time-varying environmental confounding, a climate-adjusted population-averaged GEE model was fitted (Table 4). After controlling for monthly variation in rainfall, temperature, and relative humidity, the long-term trend interaction term (treated_group#c.monthly_date) remained highly significant (beta = −0.0056, 95% CI: −0.0076, −0.0037,p < 0.001) (Table 4). This indicates that the deployment of malaria vector control interventions in Yumbe District was independently associated with a sustained, continuous reduction in the Malaria Test Positivity Rate of 0.56% per month relative to Gulu district, completely isolated from seasonal climatic dynamics. Mean ambient temperature was found to be a highly significant environmental covariate (beta = −0.0325,p < 0.001) (Table 4).

**Table 4:** Climate-adjusted baseline overall interaction trend model.

Parameter / Covariate	Coefficient (beta)	Robust Standard Error	*z*-statistic	*p*-value
Main Group Context				
Treated Group (Yumbe vs. Gulu)	0.3209	0.0332	9.67	< 0.001
Continuous Time Trend	−0.0005	0.0007	−0.71	0.475
Intervention Impact				
Treated Group (times) Time Trend	−0.0056	0.0010	−5.60	< 0.001
Climatic Confounders				
Monthly Rainfall (mm)	0.0001	0.0001	1.00	0.317
Mean Temperature (°C)	−0.0325	0.0058	−5.60	< 0.001
Relative Humidity (%)	−0.0009	0.0014	−0.64	0.521
Model Constant (beta)	1.2012	0.2070	5.80	< 0.001

### Effectiveness of Yorkool ITNs (Yumbe district) versus PermaNet ITNs (Gulu district) distributed in December 2020

In December 2020, Ministry of Health distributed a new type of ITNs (Yorkool) in Yumbe district while maintaining PermaNets ITNs in Gulu district. To statistically determine which type was more effective, we disaggregated time into three strata and compared positivity rates at pre-intervention (January to November 2020), intervention (December 2020) and post-intervention (January to November 2021). A regression analysis was performed using Generalized Estimation Equation(GEE) population averaged model. Yumbe with a new type was termed “intervention population” while Gulu with the old type was termed “control population”.

For the pre-Intervention Trends _t (Time Trend), our coefficient (0.0129) shows a statistically significant increase in the rate for the Gulu district settings during the ITN pre-distribution period (Table 5). The coefficient (0.1667) of the group difference (_z) indicates that at the start of the study, the intervention setting (Yumbe) had a significantly higher rate than the Gulu setting. Following PermaNet ITN distribution in Gulu and Yorkool ITNs in Yumbe, there was immediate Impact in January 2021 (_x2021m1;Level Change – Control) with significant immediate drop in the rate for the PermaNet ITN population (Gulu). The coefficient (0.0532) for the Yumbe vs.Gulu (_z_x2021m1 Level Change) is not statistically significant suggesting that the dramatic reduction at the start of the intervention was not significantly different between the Yumbe(Yorkool) and Gulu(PermaNet) population (Table 5). We also determined change in trends (Slopes;_x_t2021m1) but reported that control group’s trend changed by, but it was not statistically significant. We also statistically estimated difference in trend Change (_z_x_t2021m1) and found the coefficient (0.0111) to be statistically insignificant. Conclusively, we failed to find any evidence to support the hypothesis that distribution of new types of ITNs (Yorkool) in Yumbe district led to a higher reduction in malaria test positivity rate over time compared to Gulu that maintained PermaNet ITNs (Table 5).

**Table 5:** A regression analysis using Generalized Estimation Equation (GEE) population average model.

Rate	Coefficient	Std. err.	Z	P > |z|	95% lower CI 95% upper CI	
_t	.0128522	.0054069	2.38	0.017	.0022549	.0234495
_z	.1667266	.0460477	3.62	0.000	.0764748	.2569784
_z_t	.0071989	.0076465	0.94	0.346	−.007788	.0221857
_x2021m1	−.2770566	.0530682	−5.22	0.000	−.3810684	−.1730449
_x_t2021m1	−.0123957	.0076465	−1.62	0.105	−.0273825	.0025911
_z_x2021m1	.0531764	.0750498	0.71	0.479	−.0939185	.2002712
_z_x_t2021m1	.0111113	.0108137	1.03	0.304	−.0100833	.0323058
_cons	.3612873	.0325606	11.10	0.000	.2974696	.4251049

**Fig 4:**
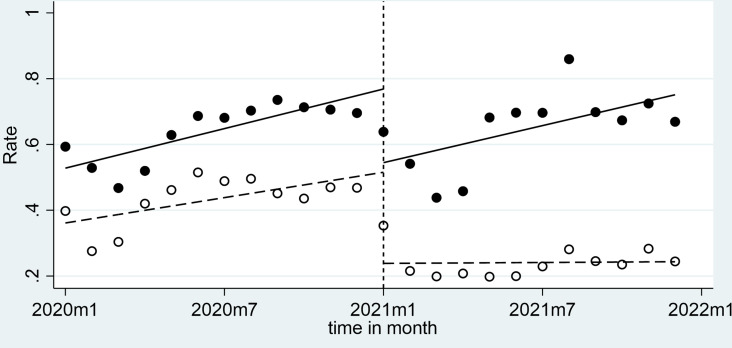
An interrupted time series plot to compare trends in test positivity rate before and after ITN distribution in December 2020 (Yorkool in Yumbe and PermaNet in Gulu). The plot utilizes a Generalized Estimating Equations (GEE) model with a Gaussian family, identity link function, and independent correlation structure. Solid lines represent predicted trends and filled circles indicate actual observed rates for the intervention group (Yumbe), while dashed lines represent predicted trends and hollow circles indicate actual observed rates for the control average(Gulu).

We visually compared trends in test positivity rate before and after ITNs distribution in December 2020 using an interrupted time series plot (Fig 4).The coefficient for the baseline pre-intervention trend;_t (0.0128) suggests that the test positivity rate in the control group (Gulu district) was significantly increasing by approximately 0.013 units per month. We also found the coefficient (0.1667) of the difference in both settings (_z) at the very beginning of the study (month 1) to be relating with a significantly higher rate in Yumbe with Yorkool compared to Gulu with PermaNet. The sudden negative coefficient(−0.2770) during the ITNs distribution phase in both sites (_x2021m1) relates with a highly significant immediate drop in the TPR for the Gulu population in January 2022(Fig 4). This is suggestive of the positive impact of the PermaNet ITNs distributed. The coefficient (−0.0123) for the change in trend (_x_t2021m1) representing the change in the slope for the Gulu population after January 2021 is not significant at the standard 5% level but shows a marginal trend toward the TPR leveling off (Fig 4). We also calculated the intervention effect (Difference-in-Differences). The level change difference (_z_x2021m1) of 0.0531 shows that the dramatic reduction in TPR (January 2021) immediately after ITNs distributions was not significantly different between Yumbe and Gulu(Fig 4). The trend change difference (_z_x_t2021m1) of 0.0111 suggests that the distribution of Yorkool did not significantly change the long-term trend of Yumbe relative to Gulu (Fig 4). We therefore conclude that that the introduction of Yorkool ITNs did not have a unique or different impact on malaria burden in Yumbe District compared to Gulu district that maintained PermaNet ITNs.

### The climate-adjusted model to evaluate the December 2020 milestone

A significant overall step-reduction in TPR was observed post-intervention (beta = −0.0872; p = 0.031) (Table 6). However, the difference-in-differences interaction term (1.treated_group#1.post_2020) was not statistically significant (beta = −0.0054, 95% CI:0.1145, 0.1038, p = 0.923), indicating that the immediate post-2020 divergence between the intervention (Yumbe) and comparison (Yumbe) areas was minimal when holding environmental covariates constant (Table 6).

**Table 6:** Evaluation of Milestone 1: December 2020 Yorkool ITNs Deployment (Model 2).

Parameter / Covariate	Coefficient (beta)	Robust Standard Error	*z*-statistic	*p*-value
**Main Group Context**				
Treated Group (Yumbe vs. Gulu)	0.1675	0.0478	3.50	< 0.001
Post-2020 Policy Period	−0.0872	0.0403	−2.16	0.031
Intervention Impact				
Treated Group (times) Post-2020	−0.0054	0.0557	−0.10	0.923
**Climatic Confounders**				
Monthly Rainfall (mm)	0.0001	0.0002	0.50	0.617
Mean Temperature (°C)	−0.0291	0.0071	−4.10	< 0.001
Relative Humidity (%)	−0.0015	0.0017	−0.88	0.378
Model Constant (beta)	1.2162	0.2620	4.64	< 0.00

### Malaria monthly test positivity rates and trends with deployment of Fludora Fusion IRS in Yumbe (December 2022) and no intervention in Gulu

**Fig 5:**
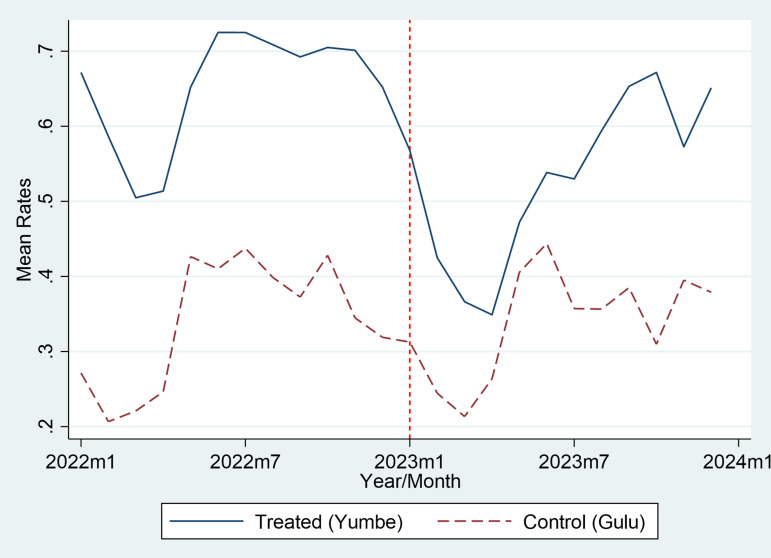
Plot on malaria monthly test positivity rates and trends with deployment of Fludora Fusion IRS in Yumbe (December 2022) and no intervention in Gulu.

The time points were again split into three; pre-intervention(January to November 2022), intervention (December 2022) and post intervention (January 2023 to November 2023). We performed an interaction regression to statistically determine the impact of fludora at different time points. We also plotted graphs from parallel trend assumptions of the difference in difference model. The coefficient (−0.0046682) for the difference in slopes (City_n#c.monthly_date) confirms that Yumbe’s rate fell by an additional 0.0047 units per month compared to Gulu; P-value <0.000 confirming high significance (Table 7, Fig 5). There is strong statistical evidence that Yumbe was declining significantly faster than Gulu and the two districts were not following the same trend. The P value (0.175) for the base city (Gulu) (row monthly_date) is statistically insignificant. Effectively, Gulu’s rate is flat or stable, while Yumbe’s is crashing. The coefficient 3.724 for the starting gap (City_n | Yumbe) shows that Yumbe started with significantly higher rates than Gulu. We therefore conclude that while both cities show a visual downward trend, an interaction model confirms that the decline in Yumbe is significantly steeper than in Gulu

**Table 7:** An interaction regression on malaria monthly test positivity rates and trends with deployment of Fludora Fusion IRS in Yumbe (December 2022) and no intervention in Gulu.

Rates	Coefficient	Std. err.	t	P>|t|	[95% Conf.Interval]	
City_n (Yumbe)	3.724412	.8738816	4.26	0.000	1.99358	5.455245
monthly_date	−.0011236	.0008242	−1.36	0.175	−.0027561	.0005089
City_n#monthly_d	−.0046682	.0011656	−4.00	0.000	−.0069769	−.0023595
_cons (Constant)	1.172229	.6179276	1.90	0.060	−.0516546	2.396112

**Fig 6:**
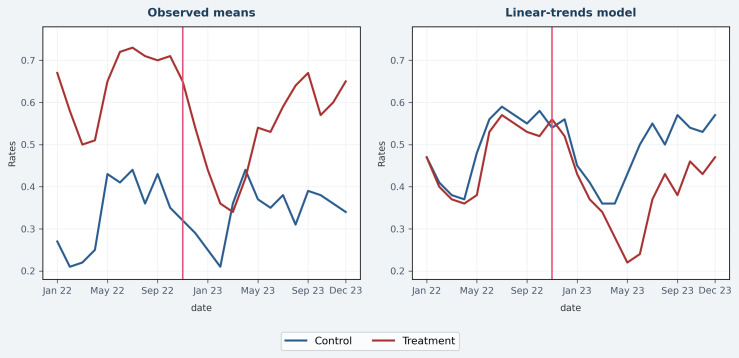
Graphical diagnostics for parallel trends. The standard diagnostic tool graph to evaluate the parallel-trends assumption for Difference-in-Differences (DiD) models, displaying both the observed monthly means (left panel) and the linear-trends model (right panel) from January 2022 through December 2023. Blue lines represent the Control group (Gulu), and red lines represent the Treatment group(Yumbe).

From the standard diagnostic tool graph (Fig 6), we evaluated the parallel-trends assumption for Difference-in-Differences (DiD) models. The observed means (left plot) shows the raw average rates for the intervention(Yumbe) and control group over time (Fig 6). For the pre-treatment (left of the red line): Our DiD model is valid because the blue (Control) and red (intervention) lines follow a similar, parallel path & fluctuations suggesting they were influenced by similar external factors before the application of fludora fusion IRS began. For the post-IRS application, both groups dropped significantly, but Yumbe’s stayed consistently lower than Gulu’s compared to their pre-intervention relationship. This visual gap represents a negative ATET of −0.106. The linear-trends model (Right Plot) is a more formal diagnostic that augments the DiD model with interaction terms to specifically test if the trends are linear and parallel (Fig 6). Summarily, the graphs suggest that the DiD model is largely valid with parallel Trends plots showing that the two districts moved together before the intervention. On impact, there is a distinct, sustained downward shift in the IRS’s rates relative to the control group after the interventions, confirming that fludora fusion significantly reduced malaria burden in Yumbe district (Fig 6).

### The climate-adjusted model evaluation of the December 2022 milestone deployment of Fludora Fusion Indoor Residual Spraying (IRS)

Fludora Fusion demonstrated a profound impact. The difference-in-differences interaction term (1.treated_group#1.post_2022) was highly statistically significant, showing an absolute 21.2% drop in the Malaria Test Positivity Rate in Yumbe District relative to Gulu district (beta = −0.2123, 95% CI: −0.2801, −0.1444, p < 0.001) (Table 8). This strong protective effect was completely isolated from time-varying environmental fluctuations, where ambient temperature remained a significant structural covariate (beta = −0.0266,p < 0.001) (Table 8).

**Table 8:** Evaluation of Milestone 2: December 2022 Fludora Fusion IRS application.

Parameter / Covariate	Coefficient (beta)	Robust Standard Error	*z*-statistic	*p*-value
**Main Group Context**				
Treated Group (Yumbe vs. Gulu)	0.2453	0.0220	11.15	< 0.001
Post-2022 Policy Period	−0.0014	0.0246	−0.06	0.955
**Intervention Impact**				
Treated Group (times) Post-2022	−0.2123	0.0346	−6.14	< 0.001
**Climatic Confounders**				
Monthly Rainfall (mm)	0.0001	0.0001	1.00	0.317
Mean Temperature (°C)	−0.0266	0.0059	−4.51	< 0.001
Relative Humidity (%)	−0.0001	0.0014	−0.07	0.943
Model Constant (beta)	0.9864	0.2100	4.70	< 0.001

### Malaria monthly test positivity rates and trends with simultaneous deployment of Actellic 300 IRS + Royal guard ITNs in Yumbe (December 2023) and a repeat of PermaNet distribution (December 2023) in Gulu district

**Fig 7:**
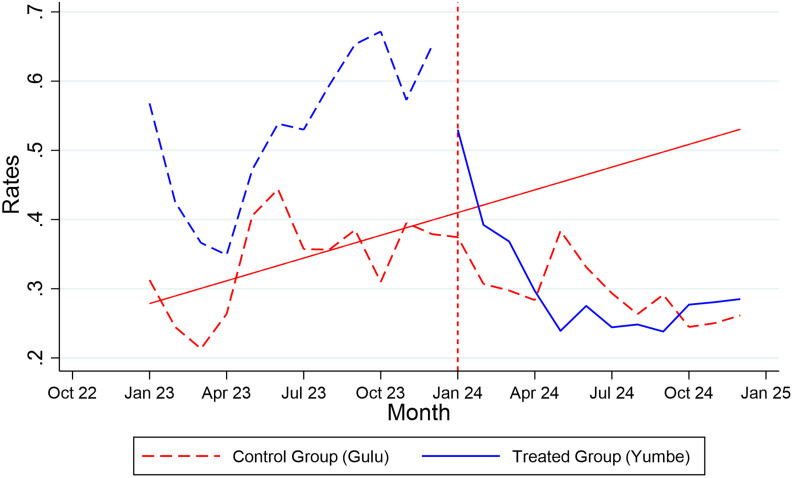
Difference in Difference plot of malaria monthly test positivity rates and trends with simultaneous deployment of Actellic 300 CS IRS + Royal guard ITNs in Yumbe (December 2023) and a repeat of PermaNet distribution (December 2023) in Gulu district. The timeline spans from January 2023 through December 2024. The dashed red line tracks the Control Group (Gulu), and the solid blue line tracks the Treated Group (Yumbe). The vertical red dashed line marks the start of the IRS treatment intervention in January 2024.

We performed a regression analysis to identify the Difference-in-Differences (DiD) effect (Table 9, Fig 7). Because of having only two groups (clusters), we used a standard OLS regression (reg) with an interaction term to get the estimate. The DiD Coefficient (−0.1813;the interaction term 1.is_treated_group#1.post_period) indicates that simultaneous deployment of Actellic 300 IRS and Royal Guard ITNs was associated with a 0.1813 unit decrease in malaria test positivity rate in Yumbe district relative to Gulu district, after controlling for baseline differences and time trends. This decrease was statistically significant because the P-value was 0.003 at 1% level (Table 9, Fig 7). The coefficient −0.0436 (1.is_treated_group) shows that before the intervention, Yumbe had rates 0.0436 units lower than Gulu (not statistically significant,).The coefficient 0.1044 (1.post_period) shows that in the Post period, TPR in the Gulu population increased by 0.1044 units compared to its own baseline/pre-intervention rate of 0.3835 (_cons) (Table 9, Fig 7).

**Table 9:** Regression analysis of malaria monthly test positivity rates and trends with simultaneous deployment of Actellic 300 IRS + Royal guard ITNs in Yumbe (December 2023) and a repeat of PermaNet distribution (December 2023) in Gulu district.

Rates	Coefficient	Std. err.	t	P>|t|	[95% conf. interval]	
1.is_treated_group	−.0436357	.0401109	−1.09	0.283	−.124527	.0372556
1.post_period	1044281	.0401109	2.60	0.013	.0235368	.1853194
is_treated_group#post_period						
1 1	−.1813122	.0573664	−3.16	0.003	−.2970025	−.0656219
_cons	.3835375	.0283627	13.52	0.000	.3263387	.4407363

**Fig 8:**
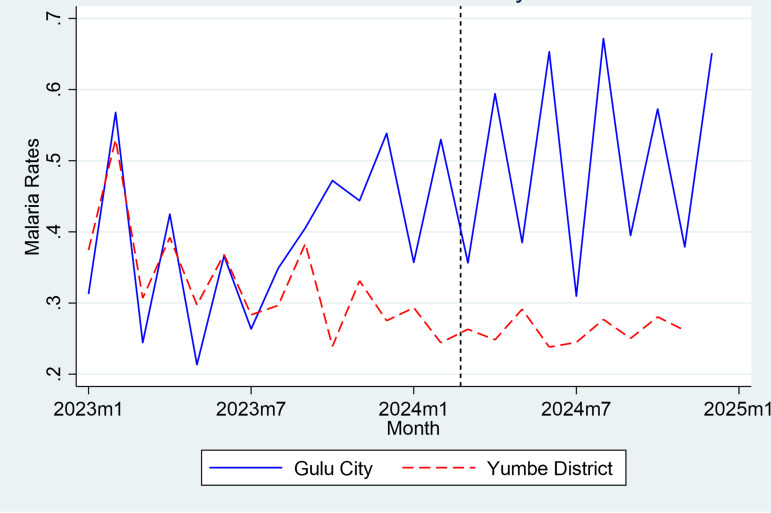
Parallel trends analysis. A line graph tracking monthly malaria rates from January 2023 (2023m1) through January 2025 (2025m1) to evaluate the parallel trends assumption between Gulu City (solid blue line) and Yumbe District (dashed red line). The vertical black dashed line indicates the timing of the intervention implementation in January 2024.

We also performed a parallel trends assumption test and plotted parallel trend graphs to address the challenge of OLS (because of few clusters) underestimating the standard error in DiD which makes the results look more significant than they actually are (Fig 8). We found the Average Treatment Effect on the Treated (ATET) to be −0.1664675 suggesting that the intervention (Actellic 300 IRS+Royal guard ITNS) was associated with an additional 16.6 percentage point reduction in malaria test positivity rates in Yumbe district compared Gulu district. From the plot, we noted that between January to July 2023 (pre-intervention months), the two lines (Gulu district and Yumbe District) tracked each other remarkably well, exhibiting the same sawtooth seasonal pattern, moving up and down in unison (Fig 8). This is strong visual evidence that the Parallel trends assumption holds for the first half of the pre-treatment year. However, we noted that from August 2023 (three months before deployment of IRS and ITNS), Gulu district’s rates started an upward climb while Yumbe District’s rates began a steady decline. In the post-intervention, the divergence becomes even more pronounced (Fig 8). Gulu district remains extremely volatile with very high peaks, while Yumbe District stabilizes at a much lower rate suggesting a significant negative effect (ATET of −0.166). This graph supports the finding that Yumbe District with ITN + IRS maintained lower and more stable malaria rates compared to the rising volatility in Gulu district with only ITNs (Fig 8).

### The climate-adjusted model evaluation of the December 2023 milestone, the co-deployment of Actellic 300 IRS and Royal Guard dual-active ingredient ITNs

The simultaneous deployment of both interventions yielded the highest protective effect. The difference-in-differences interaction term (1.treated_group#1.post_2023) was highly statistically significant, demonstrating an absolute 25.1% drop in the Malaria Test Positivity Rate in Yumbe District compared to Gulu district (beta = −0.2508, 95%CI:-0.3279, −0.1737,p < 0.001). This confirms a compounding benefit over the previous single-strategy interventions. Ambient temperature remained a highly significant covariate (beta = −0.0231,p < 0.001), while rainfall and relative humidity showed no independent significant association with TPR changes within this phase (Table 10).

**Table 10:** Evaluation of Milestone 3: December 2023 Royal Guard ITN Co-deployment.

Parameter / Covariate	Coefficient (beta)	Robust Standard Error	*z*-statistic	*p*-value
Main Group Context				
Treated Group (Yumbe vs. Gulu)	0.2172	0.0192	11.31	< 0.001
Post-2023 Policy Period	−0.0239	0.0281	−0.85	0.395
**Intervention Impact**				
Treated Group (times) Post-2023	−0.2508	0.0393	−6.38	< 0.001
**Climatic Confounders**				
Monthly Rainfall (mm)	0.0001	0.0001	1.00	0.317
Mean Temperature (°C)	−0.0231	0.0056	−4.13	< 0.001
Relative Humidity (%)	0.0003	0.0013	0.23	0.817
Model Constant (beta)	0.8749	0.2013	4.35	< 0.001

## Discussion

This study measured the impact of compounds used in different ITN types and IRS campaigns, different permutations of IRS + ITNs on malaria test positivity rates in high disease transmission settings of Yumbe district and Gulu district.

For the five-year malaria trend analysis, test positivity rates in Yumbe district dropped five times faster than that of Gulu district (p < 0.00) with 38.5% of reduction rates in Yumbe attributable to the deployment of different combinations of control interventions. Our findings is similar to that of a four years retrospective study done in Eritrea on the impact of combinations of control interventions on malaria test positivity rates [15]. They reported that malaria morbidity was strongly correlated to the number of ITNs distributed and the amount of DDT and malathion used in IRS. Our study and the Eritrean study utilized secondary data to evaluate the combined impact of indoor residual spraying and insecticide-treated nets, demonstrating that co-deployment of these vector control strategies yields a superior reduction in malaria burden compared to single interventions [15]. Finding of our study is different from that of a cross-sectional study conducted in selected urban and rural communities in Benin [17]. While a combination of malaria control strategy was preferred in Uganda, the Benin study reported that for different protection rates in urban and rural areas. For them, sleeping under ITNs in urban areas did not confer significant protection against malaria and uncomplicated malaria compared to no intervention at all. They further reported that in rural areas, the use of ITNs alone, IRS alone or a combination of the two did not provide additional protection compared to no intervention at all [17].

Our study also reported that the introduction of a new type of ITNs (Yorkool) in December 2020 campaign did not significantly change the long-term trend in TPR of Yumbe district relative to Gulu district that maintained distribution of PermaNets ITN for the same time point (The trend change difference of 0.0111). Our findings have been significantly different from a WHO report which found Clinical trials and pilot studies on dual-insecticide nets to improve malaria control by 20–50% compared with standard pyrethroid-only nets. Our finding was also different from additional WHO report that clinical trials in the United Republic of Tanzania and Benin demonstrated that the pyrethroid-chlorfenapyr nets significantly reduced malaria infections in children between the ages of 6 months and 10 years. This difference could be embedded in the burden of asymptomatic malaria and environmental drainage systems. Yumbe district is on the northern border of the western river Nile, so the drainage system is good with less swamps, whereas Gulu district is fairly flat land with swampy river banks [18]. Yumbe district also had a lower burden of asymptomatic malaria among the vulnerable groups compared to Gulu [19,20]. Conversely, our findings regarding the limited long-term impact of single net transitions mirror recent interrupted time series evidence published by Epstein et al. (2025) [5] using routine facility data across Uganda. They reported that while next-generation insecticidal net distributions achieved an initial 23% reduction in malaria incidence during the first 12 months, this protective benefit was completely lost by months 13–24 due to rapid physical or insecticidal attrition. Similar operational drops in effectiveness have been documented by Zhou et al. [21] across West Africa when switching to newer active ingredients without precisely matching them to local vector dynamics. Consequently, programmatic vector control strategies cannot merely focus on the coverage scale of deployment; they must dynamically align the specific chemical mechanisms of both IRS campaigns and ITN types with evolving local entomological resistance profiles to avoid cross-resistance and maximize community protection

We also report that Fludora Fusion demonstrated a profound impact. The difference-in-differences interaction term was highly statistically significant, showing an absolute 21.2% drop in the Malaria Test Positivity Rate in Yumbe District relative to Gulu district (beta = −0.2123, 95% CI: −0.2801, −0.1444, p < 0.001). Following a co-deployment of Actellic 300 CS IRS+ Royal Guard ITNs in Yumbe and distribution of PermaNet ITNs in Gulu, the difference-in-differences interaction term was highly statistically significant, demonstrating an absolute 25.1% drop in the Malaria Test Positivity Rate in Yumbe District compared to Gulu district (beta = −0.2508, 95%CI:-0.3279, −0.1737,p < 0.001). The varying impact of the vector control measures observed in this study underscores that the operational success of combined interventions is heavily shaped by the specific insecticide classes deployed. In Uganda, widespread vector resistance to standard pyrethroids severely complicates control efforts. Our observations closely align with a landmark longitudinal study by Nankabirwa et al. [22] in Tororo, Uganda, which demonstrated that switching the active IRS insecticide compound from pyrethroids to long-lasting organophosphates (such as pirimiphos-methyl) yielded a significantly deeper, sustained reduction in laboratory-confirmed malaria morbidity. This chemical reality explains why transitioning to newer-generation dual-insecticide tools, or co-deploying distinct chemical classes such as combining the organophosphate compound Actellic 300 in IRS campaigns with the dual-active ingredients in Royal Guard ITNs yielded a significantly more pronounced reduction in test positivity rates than single-chemical interventions. Our findings agree with that of Echodu R. et al. [13] Crucially, before introducing chemoprophylaxis, their study isolated and quantified the impact of population-based indoor residual spraying combined with ITNs, reporting a 97.6% decrease in malaria incidence and a 96% decrease in parasite prevalence after several rounds of IRS. When chemoprophylaxis was subsequently added as a separate arm, an even more drastic decrease of up to 99.5% was observed. Because our study similarly focused on the vector control baseline prior to pharmaceutical interventions, the IRS-only data from Echodu et al. serves as a highly comparable and geographically relevant baseline [13].

## Conclusion

In areas of high malaria transmission, deployment of either ITNs alone, IRS alone, or in combination can be an effective tool against malaria mosquito vectors. However, a more sustained and significant reduction is achieved through the simultaneous deployment of IRS and ITNs. Crucially, the efficacy of these combined interventions is highly shaped by the specific classes of insecticides and active compounds utilized within the deployed ITN types and IRS campaigns.

### Study limitation

The DHIS2 data reviewed, analyzed and used in this article was not standardized for research and could possibly lack completeness. To minimize potential environmental bias, our adjusted time-series analysis fully accounted for local meteorological confounders, including monthly rainfall patterns, mean ambient temperature, and relative humidity. Because both Yumbe and Gulu districts share highly comparable baseline climate profiles and malaria transmission intensities, adjusting for these weather dynamics isolates the true operational effect of the vector control campaigns. Furthermore, while some routine laboratory tests were initially conducted by non-WHO qualified staff, we systematically addressed this data reliability limitation by utilizing Rapid Diagnostic Test (RDT) results instead of routine microscopy.

## Supporting information

S1 Data Comprehensive secondary malaria surveillance dataset. Anonymized Excel spreadsheet containing the historical monthly malaria data metrics for Gulu and Yumbe districts used for statistical modeling.
(XLS)
