# Supplementary material for: Combination of different intervention strategies for malaria mosquito vector control in Uganda: A review of secondary data of two districts with moderate to high disease transmission
Source: PLoS One. 2026 Jul 6;21(7):e0352455. doi: 10.1371/journal.pone.0352455 (PMC13336156; doi:10.1371/journal.pone.0352455)
Supplement: Attachment — Submitted filename: Reviewer Comments on Manuscript Number PONE-D-26-11202.pdf [file pone.0352455.s002.pdf]

Reviewer Recommendation and Comments for Manuscript Number PONE-D-26-11202

The Authors assessed how different IRS insecticide formulations and ITN types, used alone or in combination, influence malaria test positivity rates in two high-burden districts in Uganda. Studies on the effectiveness of ITNs and/or IRS remain relevant to guide malaria control decisions. However, the manuscript needs to be revised to strengthen the analysis and improve the presentation, interpretation, and discussion of the results.

Major Comments

Given that mosquito ecology and malaria transmission dynamics are strongly influenced by key environmental factors such as rainfall, temperature, humidity, and vegetation indices, I find it concerning that the authors did not account for any climatic factors in their models, particularly in the interrupted time series analysis.

The justification that these data were not readily available is not entirely accurate. Climatic and environmental datasets are widely accessible through remote sensing platforms and could be incorporated into the analysis. For example, temperature and rainfall data can be obtained from sources such as the TerraClimate and CHIRPS, respectively, while vegetation indices like NDVI are available from the Landsat Surface Reflectance-derived-spectral-indices website.

Failing to account for these time-varying factors introduces a high risk of confounding and makes it difficult to attribute observed changes to the interventions. This also limits the validity of the conclusions.

I have also highlighted some concerns in various parts of the manuscript. Here is a summary of some additional issues:

1. The authors consider inter-ITN campaign periods as “no intervention.” This is incorrect because WHO-prequalified ITNs typically remain effective for about 3 years, except in specific cases such as PBO ITNs (with reported durability that averages about 18-24 months in some studies), and their protective effect is expected to last throughout the nets' lifetime. Therefore, observations during these periods should not be classified as no intervention, contrary to the current approach. It is also not clear how the authors incorporated interventions or accounted for intervention coverage in their models.

2. The parallel trends assumption appears uncertain (particularly in Fig 8). A formal pre-trend test (e.g. treatment × time interaction restricted to the pre-intervention period) should be conducted and reported.

3. The discussions need revision to strengthen the paper. In this version, the authors mainly compare their findings with other studies but offer limited interpretation and minimal discussion of the implications. From the current analysis and results presented it is clear that the effectiveness of interventions is heavily influenced by the insecticide class and formulation or ITN type. I feel this is an important point that should be emphasised.

4. One of the main findings is that IRS with Fludora fusion contributed to declines in malaria cases in this study area. This reported impact of Fludora Fusion contrasts with findings from other settings in Uganda (https://doi.org/10.1371/journal.pgph.0003254; https://doi.org/10.1371/journal.pgph.0000676 and https://doi.org/10.21203/rs.3.rs-7518583/v1) where clothianidin-based IRS has shown limited impact. I think this is a significant find, assuming the results do not change after the

recommended revisions/analysis and should be discussed in depth. The authors should (i) situate findings within the broader literature, ii) explore possible explanations (e.g. prior IRS exposure, ecological differences) iii) report and interpret the magnitude of effect more explicitly.

5. It is unclear whether Gulu refers to an entire district or a single city. The authors have used city and district interchangeably. This creates some confusion and raises concerns about comparability. We cannot compare data from a district and that of a township/city.

<u>Minor comments</u>:

6. The Results section includes elements of study description and discussion. These should be moved to the appropriate sections to maintain a clear and logical structure.

7. A few observations on terminology, consistency, and tone
    - Terminology should be standardised throughout the manuscript. The authors should consistently use either active ingredients, product formulations, or class-level terms, rather than mixing these. For example, alternating between Actellic 300CS and pirimiphos-methyl, or SumiShield/Fludora and clothianidin/deltamethrin, creates confusion. Similarly, there is inconsistency in the level of classification used. In some sections, chemical classes (e.g. “pyrethroid”) are presented alongside specific active ingredients (e.g. “pyriproxyfen”). This should be standardised to either class-level or active-ingredient-level terminology for clarity and consistency

    - Authors should consider using either “ITNs” (recommended) or “LLINs” consistently throughout. And should also avoid repeated definitions of IRS and ITNs once introduced, and ensure abbreviations are used consistently after definition.

    - Replace words phrasing like “mass scale IRS applications” with standard terminology, either as IRS campaigns or mass ITN distribution

8. Figures
    - Figure 1: Authors should consider using distinct colour shading especially for Gulu and Yumbe.

    - Figure 2A:
        1. Use clearer distinctions between intervention periods (e.g. colour and line type).
        2. Define “M1” and “M7” in a footnote and also add footnotes indicating timing of ITN distributions.

    - Figure 8 looks similar to Figure 7. It could be helpful for authors to clarify how they differ or to consider consolidating them if they are redundant.

9. Tables
    - Table 4: revise headings to “Lower 95% CI” and “Upper 95% CI”.
    - It would be helpful to standardise decimal places across all tables and text.

Kindly see the manuscript for additional comments.
