# Supplementary material for: Combination of different intervention strategies for malaria mosquito vector control in Uganda: A review of secondary data of two districts with moderate to high disease transmission
Source: PLoS One. 2026 Jul 6;21(7):e0352455. doi: 10.1371/journal.pone.0352455 (PMC13336156; doi:10.1371/journal.pone.0352455)
Supplement: Attachment — Submitted filename: PONE-D-26-11202_Review Feedback.pdf [file pone.0352455.s003.pdf]

PLOS One

Combination of different intervention strategies for Malaria Mosquito vector control in Uganda; a review of secondary data of two districts with moderate to high disease transmission.

--Manuscript Draft--

Manuscript Number:	PONE-D-26-11202
Article Type:	Research Article
Full Title:	Combination of different intervention strategies for Malaria Mosquito vector control in Uganda; a review of secondary data of two districts with moderate to high disease transmission.
Short Title:	Combination of malaria control strategies in Uganda
Corresponding Author:	Erick Jacob Okek, MSc<br>Makerere University Faculty of Medicine: Makerere University College of Health Sciences<br>Kampala, Kampala UGANDA
Keywords:	IRS, ITNS, TPR, Yumbe, Gulu
Abstract:	Background<br><br>Indoor residual spraying (IRS) and insecticide treated bed nets (ITNs) used either singly or in combination are the main mass mosquito vector control measures for malaria control. Despite their widespread use, malaria transmission rates remain high, the burden unacceptably huge, and yet the disease is completely preventable. This study measured the impact of compounds used in different brands of IRS and ITNS, different permutations of IRS+ITNS on malaria test positivity rates in high disease transmission settings of Yumbe district and Gulu city.<br><br>Method<br><br>The DHIS 2 records on distribution of ITNs, IRS schedules and malaria cases by gender, age and geographical location (Gulu and Yumbe districts) collected over a five-year period were used in the final analysis. Data collection was done using a checklist developed in an excel spreadsheet. Data on the following were extracted; socio-demographic characteristics, number of monthly malaria tests, monthly numbers of positive malaria tests, the brand of Insecticide Treated Nets (ITNS) distributed, the brand of Indoor Residual Spray (IRS) applied and the time points that the interventions were deployed. The data was exported into STATA ver 17.0,cleaned and additional variables generated prior to interrupted time series and Difference in Difference analysis. Monthly malaria test positivity rate (TPR) was calculated. A regression analysis and graphical plots using Generalized Estimation Equation (GEE) population average model was performed to assess the impact of deployment of a combination of control interventions on TPR at different time points.<br><br>Results<br><br>For the five-year malaria trend analysis, TPR in Yumbe district reported a much faster reduction rate of 0.006 units per month; p<0.00 (about 5 times faster than Gulu City) with about 38.5% of reduction rates attributable to variation in either time or deployment of control interventions. The trend change difference of 0.0111 suggests that the distribution of the new brand of ITNS (Yorkool) in December 2020 did not significantly change the long-term trend in TPR of Yumbe relative to Gulu city that maintained distribution of PermaNets ITN for the same time point. Following the application of Fludora fusion IRS in Yumbe district in December 2022 and no mass scale deployment of any control intervention in Gulu city for the same time point, the coefficient of -0.004668 for the difference in slopes confirms that Yumbe's rate significantly fell by an additional 0.0047 units per month compared to Gulu; P-value <0.000. Following a co-deployment of Acetellic 300 IRS+ Royal Guard ITNS in Yumbe district and distribution of PermaNet ITNS in Gulu city, we found the Average Treatment Effect on the Treated (ATET) to be -0.1664675 suggesting that after

Powered by Editorial Manager® and ProduXion Manager® from Aries Systems Corporation

	adjusting for panel and time effects, the intervention (Acetellic 300 IRS+Royal guard ITNS) is associated with a 16.6 percentage point reduction in malaria test positivity rates in Yumbe district compared to Gulu City<br><br>Conclusion<br><br>In areas of high malaria transmission, deployment of either ITNs alone, IRS alone or in combination can be an effective tool against malaria mosquito vector control .However, a more sustained reduction can only be achieved through simultaneous deployment of IRS+ITNS
**Order of Authors:**	Erick Jacob Okek, MSc
	Loyce Nakalembe, MSc,BSc
	Silvia Awor
	Benson Musinguzi
	Amos Drasiku
	Sande James Obondo, PhD,MMed, MBChB
	Martin Lukindu
	Julius Lutwama, PhD,MSc,BSc
	Jonathan Kayondo, PhD,MSc,BSc
	Moses Ocan, PhD,MSc,BSc
	Winnie Nambatya, Bpharm, MSc
**Additional Information:**	
**Question**	**Response**
**Financial Disclosure**<br><br>Enter a financial disclosure statement that describes the sources of funding for the work included in this submission. Review the submission guidelines for detailed requirements. View published research articles from *PLOS ONE* for specific examples.<br><br>This statement is required for submission and **will appear in the published article** if the submission is accepted. Please make sure it is accurate.	The author(s) received no specific funding for this work.

Powered by Editorial Manager® and ProduXion Manager® from Aries Systems Corporation

**Funded studies**<br>Enter a statement with the following details:<br>• Initials of the authors who received each award<br>• Grant numbers awarded to each author<br>• The full name of each funder<br>• URL of each funder website<br>• Did the sponsors or funders play any role in the study design, data collection and analysis, decision to publish, or preparation of the manuscript?<br><br>Did you receive funding for this work?	
**Competing Interests**<br><br>Use the instructions below to enter a competing interest statement for this submission. On behalf of all authors, disclose any competing interests that could be perceived to bias this work—acknowledging all financial support and any other relevant financial or non-financial competing interests.<br><br>This statement is required for submission and **will appear in the published article** if the submission is accepted. Please make sure it is accurate and that any funding sources listed in your Funding Information later in the submission form are also declared in your Financial Disclosure statement.<br><br>View published research articles from *PLOS ONE* for specific examples.	Not applicable

**NO authors have competing interests**<br><br>Enter: *The authors have declared that no competing interests exist.*<br><br>**Authors with competing interests**<br><br>Enter competing interest details beginning with this statement:<br><br>*I have read the journal's policy and the authors of this manuscript have the following competing interests: [insert competing interests here]*<br><br>* typeset	
**Ethics Statement**<br><br>Enter an ethics statement for this submission. This statement is required if the study involved:<br><br>• Human participants<br>• Human specimens or tissue<br>• Vertebrate animals or cephalopods<br>• Vertebrate embryos or tissues<br>• Field research<br><br>Write "N/A" if the submission does not require an ethics statement.<br><br>General guidance is provided below. Consult the submission guidelines for detailed instructions. **Make sure that all information entered here is included in the Methods section of the manuscript.**	N/A; this was the review of secondary data generated at a population level

**Format for specific study types**<br><br>**Human Subject Research (involving human participants and/or tissue)**<br>• Give the name of the institutional review board or ethics committee that approved the study<br>• Include the approval number and/or a statement indicating approval of this research<br>• Indicate the form of consent obtained (written/oral) or the reason that consent was not obtained (e.g. the data were analyzed anonymously)<br><br>**Animal Research (involving vertebrate animals, embryos or tissues)**<br>• Provide the name of the Institutional Animal Care and Use Committee (IACUC) or other relevant ethics board that reviewed the study protocol, and indicate whether they approved this research or granted a formal waiver of ethical approval<br>• Include an approval number if one was obtained<br>• If the study involved *non-human primates*, add *additional details* about animal welfare and steps taken to ameliorate suffering<br>• If anesthesia, euthanasia, or any kind of animal sacrifice is part of the study, include briefly which substances and/or methods were applied<br><br>**Field Research**<br><br>Include the following details if this study involves the collection of plant, animal, or other materials from a natural setting:<br>• Field permit number<br>• Name of the institution or relevant body that granted permission	
**Data Availability**<br><br>Authors are required to make all data underlying the findings described fully available, without restriction, and from the time of publication. PLOS allows rare exceptions to address legal and ethical concerns. See the PLOS Data Policy and FAQ for detailed information.	Yes - all data are fully available without restriction

Powered by Editorial Manager® and ProduXion Manager® from Aries Systems Corporation

A Data Availability Statement describing where the data can be found is required at submission. Your answers to this question constitute the Data Availability Statement and **will be published in the article**, if accepted.<br><br>**Important:** Stating 'data available on request from the author' is not sufficient. If your data are only available upon request, select 'No' for the first question and explain your exceptional situation in the text box.<br><br>Do the authors confirm that all data underlying the findings described in their manuscript are fully available without restriction?	
**Describe where the data may be found in full sentences. If you are copying our sample text, replace any instances of XXX with the appropriate details.**<br><br>• If the data are **held or will be held in a public repository**, include URLs, accession numbers or DOIs. If this information will only be available after acceptance, indicate this by ticking the box below. For example: *All XXX files are available from the XXX database (accession number(s) XXX, XXX.*).<br>• If the data are all contained **within the manuscript and/or Supporting Information files**, enter the following: *All relevant data are within the manuscript and its Supporting Information files.*<br>• If neither of these applies but you are able to provide **details of access elsewhere**, with or without limitations, please do so. For example:<br><br>*Data cannot be shared publicly because of [XXX]. Data are available from the XXX Institutional Data Access / Ethics Committee (contact via XXX) for researchers who meet the criteria for access to confidential data.*<br><br>*The data underlying the results presented in the study are available from (include the name of the third party*	Full data has been attached in this submission

and contact information or URL).<br>• This text is appropriate if the data are owned by a third party and authors do not have permission to share the data.<br><br>* typeset	
Additional data availability information:	

Manuscript

Click here to access/download;Manuscript;DHIS 2_improved_comprehensive analysis 1.docx

Combination of different intervention strategies for Malaria Mosquito vector control in Uganda; a review of secondary data of two districts with moderate to high disease transmission.

Erick. Jacob Okek [1,4,5], Winnie Nambatya[2] ,Loyce Nakalembe[2], Silvia Awor [6], Benson Musinguzi [1,7], Amos Drasiku [3,7] Sande James Obondo [1], Martin Lukindu [4], Julius Lutwama [5] , Jonathan Kayondo[4] , Moses Ocan [2]

1. Department of Immunology & Molecular Biology, School of Biomedical Sciences, College of Health Sciences, Makerere University, P.O box 7072, Kampala, Uganda
2. Department of Pharmacology & Therapeutics, School of Biomedical Sciences, College of Health Sciences , Makerere University, P.O box 7072, Kampala, Uganda
3. Department of Epidemiology & Biostatistics, School of Medicine, College of Health Sciences, Makerere University, P.O box 7072, Kampala, Uganda
4. Department of Entomology & Parasitology, Uganda Virus Research Institute, P.O box 49, Entebbe, Uganda
5. Department of Arbovirology & zoonotic, Uganda Virus Research Institute, P.O box 49, Entebbe, Uganda
6. Department of Reproductive Health, Faculty of Medicine, Gulu University, P.O. Box 166, Laroo Division, Gulu
7. Department of Medical Laboratory Sciences, Faculty of Health Sciences, Muni University, P.O. Box 725 Arua, Uganda
8. Department of Pharmacology, Soroti University, P.O Box 211, Soroti, Uganda

Corresponding Author: Erick Jacob Okek

okekerick@yahoo.com

Abstract:

Background: Indoor residual spraying (IRS) and insecticide treated bed nets (ITNs) used either singly or in combination are the main mass mosquito vector control measures for malaria control. Despite their widespread use, malaria transmission rates remain high, the burden unacceptably huge, and yet the disease is completely preventable. This study measured the impact of compounds used in different brands of IRS and ITNS, different permutations of IRS+ITNS on malaria test positivity rates in high disease transmission settings of Yumbe district and Gulu city.

Method: The Ministry of Health's District Health Information System 2 (DHIS 2) records on distribution of ITNs, IRS schedules and malaria cases by gender, age and geographical location (Gulu and Yumbe districts) collected over a five-year period (DHIS 2 records accessed on 11th February 2025) were used in the final analysis. Data collection was done using a checklist developed in an excel spreadsheet. Data on the following were extracted; socio-demographic characteristics, number of monthly malaria tests, monthly numbers of positive malaria tests, the brand of Insecticide Treated Nets (ITNS) distributed, the brand of Indoor Residual Spray (IRS) applied and the time points that the interventions were deployed. The data was exported into STATA ver 17.0,cleaned and additional variables generated prior to interrupted time series and Difference in Difference analysis. Monthly malaria test positivity rate (TPR) was calculated. A regression analysis and graphical plots using Generalized Estimation Equation (GEE) population average model was performed to assess the impact of deployment of a combination of control interventions on TPR at different time points.

Results: For the five-year malaria trend analysis, TPR in Yumbe district reported a much faster reduction rate of 0.006 units per month; p<0.00 (about 5 times faster than Gulu City) with about 38.5% of reduction rates attributable to variation in either time or deployment of control interventions. The trend change difference of 0.0111 suggests that the distribution of the new brand of ITNS (Yorkool) in December 2020 did not significantly change the long-term trend in TPR of Yumbe relative to Gulu city that maintained distribution of PermaNets ITN for the same time point. Following the application of Fludora fusion IRS in Yumbe district in December 2022 and no mass scale deployment of any control intervention in Gulu city for the same time point, the coefficient of -0.004668 for the difference in slopes confirms that Yumbe's rate significantly fell by an additional 0.0047 units per month compared to Gulu; P-value <0.000. Following a co-deployment of Acetellic 300 IRS+ Royal Guard ITNS in Yumbe district and distribution of PermaNet ITNS in Gulu city, we found the Average Treatment Effect on the Treated (ATET) to be -0.1664675 suggesting that after adjusting for panel and time effects, the intervention

(Acetellic 300 IRS+Royal guard ITNS) is associated with a 16.6 percentage point reduction in malaria test positivity rates in Yumbe district compared to Gulu City

Conclusion: In areas of high malaria transmission, deployment of either ITNs alone, IRS alone or in combination can be an effective tool against malaria mosquito vector control .However, a more sustained reduction can only be achieved through simultaneous deployment of IRS+ITNS

INTRODUCTION:

WHO projects that the key 2030 malaria indicator morbidity and mortality milestones outlined in the Global Technical strategy of 2016 to 2030 will be missed by 89% and 88% respectively if changes in Malaria control are not implemented immediately[1]. To achieve this, they recommended a universal coverage of either Indoor residual spray (IRS) or Insecticide Treated Nets (ITNs) as core mass malaria mosquito vector control interventions[2]. Other methods that target reduction in parasite transmission and elimination are Intermittent Preventive Therapy (IPT), mass drug administration and recently vaccines have been added to the arsenal for malaria control[3].

Protective efficacy of ITNS have been reported to be greater in high transmission settings when utilized for a longer period of time[4] .A study by *Epstein A et al 2025* [5] reported that malaria incidence in 64 sites in Uganda that received ITNs decreased from 827 cases per 1000 person years in the pre-distribution phase to 538 cases per 1000 person years in the post distribution period[5] demonstrating the effectiveness of this intervention. In many malaria endemic countries, ITNS are mostly distributed by authorities as part of a wider malaria control campaign[6]. Routinely, there are targeted distribution to pregnant mothers during antenatal visits and to infants during immunizations. With over 80% community coverage and at least one net per two persons, this intervention alone can sufficiently protect against new Malaria infections[7]. In 2010, Ugandan Ministry of Health distributed ITNS to pregnant women only. In 2013-2014, 2017-2018 and 2020-2021, there was mass scale distribution of ITNS to all population at risk in all regions of the country[8] achieving a significantly higher coverage of up to 94.1% after the 2020/2021 distribution cycle.

Indoor residual spraying (IRS) was re-introduced in Uganda in 2006 following decades of suspension. Ten districts in Northern Uganda with high perennial malaria transmission at the time (including Gulu and Yumbe) were selected for the first phase of implementation[9]. Between 2006 to 2014, three rounds of IRS using a combination of different compounds such as lambda cyhalothrin, alpha-cypermethrin, bendiocarb and pirimiphos-methyl were applied across the ten districts. Each round was followed by an immediate sharp reduction in cases then a dramatic resurgence at few months after the application. Mass IRS was eventually stopped in some districts due to logistical factors and non-satisfactory performance[10]. Among regions that sustained implementation of

IRS was Eastern Uganda; specifically, Tororo district. *Kamya MR et al 2024* [11]documented trends in selected malaria metrics at different time points of IRS implementation in Tororo district from October 2011 to September 2023. They reported a remarkable reduction in cases and parasite prevalence between 2015 to 2020 when Actellic 300 and Bendiocarb were the compounds in use for the IRS. This was followed by a dramatic resurgence between April 2020 to March 2023 when the compounds were changed to Fludora Fusion and Sumishield. When it was reverted to Actellic 300, cases immediately reduced[11]. The trend clearly indicates that the effectiveness of IRS is dependent on many external factors, including the compound of choice and associated mosquito vector resistance pattern.

Many countries have chosen to simultaneously deploy a combination of ITNS and IRS in order to achieve the 2030 WHO malaria elimination target [12]. By January 2024, thirty-one out of 57 sub-Saharan African countries were implementing a co-deployment of IRS and ITNS for malaria control despite contrasting reports on the effectiveness of such a combination[13]. *Echodu.R. et al* reported that implementation of IRS combined with ITNs was associated with 97.6% decrease in malaria incidence and 96% decrease in parasite prevalence, both measured after several rounds of IRS. However, when chemoprophylaxis is added, there was an even more drastic decrease in malaria incidence of up to 99.5%[14]. To the contrary, a randomized controlled trial in Benin found no added protective effects in the deployment of IRS+ITNS over bed nets alone[15]. A four-year prospective study that evaluated the National Malaria control program in Eritrea found no added advantage of using IRS and ITNs as opposed to using either method alone[16]. An observational study conducted in Ethiopia reported that while both intervention arms (PBO nets alone versus Standard ITNS+IRS) were associate with reduced malaria cases, there was a difference in sustained effects; with ITNS+IRS lasting longer[17].

Despite conflicting reports on the impact of multiple interventions on Malaria control, Uganda is already administering R21 MM vaccines to children in areas with heavy deployment of both indoor residual spray and long-lasting insecticide treated nets. Simultaneous deployment of many interventions has a huge logistical and financial cost associated with it. The additional protection it offers should be commensurate to the implementation cost. Relevant studies conducted in Uganda were clinical trials evaluating the impacts of single interventions. No easily available study reviewed secondary data to compare the effectiveness of different combinations of malaria vector control measures in moderate to high transmission settings such as Gulu and Yumbe. This study measured the impact of compounds used in different brands of IRS and ITNS, different permutations of IRS+ITNS on malaria test positivity rates in high disease transmission settings of Yumbe district and Gulu city.

MATERIALS AND METHODS

Study settings, population and deployment of Malaria mosquito vector control interventions.

This study reviewed Malaria related demographic and clinical data from District Health Information Systems (DHIS2) of Gulu and Yumbe districts.

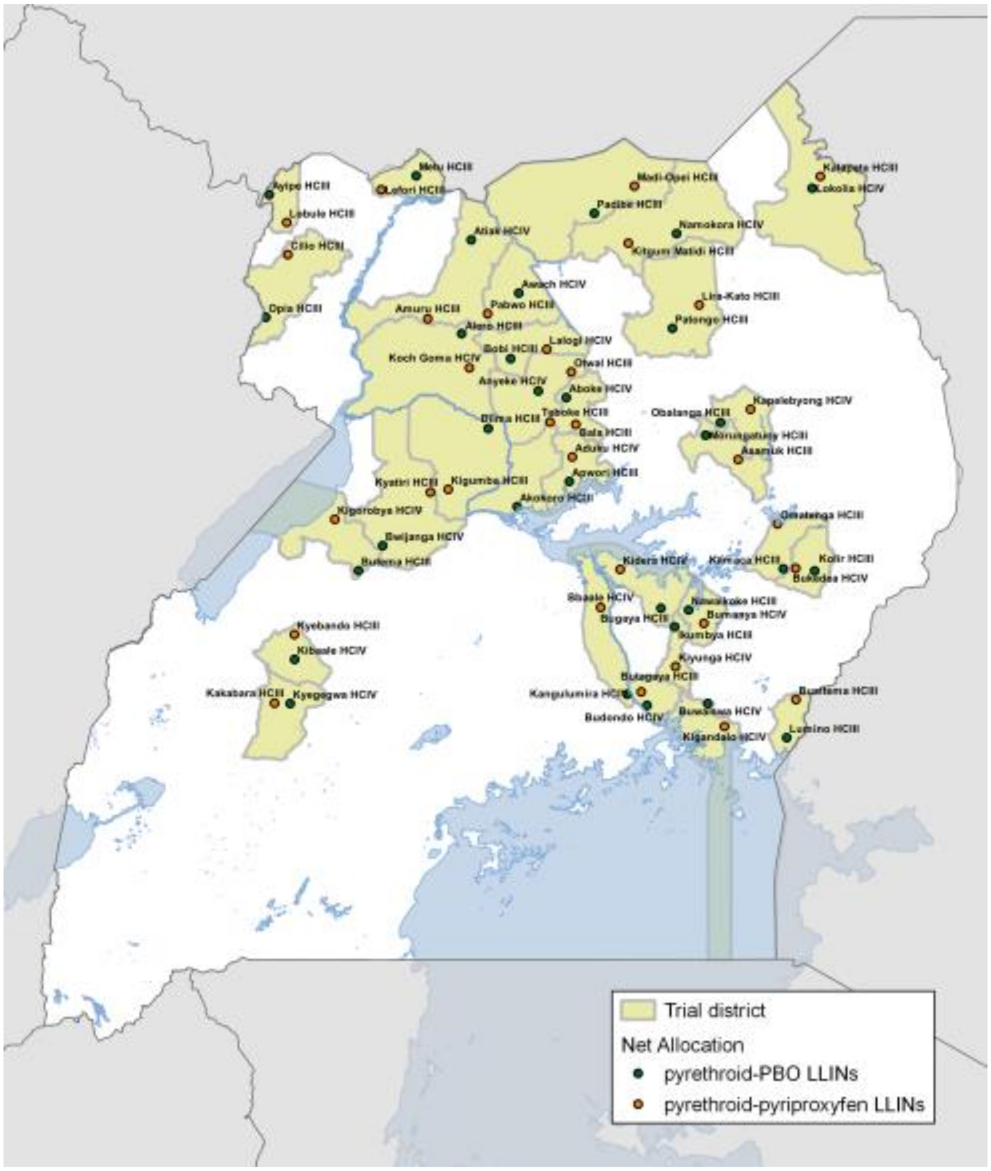

Fig 1: Map of 64 Malaria Reference Centers and their net allocations (Gulu and Yumbe districts inclusive). LLINs, long-lasting insecticidal nets; PBO, piperonyl butoxide. Map was adopted from [***https://doi.org/10.1136/bmjgh-2024-017106***](https://doi.org/10.1136/bmjgh-2024-017106)

Yumbe district (IRS + ITNS)

Yumbe district is located in the West Nile Region of Uganda (fig 1). It directly shares border with the Republic of South Sudan on the Northern part and the Democratic Republic of Congo to the west (fig 1). It is located approximately 600kms away from Kampala (The administrative and economic capital of Uganda). Malaria transmission is perennial in this area. Transmission peaks during rainy season (between April to October) and drops during dry season. The area has one of the highest entomological inoculation rates globally. Because of the complex population mixture (highest concentration of refugee camps), high transmission and strategic location, Government and partners have intensively invested in the deployment of different combination of malaria control interventions in the area; notably, Indoor Residual Spray (IRS) and mass scale distribution of Insecticide Treated Bed nets (ITNs).

Mass scale application of IRS, distribution of Bed Nets.

From January 2020 to December 2024, three rounds of IRS were sprayed and two rounds of ITNS were distributed in Yumbe district. In December 2020, there was mass scale distribution of ITNS in all sub-counties, including refugee camps (table 1). All the 18 sub-counties received standard Insecticide treated bed Nets (ITNs) with a brand name Yorkool. The Yorkool net is a G5 LN model chemically composed of Alpha-cypermethrin and chlorfenapyr. In December 2022, there was a district wide application of indoor residual spray (IRS) using Fludora Fusion. Fludora is a mixture of clothianidin (neonicotinoid) and deltamethrin (a pyrethroid). In December 2023, there was another mass scale distribution of ITNS; but the brands were changed to Royal Guard. Royal Guard is chemically composed of pyrethroid and pyriproxyfen. In December 2023, another cycle of mass IRS was repeated but using a different compound; Actellic 300 (table 1) . Actellic 300 is an organophosphate majorly composed of pirimiphos-methyl. In December 2024, Actellic 300 was again applied in the same area. However, this study did not measure the impact of the last round of IRS application. These interventions were deployed on top of the ITNS given to babies and mothers during immunization and antenatal visits. In addition, gestational mothers were equally given ITNS alongside Intermittent Preventive Therapy in pregnancy (IPTp).

Gulu District/City (ITNS only)

Gulu district/city is located in the Mid Northern part of Uganda (fig 1). The city is the main administrative and economic center of the region. Because of its strategic location between Kampala (about 350 Kms) and Juba (about 700 kms), it has become an economic hub for Uganda and South Sudan (fig 1). Acholi is the predominant indigenous ethnic group. Farming and trade are the main economic activity in the rural and city settings respectively. From 1990 to about 2010, the Acholi region went through turmoil from the armed conflicts by the Lord Resistance Army (LRA). Thousands were

displaced and put in internally displaced People's (IDPs) camps. The war reversed many gains in the health sector, especially malaria reduction and elimination achievements. Because of many swamps, low and flat lands and the nature of vegetations, the region has many fertile grounds for mosquito breeding. Just like Yumbe, the area has high perennial malaria transmission. It also has a similar rainfall pattern; with wet season stretching from April to October and dry spell after. Malaria burden in this region is one of the highest in Uganda and globally

Mass ~~scale~~ distribution of bed nets, IRS application in Gulu.

In 2006 , Gulu was among ten districts selected for the pilot roll out of IRS in Northern Uganda. The program went on consistently till 2014 when it was temporarily suspended due to non-satisfactory impact, logistical factors among others. From 2015 till date, Government of the republic of Uganda and partners embarked on mass scale distribution of Insecticide Treated Nets (ITNs) as the sole intervention measure. After every three years, there is a large-scale distribution of ITNs. This is on top of the ones given to mothers during antenatal visits and babies during immunization schedules. From 2020 till when this data was abstracted, two rounds of Insecticide Treated Nets (ITNs) were distributed in the communities. In December 2020, fifteen(15) sub-counties received ITNS (brand name:PermaNet). PermaNet is ~~chemically composed of~~ pyrethroid and Piperonyl- butoxide (table 1). In December 2023, another round of mass ITN~~S~~ distribution was implemented using PermaNet brands (table 1).

	2020/21	2022/2023	2023/24	2024/25
Yumbe district	Yorkool LLINS	Fludora Fusion (IRS)	Actellic 300(IRS) and Royal Guard ITNS	Actellic 300, and Royal Guard ITNS
Gulu district	PermaNet ITNS	No interventions	PermaNet ITNS	No intervention

Table 1: mass scale IRS applications, ITNS distributions in Yumbe and Gulu districts.

Data generation and abstraction from the District Health Information Management System for Yumbe and Gulu districts.

We briefly describe how DHIS 2 data was generated. When patients arrive at a Health facility, a clinical examination and clerkship is done. Following clinical assessment, patients are referred to the Laboratory for malaria blood tests, test conducted and results together with accompanying meta data entered into the Laboratory register. Routinely, health facilities organize outreaches to communities. Malaria tests using RDTs are usually conducted. Other community programs include integrated immunization and vaccination, mass malaria testing and treatment. Apart from

outpatient departments, malaria tests are also done in the maternity wards, inpatients departments, antenatal clinics, special clinics among others. Data generated from all the different points are summarized into the Health Information Management system (HIMS) and submitted to respective districts. Districts consolidate all information and submit to the Ministry of Health through District Health Information System 2 (DHIS 2). This is the data that we reviewed, analyzed and reported in this article.

Ethical approval/consideration.

The ~~overall~~ study was approved by the Makerere University School of Biomedical Sciences Research and Ethics Committee (SBS-REC) with approval number SBS-2023-362. Study was also approved by Uganda National Council of Science and Technology (UNCST) with approval number HS5306ES. To access data, permission was obtained from the Director General of Health Services, Ministry of Health. Access to data was granted on 11th February 2025 and data retrieval followed immediately (approval letter attached). There was no need to obtain consent from individual patients because the data was anonymized.

Statistical analysis: We summarized the demographic and clinical information of participants in a table using Microsoft word. Using a trend plot in STATA ver 17, we visually displayed a five (5) years trend in malaria test positivity rates for Yumbe and Gulu district respectively .A regression analysis was performed to quantitatively determine the relationship between test positivity rate and deployment of mass malaria control intervention. The Harris-Tzavalis (HT) test informed the decision to use the stationery rates to perform a regression analysis as opposed to converting it to first difference. Time was stratified into pre-intervention, intervention and post-interventions periods for the December 2020 ITN distribution phase, December 2022 IRS application in Yumbe and no intervention in Gulu, and December 2023 co-deployment of IRS+ITNS in Yumbe, ITNS distribution in Gulu city. Using Generalized Estimation Equation(GEE) population averaged model, we statistically determined the impact of different combinations of control interventions on TPR at different points. Because of low cluster, we used standard OLS regression with an interaction term to get the estimate . A parallel trends assumption was performed and parallel trend graphs plotted to address the same challenge of OLS underestimating the standard error in DiD that could potentially make the results look more significant than they actually are.

RESULTS:

Summary of demographic and clinical information

Averagely, Yumbe had an average Test Positivity Rate (TPR) of 57.4%) while that of Gulu was 42.2% (table 1). Household ITNs coverage was higher in Gulu (86%) compared to Yumbe (74.15%). Household IRS coverage in Yumbe was 74.15%. ~~Long~~

Lasting Insecticide Treated Nets were constituted 72.27% of total bed nets in Gulu (table 2). For the five years period, a total of 2,851,218 malaria tests were done in Yumbe while 2,017,655 malaria tests were done in Gulu. For Yumbe, number of cases were highest in children aged 29 days to 4 years (512,970 (31.3%) while for Gulu, cases were highest in adults (table 1). Malaria was more prevalent among gestational mothers in Gulu (3.07%) compared to Yumbe ((1.76%) (table 2).

**Characteristics/variable**	**Yumbe district**	**Gulu district**
Malaria control interventions	Indoor Residual Spraying (IRS), Insecticide Treated Nets (ITNs)	Insecticide Treated Nets (ITNs)
Intervention coverage in the communities	74.15% of households were sprayed with IRS,98.42% had at least one bed net	86% had at least one bed net, 72.7% being PBO nets
Total number of Malaria Tests done(Microscopy + RDTs)	2,851,218	2,017,655
Microscopy done	297,716	537,222
RDTs done	2,553,502	1,480,433
Malaria positivity by microscopy	158,603 (53.27%)	148,082(27.56%)
Malaria Positivity by RDTs	1,479,198(57.9%)	704,775(47.6%)
Malaria diagnosis by age category		
0 to 28 days	432 (0.026%)	780 (0.91%)
10 years to 19 years	423,133 (25.8%)	261,503 (30.6%)
20+ years	423,905 (25.8%)	276,425 (32.4%)
29 days to 4 years	512,970 (31.3%)	192,648 (22.6%)
5years to 9 years	329,064 (20.1%)	151,232 (17.7%)
Malaria in pregnancy		
10 to 19 years	9620(0.59%)	8373(0.98%)
+ 20 years	19287(1.17%)	17838(2.09%)
Mean Parasite prevalence		
Below 15 years	428.35 parasites/ul	359.69 parasites/ul
Above 15 years	128.43 parasites/ul	190.9 parasites/ul

Table 2: Demographic and clinical characteristics of patients from Yumbe district and Gulu City

A five years (January 2020 to December 2024) malaria monthly test positivity rates and trends with interventions in Gulu City and Yumbe district

From January 2020 to mid-2023, Yumbe district reported consistently high-test positivity rates compared to Gulu city (fig 1A). Both settings exhibited high seasonal fluctuations in test positivity rate and possibly malaria transmission rates. The

noticeable peaks and troughs in both Yumbe and Gulu correspond with high rainfall intensity in the areas. According to the data from the Uganda metrological center, both settings receive significantly higher rainfall intensity from the Month of May to December which do corresponds to the peak values in both settings. Following the distribution of Yorkool ITNS in Yumbe and PermaNet ITNS in Gulu, there was a dramatic decline in TPR in both settings (fig 2b). In July 2021, Yumbe saw a peak reaching near rates of 0.85 while Gulu remained relatively low during the same period (fig2A). Following the application of Fludora fusion IRS in Yumbe in December 2022 (Fig 2b), there was a significant drop in rates; similar magnitude of reduction within the same time point was observed in Gulu city though there was no mass scale deployment of malaria control intervention (Fig 2a & 2b). From around June 2023, the gaps between the two locations quickly narrowed and even became narrower throughout 2024 following mass distribution of PermaNets in Gulu city and deployment of a combination of Acetellic 300 IRS+Royal Guard ITNS in Yumbe district in December 2023 (fig 2a&2b).Summarily, Yumbe started with a much higher and volatile rates but by the end of the data collection period, both settings had declined and stabilized at lower rates

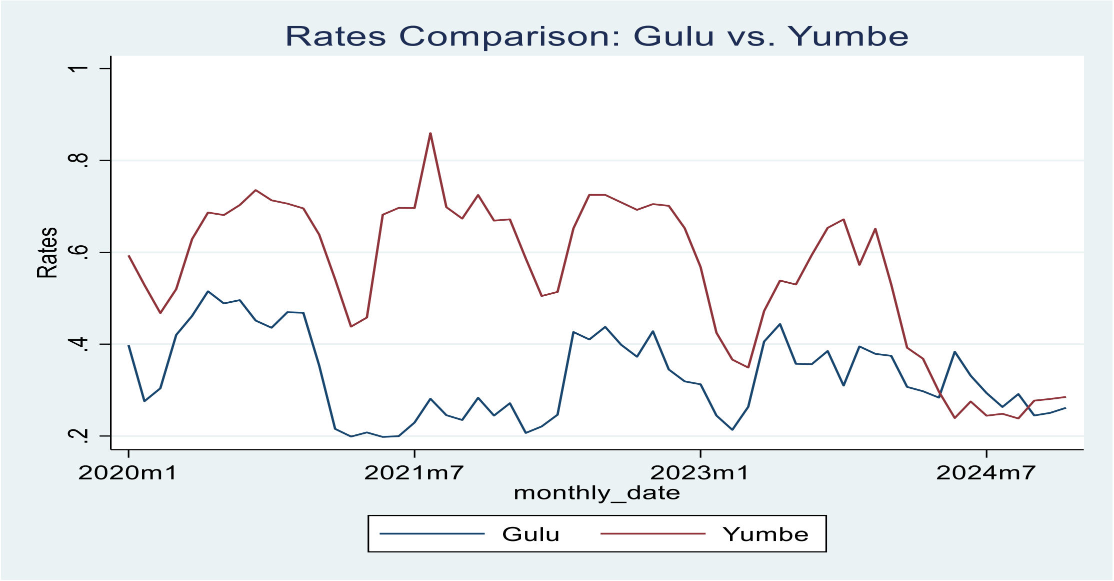

Fig 2A: A combined graphical display of a ~~five years~~ (January 2020 to December 2024) malaria monthly test positivity rates and trends in Gulu City and Yumbe district

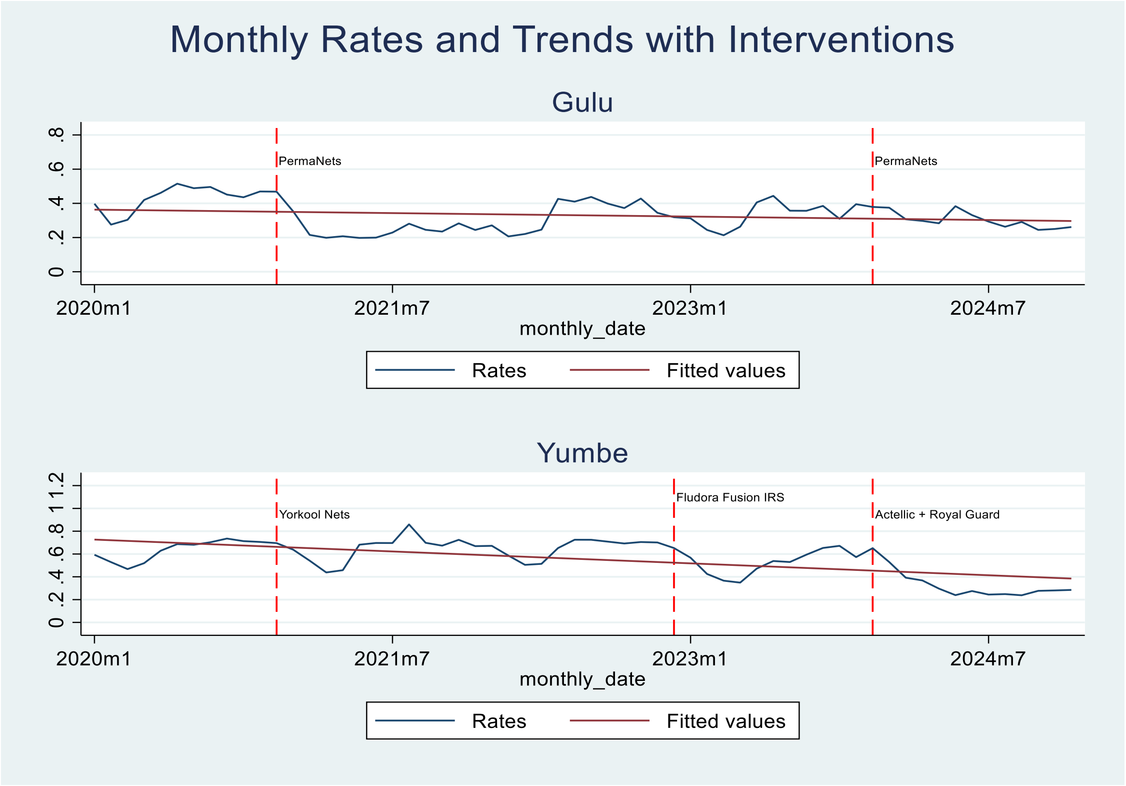

Fig 2A: A graphical display of a five years (January 2020 to December 2024) malaria monthly test positivity rates and trends with interventions in Gulu City and Yumbe district

We proceeded with the panel regression analysis using stationery rates because the P value from the Harris-Tzavalis (HT) test was 0.037 (significant) thus no need to convert our rates to first order before proceeding with the regression analysis. The dependent variable was test positivity rates while the independent variables were time and locality (table 3, fig 3).We report a major difference in malaria test positivity rate between Gulu and Yumbe. There was downward trend in both settings though only one was statistically significant. For Gulu city, there was a weak non-significant co-efficient of -0.011; p=0.093(table 3) with a very slow monthly unit dropping rate of only 0.001 making it hard to disapprove the argument that the drop could be by chance than attributing it to the impact of interventions (fig 3). We also report that only 4.8% of Gulu's rate is explained by variation in time while the other 95% could be due to volatility in months and other external factors such as variability in rainfall intensity. For Yumbe district, we report a significant strong coefficient (-0.0058) with a much faster reduction rate of 0.006 units per month; p<0.00 (about 5 times faster than Gulu City) (fig 3, table 3). About 38.5% of reduction rates can be explained by variation in time or deployment of control interventions. We conclude that the visual fitted values line in Yumbe represent a real reduction while for Gulu is mostly a mathematical average of noisy data (fig 3).

Location	coefficient	R-square	t-value	P-value	95% CI
Gulu city	-0.001126	0.0478	-1.71	0.093	-0.0024411 to 0.000194
Yumbe district	-0.0057918	0.3846	-6.02	<0.00	-0.0077175 to -0.0038661

Table 3: regression analysis for a five year malaria monthly test positivity rates and trends with interventions in Gulu City and Yumbe district

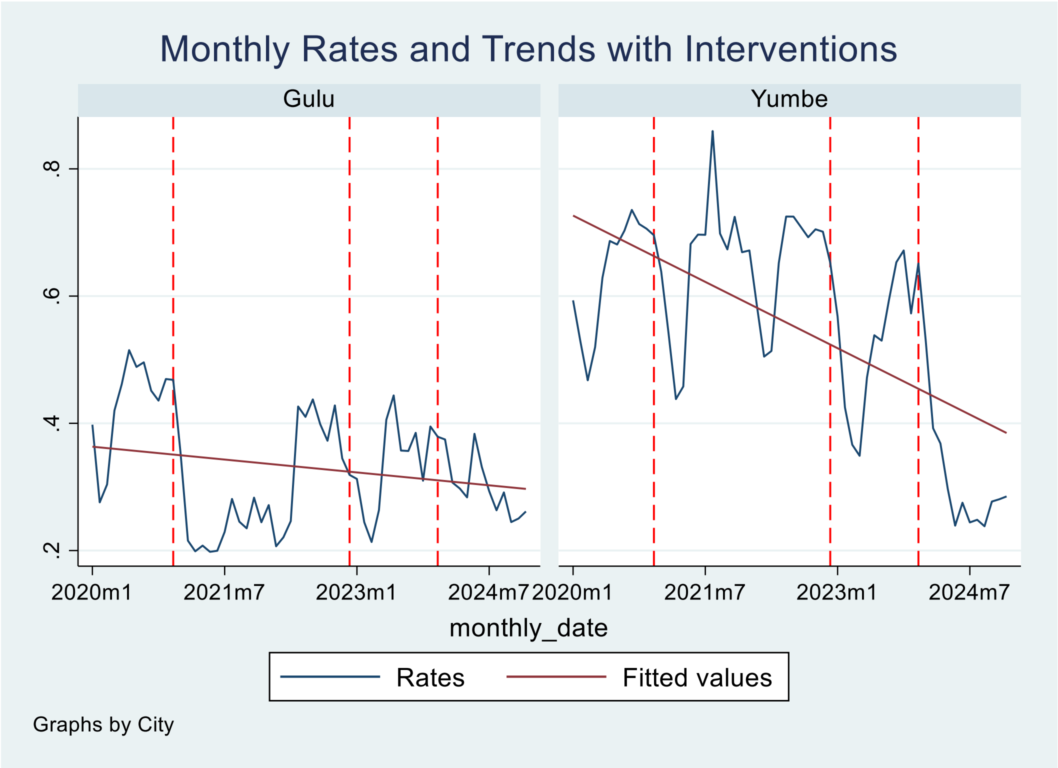

Fig 3: graph comparing monthly rates and trends with interventions between Gulu and Yumbe districts

Effectiveness of Yorkool ITNS (Yumbe district) versus PermaNet ITNS (Gulu city) distributed in December 2020 in reducing malaria test positivity rate.

In December 2020, Ministry of Health distributed a new brand of ITNS (Yorkool) in Yumbe district while maintaining PermaNets ITNS in Gulu City. To statistically determine which brand was more effective, we disaggregated time into three strata and compared positivity rates at pre-intervention (January to November 2020), intervention (December 2020) and post-intervention (January to November 2021). A regression analysis was performed using Generalized Estimation Equation(GEE) population averaged model. Yumbe with a new brand was termed “intervention population” while Gulu with the old brand was termed “control population”.

For the pre-Intervention Trends _t (Time Trend), our coefficient (0.0129) shows a statistically significant increase in the rate for the Gulu city settings during the ITN pre-distribution period (table 4). The coefficient (0.1667) of the group difference (_z) indicates that at the start of the study, the intervention setting (Yumbe) had a significantly higher rate than the Gulu setting. Following PermaNet ITN distribution in Gulu and Yorkool ITNS in Yumbe, there was immediate Impact in January 2021 (_x2021m1;Level Change - Control) with significant immediate drop in the rate for the PermaNet ITN population (Gulu). The coefficient (0.0532) for the Yumbe vs.Gulu (_z_x2021m1 Level Change) is not statistically significant suggesting that the dramatic reduction at the start of the intervention was not significantly different between the Yumbe(Yorkool) and Gulu(PermaNet) population (table 4). We also determined change in trends (Slopes;_x_t2021m1) but reported that control group's trend changed by , but it was not statistically significant. We also statistically estimated difference in trend Change (_z_x_t2021m1) and found the coefficient (0.0111) to be statistically insignificant. Conclusively, we failed to find any evidence to support the hypothesis that distribution of new brand of ITNS (Yorkool) in Yumbe district led to a higher reduction in malaria test positivity rate over time compared to Gulu that maintained PermaNet ITNS (table 4).

Rate	Coefficient	Std. err.	Z	P>|z|	[95% conf. interval]	
_t	.0128522	.0054069	2.38	0.017	.0022549	.0234495
_z	.1667266	.0460477	3.62	0.000	.0764748	.2569784
_z_t	.0071989	.0076465	0.94	0.346	-.007788	.0221857
_x2021m1	-.2770566	.0530682	-5.22	0.000	-.3810684	-.1730449
_x_t2021m1	-.0123957	.0076465	-1.62	0.105	-.0273825	.0025911
_z_x2021m1	.0531764	.0750498	0.71	0.479	-.0939185	.2002712
_z_x_t2021m1	.0111113	.0108137	1.03	0.304	-.0100833	.0323058
_cons	.3612873	.0325606	11.10	0.000	.2974696	.4251049

Table 4: *A regression analysis using Generalized Estimation Equation(GEE) population average model*

We visually compare trends in test positivity rate before and after ITN distribution in December 2020 using an interrupted time series plot (fig 4).The coefficient for the baseline pre-intervention trend;_t (0.0128) suggests that the test positivity rate in the control group (Gulu City) was significantly increasing by approximately 0.013 units per month. We also found the coefficient (0.1667) of the difference in both settings (_z) at the very beginning of the study (month 1) to be relating with a significantly higher rate in Yumbe with Yorkool ITNS compared to Gulu with PermaNet. The sudden negative coefficient(-0.2770) during the ITNS distribution phase in both sites (_x2021m1) relates with a highly significant immediate drop in the TPR for the Gulu population in January 2022(fig 4) . This is suggestive of the positive impact of the PermaNet ITNS distributed .The coefficient (-0.0123) for the change in trend (_x_t2021m1) representing the change in the slope for the Gulu population after January 2021 is not significant at the standard 5% level but shows a marginal trend toward the TPR leveling off (fig 4). We also calculated the intervention effect (Difference-in-Differences). The level change difference (_z_x2021m1) of 0.0531 shows that the dramatic reduction in TPR (January 2021) immediately after ITNS distributions was not significantly different between Yumbe and Gulu(fig 4). The trend change difference (_z_x_t2021m1) of 0.0111 suggests that the distribution of the new brand of ITNS (Yorkool) did not significantly change the long-term trend of Yumbe relative to Gulu(fig4). We therefore conclude that that the introduction of Yorkool ITNS did not have a unique or different impact on malaria burden in Yumbe District compared to Gulu City that maintained PermaNet ITNS.

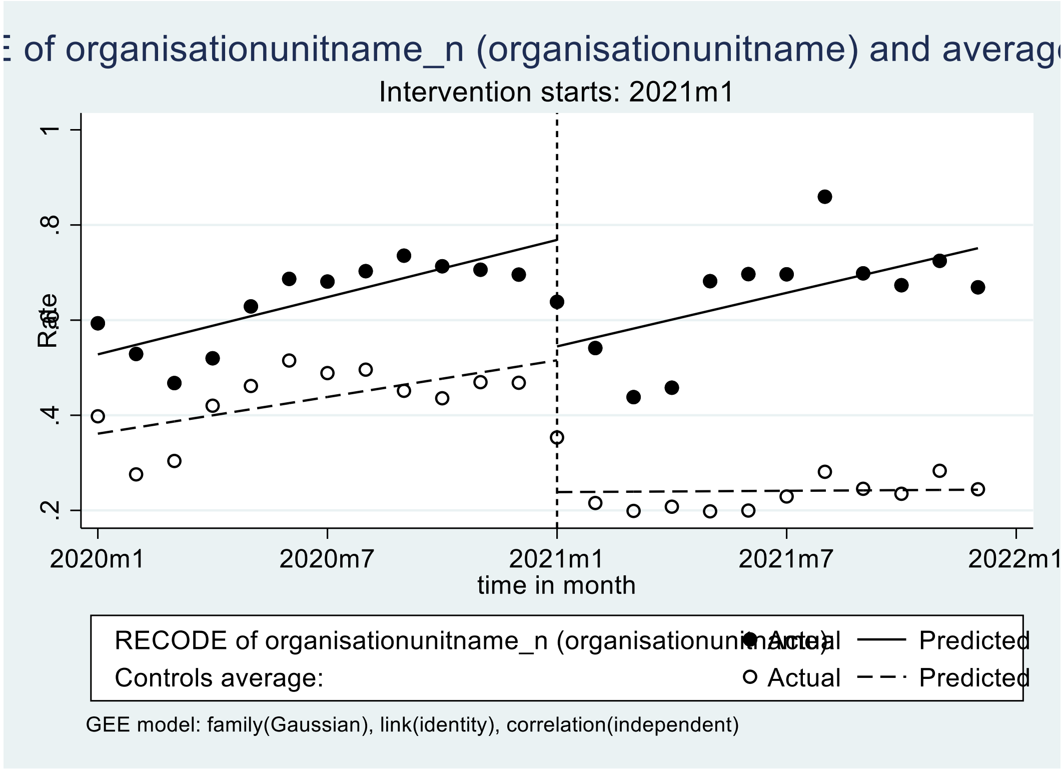

Fig 4: *An interrupted time series plot to compare trends in test positivity rate before and after ITN distribution in December 2020*

malaria monthly test positivity rates and trends with deployment of Fludora fusion IRS in Yumbe (December 2022) and no intervention in Gulu

The time points were again split into three; pre-intervention(January to November 2022), intervention (December 2022) and post intervention (January 2023 to November 2023). We performed an interaction regression to statistically determine the impact of fludora at different time points. We also plotted graphs from parrel trend assumptions of the difference in Difference model. The coefficient (-0.0046682) for the difference in slopes (City_n#c.monthly_date) confirms that Yumbe's rate fell by an additional 0.0047 units per month compared to Gulu; P-value <0.000 confirming high significance (table 5, fig 5). There is strong statistical evidence that Yumbe was declining significantly faster than Gulu and the two cities were not following the same trend. The P value (0.175) for the "base" city (Gulu) (row monthly_date) is statistically insignificant. Effectively, Gulu's rate is flat or stable, while Yumbe's is crashing. The coefficient 3.724 for the starting gap (City_n | Yumbe) shows that Yumbe started with significantly higher rates than Gulu. We therefore conclude that while both cities show a visual downward trend, an interaction model confirms that the decline in Yumbe is significantly steeper than in Gulu

Rates	Coefficient	Std. err.	t	P>|t|	[95% Conf.Interval]	
City_n (Yumbe)	3.724412	.8738816	4.26	0.000	1.99358	5.455245
monthly_date	-.0011236	.0008242	-1.36	0.175	-.0027561	.0005089
City_n#monthly_d	-.0046682	.0011656	-4.00	0.000	-.0069769	-.0023595
_cons (Constant)	1.172229	.6179276	1.90	0.060	-.0516546	2.396112

***Table 5**: an interaction regression on malaria monthly test positivity rates and trends with deployment of Fludora fusion IRS in Yumbe (December 2022) and no intervention in Gulu*

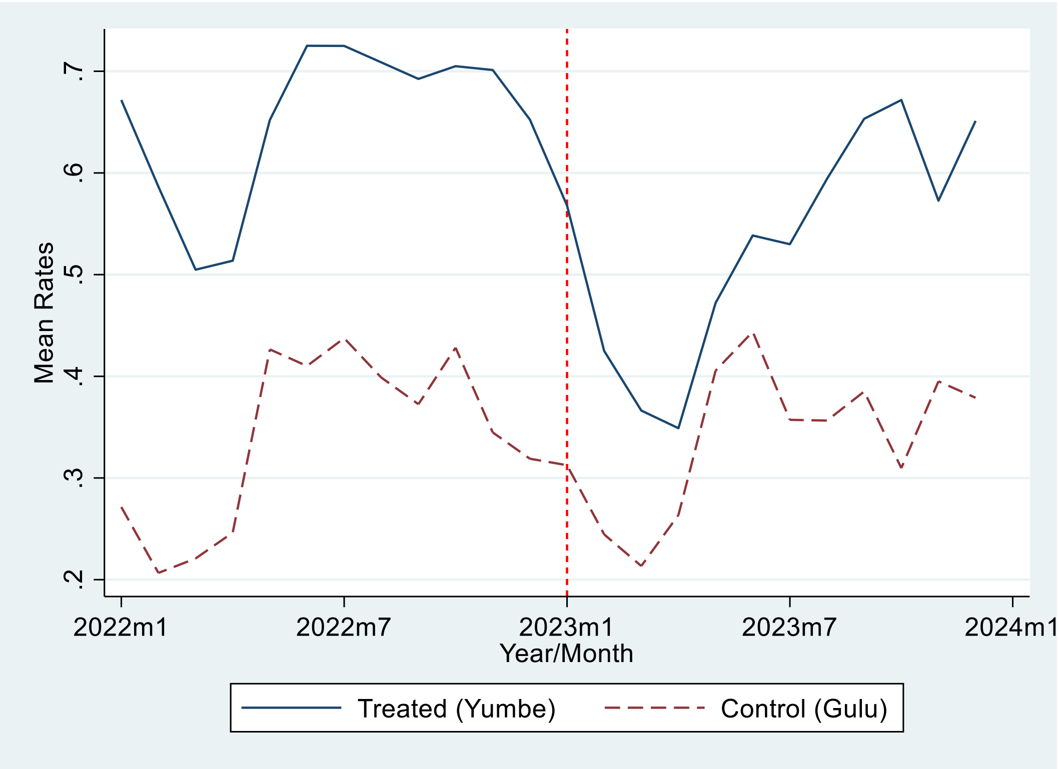

Fig 5: Plot on malaria monthly test positivity rates and trends with deployment of Fludora fusion IRS in Yumbe (December 2022) and no intervention in Gulu

From the standard diagnostic tool graph, we evaluated the parallel-trends assumption for Difference-in-Differences (DiD) models. The observed means (left plot) shows the raw average rates for the intervention(Yumbe) and control (group)groups over time. For the pre-treatment (Left of the red line): Our DiD model is valid because the blue (Control) and red (intervention) lines follow a similar, parallel path & fluctuations; (a typical example is the peaks in mid-2022), suggesting they were influenced by similar external factors (possibly rainfall) before the application of fludora fusion IRS began. For the post-IRS application (right of the red line), both groups drop significantly, but the red Treatment line (Yumbe) stays consistently lower than the blue Control line compared to their pre-treatment relationship. This visual gap represents the negative

ATET of -0.106. The linear-trends model (Right Plot) is a more formal diagnostic that augments the DiD model with interaction terms to specifically test if the trends are linear and parallel. Summarily, the graphs suggest that the DiD model is largely valid with parallel Trends plots showing that the two districts moved together before the treatment. On impact, there is a distinct, sustained downward shift in the IRS's rates relative to the control group after the red line, confirming that fludora fusion significantly reduced malaria burden in Yumbe district.

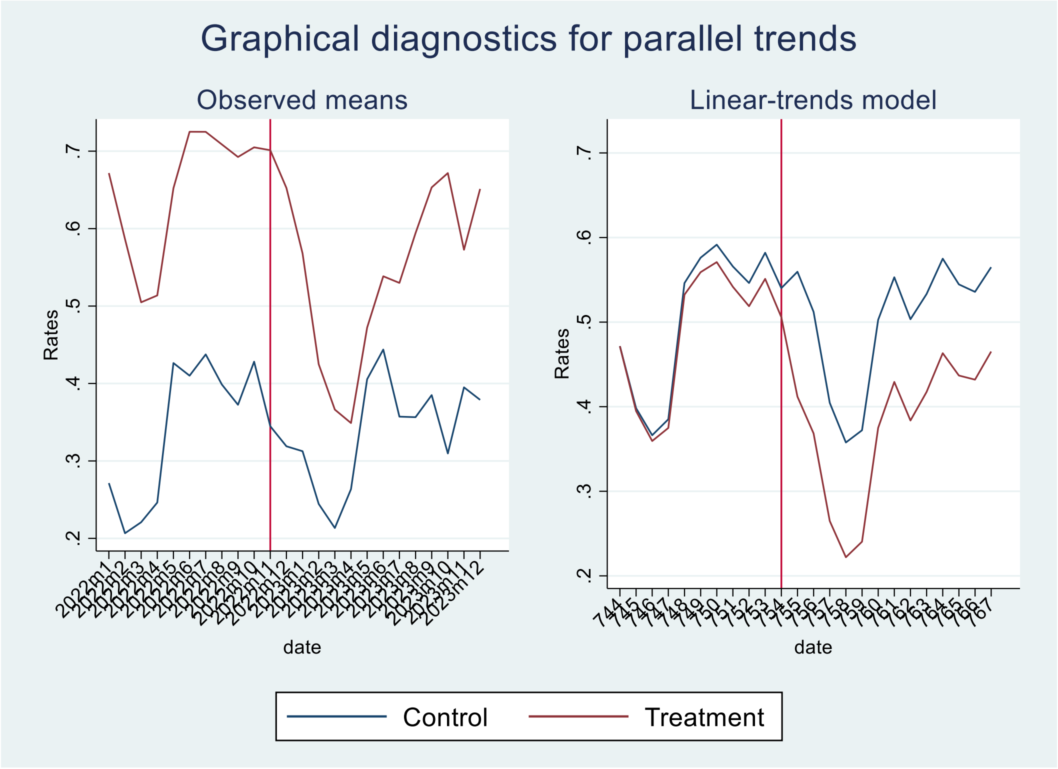

Fig 6: The standard diagnostic tool graph to evaluate the parallel-trends assumption for Difference-in-Differences (DiD) models

malaria monthly test positivity rates and trends with simultaneous deployment of Acetellic 300 IRS + Royal guard ITNS in Yumbe (December 2023) and a repeat of PermaNet distribution (December 2023) in Gulu City.

We performed a regression analysis to identify the Difference-in-Differences (DiD) effect (table 6, fig 7). Because of having only two groups (clusters), we used a standard OLS regression (reg) with an interaction term to get the estimate. The DiD Coefficient (-0.1813;the interaction term 1.is_treated_group#1.post_period) indicates that simultaneous deployment of Acetellic 300 IRS and Royal Guard ITNS was associated with a 0.1813 unit decrease in malaria test positivity rate in Yumbe district relative to Gulu City, after controlling for baseline differences and time trends. This decrease was

statistically significant because the P-value was 0.003 at 1% level (table 6, fig 7). The coefficient -0.0436 (1.is_treated_group) shows that before the intervention, Yumbe had rates 0.0436 units lower than Gulu (not statistically significant,).The coefficient 0.1044 (1.post_period) shows that in the Post period, TPR in the Gulu population increased by 0.1044 units compared to its own baseline/pre-intervention rate of 0.3835 (_cons) (table 6, fig 7).

Rates	Coefficient	Std. err.	t	P>|t|	[95% conf. interval]	
1.is_treated_group	-.0436357	.0401109	-1.09	0.283	-.124527	.0372556
1.post_period	.					
	1044281	.0401109	2.60	0.013	.0235368	.1853194
is_treated_group#post_period						
1 1						
	-.1813122	.0573664	-3.16	0.003	-.2970025	-.0656219
_cons						
	.3835375	.0283627	13.52	0.000	.3263387	.4407363

Table 6: Regression analysis of malaria monthly test positivity rates and trends with simultaneous deployment of Acetellic 300 IRS + Royal guard ITNS in Yumbe (December 2023) and a repeat of PermaNet distribution (December 2023) in Gulu City.

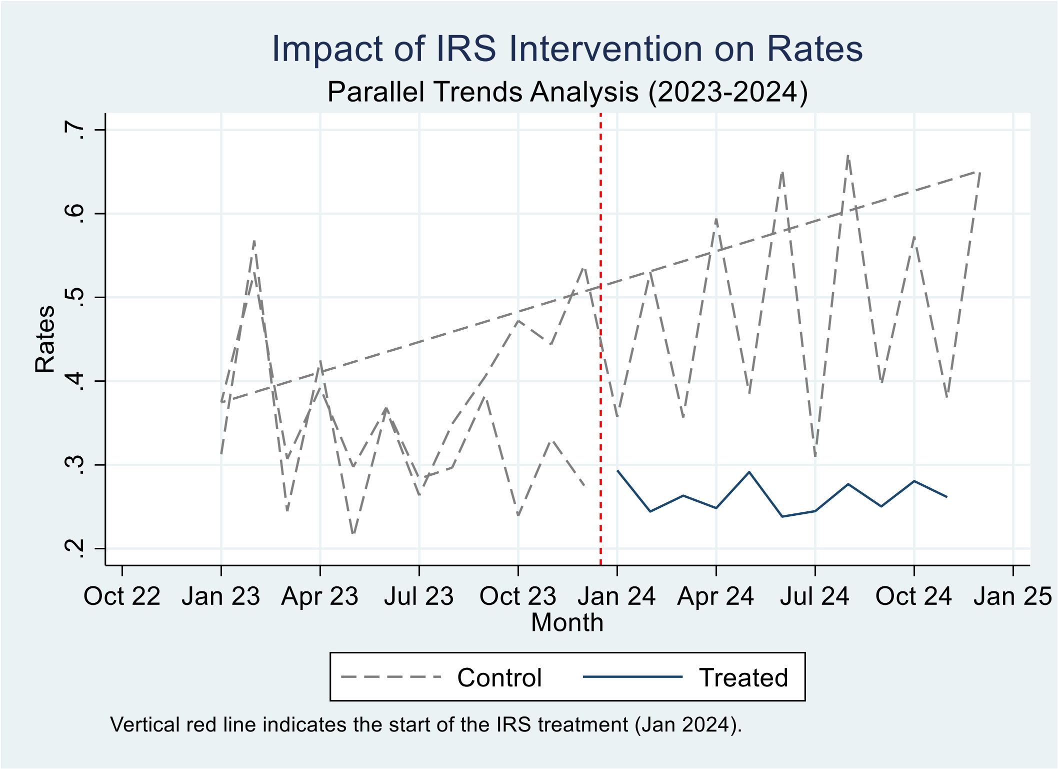

Fig 7: Difference in Difference plot of malaria monthly test positivity rates and trends with simultaneous deployment of Acetellic 300 IRS + Royal guard ITNS in Yumbe (December 2023) and a repeat of PermaNet distribution (December 2023) in Gulu City.

We also performed a parallel trends assumption and plotted parallel trend graphs to address the challenge of OLS (because of few clusters) underestimating the standard error in DiD which makes the results look more significant than they actually are (fig 8). We found the Average Treatment Effect on the Treated (ATET) to be -0.1664675 suggesting that after adjusting for panel and time effects, the intervention (Acetellic 300 IRS+Royal guard ITNS) is associated with a 16.6 percentage point reduction in malaria test positivity rates in Yumbe district compared Gulu City. From the plot, we note that between January to July 2023 (pre-intervention months), the two lines (Gulu City and Yumbe District) tracked each other remarkably well, exhibiting the same sawtooth seasonal pattern, moving up and down in unison(fig 8). This is strong visual evidence that the Parallel trends assumption holds for the first half of the pre-treatment year. However, we noted that from August 2023 (three months before deployment of IRS and ITNS), Gulu City's rates started an upward climb while Yumbe District's rates began a steady decline. After the dashed line (post-intervention), the divergence becomes even more pronounced(fig 8). Gulu City (blue) remains extremely volatile with very high peaks, while Yumbe District (red) stabilizes at a much lower rate suggesting a significant negative effect (ATET of -0.166). This graph supports the finding that Yumbe District with ITN+IRS maintained lower and more stable malaria rates compared to the rising volatility in Gulu City with only ITNS(fig 8).

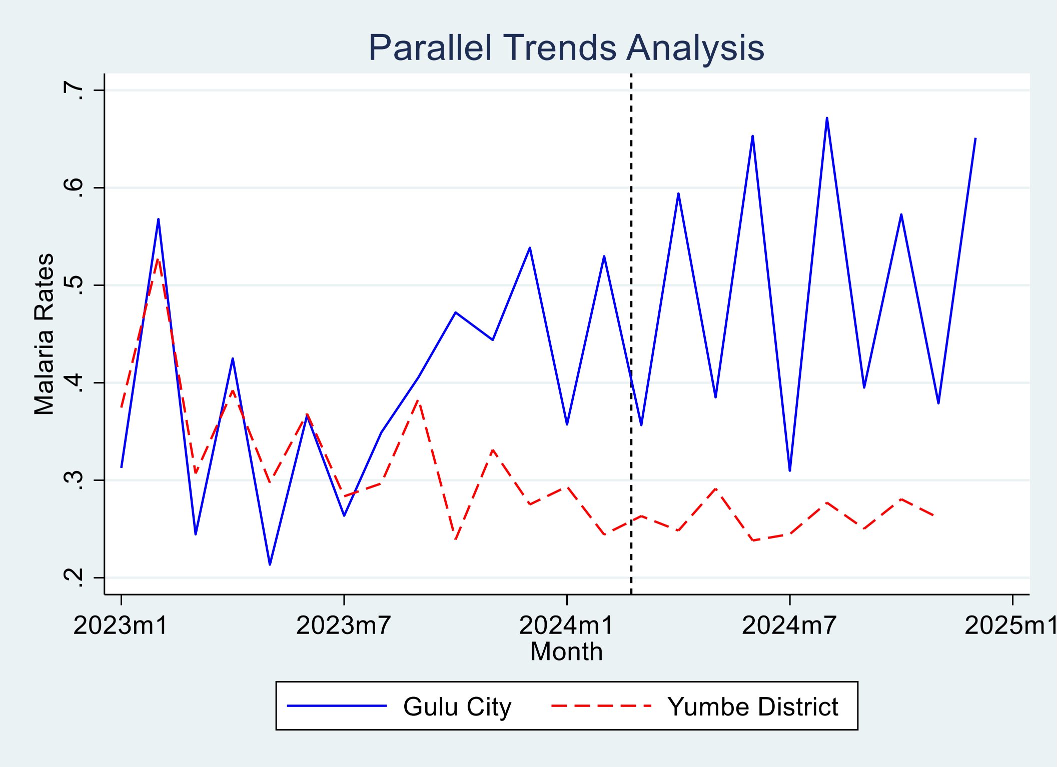

Fig 8: The standard diagnostic tool graph to evaluate the parallel-trends assumption for Difference-in-Differences (DiD) models

Discussion

This study measured the impact of compounds used in different brands of IRS and ITNS, different permutations of IRS+ITNS on malaria test positivity rates in high disease transmission settings of Yumbe district and Gulu city.

For the five-year malaria trend analysis, test positivity rates in Yumbe district dropped five times faster than that of Gulu City (p<0.00) with 38.5% of reduction rates in Yumbe attributable to deployment of different combinations of control interventions. Our findings agrees with that of a four years retrospective study done in Eritrea on the impact of combinations of control interventions on malaria test positivity rates[18]. They reported that malaria morbidity was strongly correlated to the number of ITNS distributed and the amount of DDT and malathion used in IRS. The current study and the Eritrean study both reviewed secondary data though we only ~~did~~ ~~compare~~ the impact of the brands/compounds while the Eritrean study quantified and compared the impact of the active compounds in both IRS and ITNS[18]. Finding of the current study does not agree with that of a cross-sectional study conducted in selected urban and rural communities

in Benin[19]. The Benin study reported that for urban areas, sleeping under ITNs does not confer significant protection against malaria and uncomplicated malaria compared to no intervention at all. They further reported that in rural areas, the use of ITNs alone, IRS alone or a combination of the two did not provide additional protection compared to no intervention at all[19]. The Benin study used a cross-sectional study design while the current study reviewed secondary data retrospectively. The commonality between the two studies is the aspect of measuring the impact of ITN or a combination of IRS+ITN on malaria case reduction by rural or urban settings. For the current study, the comparison was between combination of IRS+ITN versus ITN alone while the Benin study compared ITN or IRS versus no intervention

This study also reported that introduction of new brand of ITNS (Yorkool) in December 2020 did not significantly change the long-term trend in TPR of Yumbe district relative to Gulu city that maintained distribution of PermaNets ITN for the same time point (The trend change difference of 0.0111). Our findings disagree from a WHO report which found Clinical trials and pilot studies on dual-insecticide nets to improve malaria control by 20–50% compared with standard pyrethroid-only nets. Our findings further disagrees with the additional WHO report that clinical trials in the United Republic of Tanzania and Benin demonstrated that the pyrethroid-chlorfenapyr nets significantly reduced malaria infections in children between the ages of 6 months and 10 years. Our findings does not fully agree with that of *Epstein A et al 2025* [5] that reported a higher reduction in malaria incidence from 827 cases per 1000 person years in the pre-distribution phase of newer generation nets to 538 cases per 1000 person years in the post distribution period. The Epstein A et al was a prospective study conducted in 164 sites across Uganda with relatively fewer sample size [5] while the current study reviewed secondary data involving over 5 million individuals giving it a higher statistical power. The Epstein study measured a one-time impact at 164 sites while the current study reviewed a five year data on laboratory confirmed malaria cases collected from over 100 health centers located within Yumbe and Gulu districts. Findings of the current study agrees with that of a study conducted in Burkina Faso and provided evidence supporting the introduction of both Interceptor G2 nets and PBO nets, which were distributed in Burkina Faso in 2019 as part of a mass campaign[20].

Following the application of Fludora fusion IRS in Yumbe district in December 2022 and no mass scale deployment of any control intervention in Gulu city for the same time point, the coefficient of -0.004668 for the difference in slopes confirms that Yumbe's rate significantly fell by an additional 0.0047 units per month compared to Gulu; P-value <0.000. Our findings agree with that of a systematic review and meta-analysis by *Zhou et al 2022* [21] on effectiveness of indoor residual spraying for malaria control. It reported that IRS was associated with lower rates of malaria infection (*OR*= 0.35, 95% *CI*: 0.27-0.44) and also that significantly higher effectiveness was observed in IRS

coverage ≥ 80% than in IRS coverage < 80%. The study also reported that Pyrethroids was identified to show the greatest performance in malaria control.[21]. The systematic review and meta-analysis by *Zhou et al 2022* had a fewer malaria related outcome measures while the current study was a review of secondary data with multiple malaria related outcomes. Findings of the current review agrees with that of a study on the impact of indoor residual spraying with pirimiphos-methyl (Actellic 300CS) on entomological indicators of transmission and malaria case burden in Migori County, western Kenya[22]. They concluded that a single application of IRS with Actellic 300CS in Migori County provided ten months' protection and resulted in the near elimination of the primary malaria vector *An. funestus* and a corresponding reduction of malaria case count among out-patients[22]. The Kenyan study was prospective longitudinal one with entomological outcomes while the current study was the review of the secondary data with test positivity rates as the major outcome.

We found the Average Treatment Effect on the Treated (ATET) to be -0.1664675 suggesting that after adjusting for panel and time effects, the intervention (Acetellic 300 IRS+Royal guard ITNS) is associated with a 16.6 percentage point reduction in malaria test positivity rates in Yumbe district compared to Gulu City. Our findings agree with that of *Echodu.R. et al* on the Impact of population based indoor residual spraying with and without mass drug administration with dihydroartemisinin-piperaquine on malaria prevalence in a high transmission setting[14]. They reported that implementation of IRS combined with ITNs was associated with 97.6% decrease in malaria incidence and 96% decrease in parasite prevalence, both measured after several rounds of IRS. However, when chemoprophylaxis is added, there was an even more drastic decrease in malaria incidence of up to 99.5%[14]. Our findings also agree with that of a Cochrane review by *Joseph Pryce et al 2022*[23]. They reported that in communities using ITNS, the addition of IRS with non-pyrethroid like insecticide was associated with reduced malaria prevalence but noted that the findings lacked heterogeneity. The Pyrce study measured the effects of combination of IRS +non-pyrethroid nets on malaria case reduction at only one-time point while the current study reviewed a five(5) years secondary data on the effects of combinations of different interventions on malaria test positivity in areas with moderate to high malaria transmission. This significantly improves the reliability findings from the current study to make it more relevant and reliable.

Conclusion: In areas of high malaria transmission, deployment of either ITNs alone, IRS alone or in combination can be an effective tool against malaria mosquito vector control .However, a more sustained reduction can only be achieved through simultaneous deployment of IRS+ITNS

Study limitation: The DHIS2 data reviewed, analyzed and used in this article was not standardized for research and could possibly lack completeness. Data on weather patterns, pyrethroid resistance, entomological inoculation rates and other environmental

factors were not easily available and so we did not adjust for all of them in the analysis. This could somehow affect some findings and results. However, the two settings have very similar rainfall patterns, malaria transmission intensity, similar population dynamics thus controlling for the effects of some of these confounders. Some Laboratory tests were conducted by non-WHO qualified staff raising a question of reliability. This was addressed by using RDT as opposed to microscopy results. We were unable to adjust for the effects of ITNS and IPT given to pregnant mothers and babies, but both settings received them thus neutralizing any possible impacts.

Funding: No agency, donor, or institution funded this study. It is part of a PhD project. Attempts are being made to secure one for the remaining objectives. Uganda Virus Research Institute and Makerere University offered in kind support.

Availability of Data: All relevant data will be made available. Data access links will be shared with the Journal and scientific editors.

Declarations

Consent to Publication: All authors have consented to ownership of the content of the manuscript and agreed that it can be published in this journal.

Conflict of Interest : All authors have declared no conflict of interest

Acknowledgment:

Uganda Virus Research Institute under the great leadership of Prof Pontiano Kaleebu, thank you for the opportunity to work with and learn from you. Dr. Jonathan Kayondo and the entire team at the department of entomology & parasitology-UVRI, I do appreciate your mentorship and support in genomics and bioinformatics. Let me extend my gratitude to Dr .Isaac Ssewanyana for the great mentorship in malaria immunology. Prof Kamya and the entire IDRC team, thank you for all kinds of support. Finally, all PhD students and peers at Makerere University, your morale and intellectual support is immeasurable .

Author contribution :EJO: Ideation & conceptualization, retrieving data from DHIS 2, drafting manuscript, identification of the journal & manuscript submission. SA: Reviewed the manuscript, adjusted the accuracy of the clinical data, wrote the abstract. BM: wrote introduction, improved information on methods section. AD : provided statistical software, conducted statistical analysis, reviewed section on results. SJO : Reviewed the manuscript. ML : Reviewed the manuscript. JL: provided logistics support, reviewed the manuscript. JK: provided office space, reviewed the analysis plan. MO: Offered approval to access Ministry of Health data, reviewed the analysis plan, reviewed manuscript.

Author Information: EJO is a PhD student at the Department of Immunology & Molecular Biology, Makerere University, a research fellow at Uganda Virus Research Institute. SA is a gynecologist at Lacor Hospital, a lecturer at the faculty of Medicine, Gulu University. BM is a Biomedical laboratory scientist, chief lab Technologist at Muni University . AD is a PhD student at the department of Biostatistics & Epidemiology, Makerere University, a lecturer at Muni University. SJO is an immunologist, a senior lecturer at the Department of Immunology & molecular Biology, Makerere University. ML is a post-Doctoral Research fellow at the entomology and parasitology department-UVRI. JK is the Head of Department Entomology and Parasitology department-UVRI, Principal Investigator for target Malaria. JL is a professor of Arbovirology at Uganda Virus Research Institute. MO is a pharmacologist, senior lecturer at the department of pharmacology and therapeutics, Makerere University.

Consent to Publications: All authors have consented to publishing this manuscript. All authors have declared no conflict of interest.

Ethical approval/consideration :The overall study was approved by the Makerere University School of Biomedical Sciences Research and Ethics Committee (SBS-REC) with approval number SBS-2023-362. Study was also approved by Uganda National Council of Science and Technology (UNCST) with approval number HS5306ES. To access data, permission was obtained from the Director General of Health Services, Ministry of Health. There was no need to obtain consent from individual patients because the data was anonymized

Reference

1. Oxborough RM, Chilito KCF, Tokponnon F, Messenger LA. Malaria vector control in sub-Saharan Africa: complex trade-offs to combat the growing threat of insecticide resistance. The Lancet Planetary Health. 2024;8(10):e804-e12.
2. Organization WH. World malaria report 2023: World Health Organization; 2023.
3. Egbujor CE, Innocent DC, Chukwuocha UM, Ekeleme UG, Ekenze JC, Dozie UW, et al. The effectiveness of interventions for improving the uptake of Intermittent Preventive Treatment (IPT) for malaria control among pregnant women in sub-saharan Africa: A systematic literature review. Archives of Preventive Medicine. 2023;8(1):008-19.
4. Yang G-g, Kim D, Pham A, Paul CJ. A meta-regression analysis of the effectiveness of mosquito nets for malaria control: the value of long-lasting insecticide nets. International journal of environmental research and public health. 2018;15(3):546.
5. Epstein A, Gonahasa S, Namuganga JF, Nassali MJ, Maiteki-Sebuguzi C, Nabende I, et al. Evaluating the impact of two next-generation long-lasting insecticidal

nets on malaria incidence in Uganda: an interrupted time-series analysis using routine health facility data. BMJ Global Health. 2025;10(3).
6. Ng'ang'a PN, Aduogo P, Mutero CM. Long lasting insecticidal mosquito nets (ITNs) ownership, use and coverage following mass distribution campaign in Lake Victoria basin, Western Kenya. BMC Public Health. 2021;21(1):1046.
7. Lengeler C. Insecticide-treated nets for malaria control: real gains. SciELO Public Health; 2004. p. 84-.
8. Aguma HB, Rukaari M, Nakamatte R, Achii P, Miti JT, Muhumuza S, et al. Mass distribution campaign of long-lasting insecticidal nets (ITNs) during the COVID-19 pandemic in Uganda: lessons learned. Malaria Journal. 2023;22(1):310.
9. Tukei BB, Beke A, Lamadrid-Figueroa H. Assessing the effect of indoor residual spraying (IRS) on malaria morbidity in Northern Uganda: a before and after study. Malaria journal. 2017;16:1-9.
10. Namuganga JF, Epstein A, Nankabirwa JI, Mpimbaza A, Kiggundu M, Sserwanga A, et al. The impact of stopping and starting indoor residual spraying on malaria burden in Uganda. Nature communications. 2021;12(1):2635.
11. Kamya MR, Nankabirwa JI, Arinaitwe E, Rek J, Zedi M, Maiteki-Sebuguzi C, et al. Dramatic resurgence of malaria after 7 years of intensive vector control interventions in Eastern Uganda. PLOS Global Public Health. 2024;4(8):e0003254.
12. Okumu FO, Moore SJ. Combining indoor residual spraying and insecticide-treated nets for malaria control in Africa: a review of possible outcomes and an outline of suggestions for the future. Malaria journal. 2011;10:1-13.
13. Oxborough RM, Chilito KLF, Tokponnon F, Messenger LA. Malaria vector control in sub-Saharan Africa: complex trade-offs to combat the growing threat of insecticide resistance. The Lancet Planetary Health. 2024;8(10):e804-e12.
14. Echodu DC, Yeka A, Eganyu T, Odude W, Bukenya F, Amoah B, et al. Impact of population based indoor residual spraying with and without mass drug administration with dihydroartemisinin-piperaquine on malaria prevalence in a high transmission setting: a quasi-experimental controlled before-and-after trial in northeastern Uganda. BMC Infectious Diseases. 2023;23(1):72.
15. Corbel V, Akogbeto M, Damien GB, Djenontin A, Chandre F, Rogier C, et al. Combination of malaria vector control interventions in pyrethroid resistance area in Benin: a cluster randomised controlled trial. The Lancet infectious diseases. 2012;12(8):617-26.
16. Nyarango PM, Gebremeskel T, Mebrahtu G, Mufunda J, Abdulmumini U, Ogbamariam A, et al. A steep decline of malaria morbidity and mortality trends in Eritrea between 2000 and 2004: the effect of combination of control methods. Malaria journal. 2006;5:1-13.
17. Davis KM, Worku A, Balkew M, Mumba P, Chibsa S, Tongren JE, et al. An observational study evaluating the epidemiological and entomological impacts of piperonyl butoxide insecticide-treated nets (ITNs) compared to a combination of indoor residual spraying (IRS) plus standard pyrethroid-only ITNs in Amhara Region, Ethiopia, 2019–2023. BMJ Global Health. 2025;10(1).
18. Nyarango PM, Gebremeskel T, Mebrahtu G, Mufunda J, Abdulmumini U, Ogbamariam A, et al. A steep decline of malaria morbidity and mortality trends in Eritrea

between 2000 and 2004: the effect of combination of control methods. Malaria journal. 2006;5(1):33.
19. Damien BG, Kesteman T, Dossou-Yovo GA, Dahounto A, Henry M-C, Rogier C, et al. Long-Lasting insecticide-treated nets combined or not with indoor residual spraying may not be sufficient to eliminate malaria: a case-control study, Benin, West Africa. Tropical Medicine and Infectious Disease. 2023;8(10):475.
20. Hien AS, Soma DD, Maiga S, Coulibaly D, Diabaté A, Belemvire A, et al. Evidence supporting deployment of next generation insecticide treated nets in Burkina Faso: bioassays with either chlorfenapyr or piperonyl butoxide increase mortality of pyrethroid-resistant Anopheles gambiae. Malaria journal. 2021;20(1):406.
21. Zhou Y, Zhang W-X, Tembo E, Xie M-Z, Zhang S-S, Wang X-R, et al. Effectiveness of indoor residual spraying on malaria control: a systematic review and meta-analysis. Infectious Diseases of Poverty. 2022;11(04):29-42.
22. Abong'o B, Gimnig JE, Torr SJ, Longman B, Omoke D, Muchoki M, et al. Impact of indoor residual spraying with pirimiphos-methyl (Actellic 300CS) on entomological indicators of transmission and malaria case burden in Migori County, western Kenya. Scientific reports. 2020;10(1):4518.
23. Pryce J, Medley N, Choi L. Indoor residual spraying for preventing malaria in communities using insecticide-treated nets. Cochrane Database of Systematic Reviews. 2022(1).

DGHS approval

Click here to access/download
Supporting Information
DGHS to UNCST.pdf

Click here to access/download
Supporting Information
Ethical approval_combinepdf.pdf

UNCST approval

Click here to access/download
Supporting Information
UNCST_rstug_UNCSTRefNumber.pdf

raw data

Click here to access/download
Supporting Information
Data 67_aligned.xlsx
