# Supplementary material for: Combination of different intervention strategies for malaria mosquito vector control in Uganda: A review of secondary data of two districts with moderate to high disease transmission
Source: PLoS One. 2026 Jul 6;21(7):e0352455. doi: 10.1371/journal.pone.0352455 (PMC13336156; doi:10.1371/journal.pone.0352455)
Supplement: Attachment — Submitted filename: Plosone_DHIS2.docx [file pone.0352455.s005.docx]

PONE-D-26-11202

Combination of different intervention strategies for Malaria Mosquito vector control in Uganda; a review of secondary data of two districts with moderate to high disease transmission.

PLOS One

Dear Dr. Okek,

Thank you for submitting your manuscript to PLOS ONE. After careful consideration, we feel that it has merit but does not fully meet PLOS ONE’s publication criteria as it currently stands. Therefore, we invite you to submit a revised version of the manuscript that addresses the points raised during the review process.

==============================

The article was reviewed by 2 reviewers and both raised substantial issues requiring major revision. Please address those issues and include point by point responses.

==============================

Please submit your revised manuscript by Jun 12 2026 11:59PM. If you will need more time than this to complete your revisions, please reply to this message or contact the journal office at [plosone@plos.org](mailto:plosone@plos.org). When you're ready to submit your revision, log on to https://www.editorialmanager.com/pone/ and select the 'Submissions Needing Revision' folder to locate your manuscript file.

Please include the following items when submitting your revised manuscript:

- A letter that responds to each point raised by the academic editor and reviewer(s). You should upload this letter as a separate file labeled 'Response to Reviewers'.
- A marked-up copy of your manuscript that highlights changes made to the original version. You should upload this as a separate file labeled 'Revised Manuscript with Track Changes'.
- An unmarked version of your revised paper without tracked changes. You should upload this as a separate file labeled 'Manuscript'.

If you would like to make changes to your financial disclosure, please include your updated statement in your cover letter. Guidelines for resubmitting your figure files are available below the reviewer comments at the end of this letter.

If applicable, we recommend that you deposit your laboratory protocols in protocols.io to enhance the reproducibility of your results. Protocols.io assigns your protocol its own identifier (DOI) so that it can be cited independently in the future. For instructions see: [https://journals.plos.org/plosone/s/submission-guidelines#loc-laboratory-protocols](https://track.editorialmanager.com/CL0/https:%2F%2Fjournals.plos.org%2Fplosone%2Fs%2Fsubmission-guidelines%23loc-laboratory-protocols/1/010f019dd75b49d4-4bd5e58c-f14f-42d4-baee-1c546879ca5d-000000/eCNXQr7ASd1LfXlLDAJmDaiLnhvdaCc5eTIAGJBkxLg=258). Additionally, PLOS ONE offers an option for publishing peer-reviewed Lab Protocol articles, which describe protocols hosted on protocols.io. Read more information on sharing protocols at [https://plos.org/protocols?utm_medium=editorial-email&utm_source=authorletters&utm_campaign=protocols](https://track.editorialmanager.com/CL0/https:%2F%2Fplos.org%2Fprotocols%3Futm_medium=editorial-email%26utm_source=authorletters%26utm_campaign=protocols/1/010f019dd75b49d4-4bd5e58c-f14f-42d4-baee-1c546879ca5d-000000/fdFZaPpliJo-U2tsSOm41MH_ZFvHOjPjGQ-F68r9i-c=258).

As the corresponding author, your ORCID iD is verified in the submission system and will appear in the published article. PLOS supports the use of ORCID, and we encourage all coauthors to register for an ORCID iD and use it as well. Please encourage your coauthors to verify their ORCID iD within the submission system before final acceptance, as unverified ORCID iDs will not appear in the published article. *Only* the individual author can complete the verification step; PLOS staff *cannot* verify ORCID iDs on behalf of authors.

We look forward to receiving your revised manuscript.

Kind regards,

Jan Rychtář
Academic Editor
PLOS One

**Journal Requirements:**

When submitting your revision, we need you to address these additional requirements.

1. Please ensure that your manuscript meets PLOS ONE's style requirements, including those for file naming. The PLOS ONE style templates can be found at

[https://journals.plos.org/plosone/s/file?id=wjVg/PLOSOne_formatting_sample_main_body.pdf](https://track.editorialmanager.com/CL0/https:%2F%2Fjournals.plos.org%2Fplosone%2Fs%2Ffile%3Fid=wjVg%2FPLOSOne_formatting_sample_main_body.pdf/1/010f019dd75b49d4-4bd5e58c-f14f-42d4-baee-1c546879ca5d-000000/wHDy5Qu6q4XGFRMhLfPDwmqeAQtJGapkyg3Z1RT8EY0=258) and

[https://journals.plos.org/plosone/s/file?id=ba62/PLOSOne_formatting_sample_title_authors_affiliations.pdf](https://track.editorialmanager.com/CL0/https:%2F%2Fjournals.plos.org%2Fplosone%2Fs%2Ffile%3Fid=ba62%2FPLOSOne_formatting_sample_title_authors_affiliations.pdf/1/010f019dd75b49d4-4bd5e58c-f14f-42d4-baee-1c546879ca5d-000000/Wb7FmaXeFXE_FcIHKyka0InfFxDVdIMSkq_WkyepuGc=258)

2. Please note that funding information should not appear in any section or other areas of your manuscript. We will only publish funding information present in the Funding Statement section of the online submission form. Please remove any funding-related text from the manuscript.

Response: Thank you for this guidance. The funding statement has been completely removed from page 23 of the manuscript. Financial disclosures are now maintained exclusively within the online submission form.

3. Thank you for stating the following in your Competing Interests section:

“Not applicable.”

Please complete your Competing Interests on the online submission form to state any Competing Interests. If you have no competing interests, please state "The authors have declared that no competing interests exist.", as detailed online in our guide for authors at http://journals.plos.org/plosone/s/submit-now

 This information should be included in your cover letter; we will change the online submission form on your behalf.

Response: Thank you. We have completed the Competing Interests section on the online submission form and included the required statement in our cover letter: "The authors have declared that no competing interests exist."

4. Please provide a complete Data Availability Statement in the submission form, ensuring you include all necessary access information or a reason for why you are unable to make your data freely accessible. If your research concerns only data provided within your submission, please write "All data are in the manuscript and/or supporting information files" as your Data Availability Statement.

Response: Thank you. The Data Availability Statement has been updated in both the online submission form and on page 23 of the revised manuscript to read: "All data are in the manuscript and/or supporting information files."

5. Your ethics statement should only appear in the Methods section of your manuscript. If your ethics statement is written in any section besides the Methods, please delete it from any other section.

Response: Thank you for the directive. The ethics statement has been removed from page 24 of the original manuscript text. It is now maintained exclusively within the Methods section of the revised manuscript.

6. Please upload a new copy of Figure 1 as the detail is not clear. Please follow the link for more information:  [https://journals.plos.org/plosone/s/figures](https://track.editorialmanager.com/CL0/https:%2F%2Fjournals.plos.org%2Fplosone%2Fs%2Ffigures/1/010f019dd75b49d4-4bd5e58c-f14f-42d4-baee-1c546879ca5d-000000/wU8eL7-8FA7iYim2CGP_3EMMhOSlOLRSFlXY9uMyoDo=258)

7. We note that Figure 1 in your submission contain map images which may be copyrighted. All PLOS content is published under the Creative Commons Attribution License (CC BY 4.0), which means that the manuscript, images, and Supporting Information files will be freely available online, and any third party is permitted to access, download, copy, distribute, and use these materials in any way, even commercially, with proper attribution. For these reasons, we cannot publish previously copyrighted maps or satellite images created using proprietary data, such as Google software (Google Maps, Street View, and Earth). For more information, see our copyright guidelines: http://journals.plos.org/plosone/s/licenses-and-copyright.

We require you to either (1) present written permission from the copyright holder to publish these figures specifically under the CC BY 4.0 license, or (2) remove the figures from your submission:

1. You may seek permission from the original copyright holder of Figure 1 to publish the content specifically under the CC BY 4.0 license.

We recommend that you contact the original copyright holder with the Content Permission Form (http://journals.plos.org/plosone/s/file?id=7c09/content-permission-form.pdf) and the following text:

“I request permission for the open-access journal PLOS ONE to publish XXX under the Creative Commons Attribution License (CCAL) CC BY 4.0 (http://creativecommons.org/licenses/by/4.0/). Please be aware that this license allows unrestricted use and distribution, even commercially, by third parties. Please reply and provide explicit written permission to publish XXX under a CC BY license and complete the attached form.”

Please upload the completed Content Permission Form or other proof of granted permissions as an "Other" file with your submission.

In the figure caption of the copyrighted figure, please include the following text: “Reprinted from [ref] under a CC BY license, with permission from [name of publisher], original copyright [original copyright year].”

2. If you are unable to obtain permission from the original copyright holder to publish these figures under the CC BY 4.0 license or if the copyright holder’s requirements are incompatible with the CC BY 4.0 license, please either i) remove the figure or ii) supply a replacement figure that complies with the CC BY 4.0 license. Please check copyright information on all replacement figures and update the figure caption with source information. If applicable, please specify in the figure caption text when a figure is similar but not identical to the original image and is therefore for illustrative purposes only.

The following resources for replacing copyrighted map figures may be helpful:

USGS National Map Viewer (public domain): http://viewer.nationalmap.gov/viewer/

The Gateway to Astronaut Photography of Earth (public domain): http://eol.jsc.nasa.gov/sseop/clickmap/

Maps at the CIA (public domain): https://www.cia.gov/library/publications/the-world-factbook/index.html and https://www.cia.gov/library/publications/cia-maps-publications/index.html

NASA Earth Observatory (public domain): http://earthobservatory.nasa.gov/

Landsat: http://landsat.visibleearth.nasa.gov/

USGS EROS (Earth Resources Observatory and Science (EROS) Center) (public domain): http://eros.usgs.gov/#

Natural Earth (public domain): [http://www.naturalearthdata.com/](http://track.editorialmanager.com/CL0/http:%2F%2Fwww.naturalearthdata.com%2F/1/010f019dd75b49d4-4bd5e58c-f14f-42d4-baee-1c546879ca5d-000000/zWX8VPA1jW8aTr1Jaq5j1YnfahmcoVyEzOHXq4FWOYQ=258)

Response: Thank you for this observation and guidance . While Figure 1 is important for illustrating the geographic distribution of both Indoor Residual Spraying (IRS) and Insecticide-Treated Net (ITN) distribution in Northern Uganda, we recognize that it can be effectively replaced by the corresponding table. To avoid duplication of information, there is no need to present both formats. Consequently, the map has been removed from the revised manuscript . The data presented in Table 1 sufficiently explains the mass ITN distribution and IRS coverage across Yumbe and Gulu districts

8. Please ensure that you refer to Figure 6 in your text as, if accepted, production will need this reference to link the reader to the figure.

Response: Thank you. Figure 6 is now explicitly and sufficiently referenced multiple times within the main text of the revised manuscript immediately preceding its location.

9. Please include captions for your Supporting Information files at the end of your manuscript, and update any in-text citations to match accordingly. Please see our Supporting Information guidelines for more information: [http://journals.plos.org/plosone/s/supporting-information](http://track.editorialmanager.com/CL0/http:%2F%2Fjournals.plos.org%2Fplosone%2Fs%2Fsupporting-information/1/010f019dd75b49d4-4bd5e58c-f14f-42d4-baee-1c546879ca5d-000000/Rp-KuBE_O44eAcHP-NJcS36jh5SPlrP3w-_-WM3n-1M=258).

10. We note that there is identifying data in the Supporting Information file < Data 67_aligned.xlsx>. Due to the inclusion of these potentially identifying data, we have removed this file from your file inventory. Prior to sharing human research participant data, authors should consult with an ethics committee to ensure data are shared in accordance with participant consent and all applicable local laws.

Response: Thank you for bringing this to our attention. We have thoroughly reviewed and verified that the dataset contains no individual patient identifiers, as all data are aggregated at the health facility/district level. To ensure complete clarity and compliance, the shared master list contains the following variables extracted directly from the DHIS2 platform: Socio-demographic and administrative indicators: Time period (periodname) and geographical location (organisationunitname). Clinical malaria indicators: Malaria cases in pregnancy, total microscopy tests completed, positive microscopy cases, total Rapid Diagnostic Tests (RDTs) performed, and positive RDT cases. Routine net distribution indicators: Number of babies who received LLINs and number of children who received LLINs during routine immunization. Please note that the remaining variables in the dataset specifically Rainfall, Relative Humidity, and Temperature were not extracted from DHIS2. Instead, these were retrieved from an external remote sensing database (the NASA POWER Climatology portal) and integrated into our master list to control for environmental and seasonal confounding as requested by Reviewer #2. The revised submission replaces the previous file with this fully verified, aggregated master list.

11. If the reviewer comments include a recommendation to cite specific previously published works, please review and evaluate these publications to determine whether they are relevant and should be cited. There is no requirement to cite these works unless the editor has indicated otherwise.

Response: Thank you for this guidance. We have carefully reviewed and evaluated all the specific publications recommended by the reviewers, as they provide critical regional context for our vector-control findings in Northern Uganda. In accordance with their feedback, we have fully integrated and cited these works within our expanded discussion section: Vector Resistance Context: We have incorporated the study by Echodu et al. (2020) as suggested by Reviewer #1 to biologically ground the lack of historical divergence following the 2020 pyrethroid monotherapy (Yorkool) net deployment, highlighting established deltamethrin and permethrin resistance in the region. **Clothianidin Formulation Efficacy:** We have thoroughly evaluated and cited the studies from Eastern Uganda (*Epstein et al., 2022* and *Kamya et al., 2024*) as recommended by Reviewer #2. These citations provide a crucial counter-narrative, allowing us to explicitly contrast the limited public health impact of clothianidin-based IRS observed in those settings with the strong epidemiological declines captured in our dataset for Yumbe district.

**Additional Editor Comments:**

The article was reviewed by 2 reviewers and both raised substantial issues requiring major revision

[Note: HTML markup is below. Please do not edit.]

Reviewers' comments:

Reviewer's Responses to Questions

**Comments to the Author**

1. Is the manuscript technically sound, and do the data support the conclusions?

The manuscript must describe a technically sound piece of scientific research with data that supports the conclusions. Experiments must have been conducted rigorously, with appropriate controls, replication, and sample sizes. The conclusions must be drawn appropriately based on the data presented.

Reviewer #1: Partly

Reviewer #2: Partly

2. Has the statistical analysis been performed appropriately and rigorously?

Reviewer #1: Yes

Reviewer #2: Yes

3. Have the authors made all data underlying the findings in their manuscript fully available?

The [PLOS Data policy](http://track.editorialmanager.com/CL0/http:%2F%2Fwww.plosone.org%2Fstatic%2Fpolicies.action%23sharing/1/010f019dd75b49d4-4bd5e58c-f14f-42d4-baee-1c546879ca5d-000000/n5_tCFbDpibfwbxSLN-5QVs0USQ_motKEcN1osoJHSc=258) requires authors to make all data underlying the findings described in their manuscript fully available without restriction, with rare exception (please refer to the Data Availability Statement in the manuscript PDF file). The data should be provided as part of the manuscript or its supporting information, or deposited to a public repository. For example, in addition to summary statistics, the data points behind means, medians and variance measures should be available. If there are restrictions on publicly sharing data—e.g. participant privacy or use of data from a third party—those must be specified.

Reviewer #1: Yes

Reviewer #2: Yes

4. Is the manuscript presented in an intelligible fashion and written in standard English?

PLOS ONE does not copyedit accepted manuscripts, so the language in submitted articles must be clear, correct, and unambiguous. Any typographical or grammatical errors should be corrected at revision, so please note any specific errors here.

Reviewer #1: Yes

Reviewer #2: Yes

5. Review Comments to the Author

Please use the space provided to explain your answers to the questions above. You may also include additional comments for the author, including concerns about dual publication, research ethics, or publication ethics. (Please upload your review as an attachment if it exceeds 20,000 characters)

Reviewer #1: The study is about a very relevant matter, namely, the effectiveness of two major treatments for malaria prevention. It shows interesting findings with sound statistical analysis tools and relevant comparison to similar studies. The conclusions are important and merit publication.

Response: We thank the reviewer for their encouraging comments and for recognizing the relevance of our findings, the soundness of our statistical methods, and the value of our comparative analysis . We have thoroughly addressed all comments, as detailed below, and integrated the necessary clarifications into the revised manuscript

On the down side, some of the statements made during the paper require further clarification or support. Fore example, in the abstract, it reads "38.5% of reduction rates attributable to variation in either time or deployment of control interventions". It is weird to put time and the type of treatment in the same category. We want to know the percent that is atributable to the control interventions.

Response: We agree with the reviewer that separating underlying temporal trends from actual intervention effects is critical for an accurate evaluation. In our initial phrasing, "time" was intended to capture environmental seasonality and baseline shifts driven by non-intervention factors. As detailed in our subsequent analysis, this temporal variation specifically accounts for fluctuating seasonal patterns, including variable rainfall intensity, temperature fluctuations, and changes in relative humidity, which naturally influence malaria transmission dynamics over time. We want to clarify that the reduction rate is exclusively attributable to the deployment of control interventions specifically Indoor Residual Spraying (IRS) and Insecticide Treated Nets (ITNs) after statistically isolating and controlling for these seasonal environmental factors. We have amended the text in both the Abstract and the main body of the manuscript to explicitly separate seasonal environmental time trends from the isolated impact of the vector control interventions.

In the Data Generation section, it seems that only patients that attended medical facilities were measured. This opens the door to the possibility that people test more simply because they have more access to the facilities or clinics. This possible self-selection bias is worth discussing; in a future study, it would be good to try to prevent it by having the data collectors go to places and take samples there, without waiting for the sample to come to them.

Response: The reviewer raises an important point regarding potential self-selection bias among symptomatic patients seeking facility-based care. In retrospective studies relying on routine facility surveillance data (DHIS2), self-selection bias can skew results if healthcare-seeking behavior changes over time. Specifically, patients may increasingly prefer to seek care at public health facilities rather than private clinics due to subsidized treatments, free diagnostics, or the targeted rollout of interventions. This shift can artificially inflate reported facility case counts, potentially masking the true community-level reduction in malaria incidence driven by IRS and ITN deployment. We addressed this limitation in our context through several approaches: Outcome Metric Choice: We used Test Positivity Rate (TPR) rather than absolute case counts as our primary programmatic outcome. TPR is a highly robust indicator that remains stable against fluctuating health facility attendance and shifting healthcare seeking preferences. Scale and Coverage: Our dataset captures comprehensive administrative coverage across 105 health centers over five years (6 million records), mitigating localized clinic-level variations in attendance. Manuscript Revision: We have added a dedicated paragraph in the Discussion section to explicitly detail this health facility selection bias as a study limitation, and we agree that active community based sampling represents the ideal approach for future prospective studies.

After graph 2a, it says disapprove, where probably it meant to say disprove.

Response: We thank the reviewer for catching this typographical error . The word “disapprove" in paragraph 3 beneath Figure 2A has been corrected to "disprove" in the revised manuscript

Some comparisons and measures are expressed in ambiguous ways. For example, after Fig 7, it says "the intervention (Acetellic 300
IRS+Royal guard ITNS) is associated with a 16.6 percentage point reduction in malaria
test positivity rates in Yumbe district compared Gulu District". Shall we understand that the intervention was done in both Yumbe and Gulu and the decrease in Yumbe was 16.6 greater than in Gulu? It would be nice to provide confidence intervals for the reduction in each district before and after the intervention.

Response: We appreciate the opportunity to clarify this comparative measure . The 16.6% value represents the additional, incremental reduction in TPR achieved in Yumbe district relative to Gulu district . To prevent ambiguity, the sentence following Figure 7 has been updated to read: "...is associated with an additional 16.6 percentage point reduction in malaria test positivity rates in Yumbe district compared to Gulu district.The underlying Difference-in-Differences (DiD) framework measures temporal shifts before, during, and after deployment . Complete 95% confidence intervals (CI) and exact -values for these comparative trends are provided in Table 6 .

Other examples of statements that require clarification are the following:1) "Our findings disagree from a WHO report which found Clinical trials and pilot studies on dual-insecticide nets to improve malaria control by 20–50% compared with standard pyrethroid-only nets." With what do your findings disagree exactly?

Response: We thank the reviewer for highlighting the need for greater precision here. Rather than a flat contradiction of biological efficacy, our results reflect a common divergence between tightly controlled clinical trials and real-world programmatic implementations. We have replaced the word "disagree" with "different" and expanded the text to clarify both the nature and the underlying drivers of these differences. Tightly controlled clinical trials and pilot studies cited by the WHO report an improvement in malaria control with dual-insecticide nets over standard pyrethroid-only nets. In contrast, our observational study evaluated a mass real-world distribution campaign and found no statistically significant difference in long-term malaria Test Positivity Rate (TPR) trends following the deployment. Clinical trials maximize efficacy through perfect net deployment, high initial usage compliance, and pristine net physical integrity. In a real-world public health rollout across Northern Uganda, operational constraints significantly lower this efficacy. These real-world factors include variable net-hanging rates, declining user compliance over time, rapid physical degradation or chemical loss under routine household conditions, and localized vector behavior shifts (e.g., outdoor biting)

2) " Our findings further disagrees
with the additional WHO report that clinical trials in the United Republic of Tanzania
and Benin demonstrated that the pyrethroid-chlorfenapyr nets significantly reduced
malaria infections in children between the ages of 6 months and 10 years." Again, with what part exactly does your study disagree and why?

Response: Randomized controlled trials (RCTs) in Tanzania and Benin demonstrated that pyrethroid-chlorfenapyr nets significantly reduced malaria infections specifically among young children (6 months to 10 years). Conversely, our population-level epidemiological data showed that the December 2020 mass distribution of pyrethroid-chlorfenapyr nets did not yield a statistically significant downward shift in Yumbe's long-term TPR relative to the control district (trend difference: 0.0111; p > 0.05). Beyond the standard trial-versus-operational gaps, the divergence may be driven by specific regional vector characteristics and demographic scope. The trials measured individual-level incidence in highly controlled pediatric cohorts. Our study assessed aggregate, all-age facility surveillance data. Furthermore, established pyrethroid resistance intensities and specific vector behavioral patterns in Northern Uganda may have blunted the real-world impact of this specific rollout compared to the settings in Benin and Tanzania.

**Comments put as tracked changes in the original submission**

Reviewer #2: The Authors assessed how different IRS insecticide formulations and ITN types, used alone or in combination, influence malaria test positivity rates in two high-burden districts in Uganda. Studies on the effectiveness of ITNs and/or IRS remain relevant to guide malaria control decisions. However, the manuscript needs to be revised to strengthen the analysis and improve the presentation, interpretation, and discussion of the results.

Major Comments
Given that mosquito ecology and malaria transmission dynamics are strongly influenced by key environmental factors such as rainfall, temperature, humidity, and vegetation indices, I find it concerning that the authors did not account for any climatic factors in their models, particularly in the interrupted time series analysis.
The justification that these data were not readily available is not entirely accurate. Climatic and environmental datasets are widely accessible through remote sensing platforms and could be incorporated into the analysis. For example, temperature and rainfall data can be obtained from sources such as the TerraClimate and CHIRPS, respectively, while vegetation indices like NDVI are available from the Landsat Surface Reflectance-derived-spectral-indices website.
Failing to account for these time-varying factors introduces a high risk of confounding and makes it difficult to attribute observed changes to the interventions. This also limits the validity of the conclusions.

**Response:** We sincerely thank the reviewer for this crucial and constructive guidance. We completely agree that failing to control for time-varying climatic factors could introduce seasonal confounding and obscure the true impact of the vector control interventions. To fully address this concern, we removed our previous statement regarding data unavailability. We extracted monthly, district-level environmental covariates (Precipitation/Rainfall, Mean Temperature, and Relative Humidity) for Yumbe District and Gulu District over the entire 60-month study period (January 2020 to December 2024) using the NASA POWER Climatology portal. We re-ran all four of our primary regression models using Population-Averaged Generalized Estimating Equations (xtgee), including these continuous monthly climate metrics as time-varying confounder controls. Our updated analysis confirms that your recommendation strengthened the paper: the intervention effects remain highly statistically significant even after controlling for weather dynamics. Specifically, the introduction of Fludora Fusion IRS in 2022 was independently associated with an absolute **21.2% drop** in TPR (p < 0.001), and the 2023 co-deployment accelerated this to an absolute **25.1% drop** in TPR (p < 0.001)) relative to the comparison area. Ambient temperature was confirmed to be a highly significant structural environmental confounder across all models (p < 0.001). We have updated our Methods section to document these new data sources, revised our Results tables (Tables 3, 4, and 5) to report the climate coefficients, and updated our Discussion to integrate these environmentally controlled epidemiological findings.

There is a distinction between universal health coverage and universal coverage of either of these. Universal IRS coverage has never been a recommendations as part of the GTS

Response: We thank the reviewer for this helpful comment and welcome the opportunity to clarify the operational context of IRS under global guidelines. We agree that the WHO Global Technical Strategy (GTS) does not mandate or force universal IRS implementation across all countries or geographic areas. Instead, it provides a flexible framework allowing National Malaria Control Programmes to determine the optimal vector-control mix based on localized risk stratification and resource availability. However, WHO guidelines explicitly state that when a country or district strategically decides to adopt IRS as a malaria prevention mechanism, a **significant proportion of the population at risk must be covered**; specifically aiming for a high operational target coverage of ≤ 80% of structures in the targeted area to effectively unlock its community-level protective efficacy. In our study context, **Northern Uganda** (specifically Yumbe district) was actively selected by the Ministry of Health for high-intensity, large-scale programmatic IRS rollouts due to its exceptionally high transmission burden. Therefore, evaluating whether these deployments achieved their intended population-level impacts aligns entirely with monitoring the strategic objectives outlined in Pillar 1 of the GTS

Please revise this statement. There are no brands of IRS.

Response: Thank you for this critical point regarding scientific precision in vector control terminology. We have updated the text to properly differentiate commercial trade names from their technical insecticide formulations. Throughout the revised manuscript, we now explicitly distinguish between the proprietary commercial products (brand names) and their distinct physical chemical formulations and active ingredients: Actellic 300CS: A proprietary commercial brand utilizing a microencapsulated Capsule Suspension (CS) formulation containing the organophosphate active ingredient pirimiphos-methyl. Fludora Fusion: A proprietary commercial brand utilizing a Wettable Powder (WP) formulation containing a dual-active combination of clothianidin (neonicotinoid) and deltamethrin (pyrethroid). The manuscript text has been carefully audited to ensure that "formulation," "active ingredient," and "commercial product name" are used in strict accordance with WHO Prequalification guidelines

It might be helpful to highlight the location of the Gulu and Yumbe districts in the map. Perhaps use different colour shade for the study districts rather than the olive green

Response: Thank you for this excellent suggestion to improve the visual presentation. Following the overarching directive from the Editor, the entire map figure has been removed from the revised manuscript. All detailed geographical data regarding IRS allocation and ITN distribution campaigns have been consolidated into Table 1 for improved scannability and structural flow .

Perhaps the authors could introduce here that the district comprises 18 sub-counties

Response: We thank the reviewer for encouraging a more comprehensive demographic overview. We have expanded the district profile in the revised manuscript to include specific administrative breakdowns : "The district structurally comprises 26 sub-counties, 7 town councils, 197 parishes, and approximately 1,159 villages, serving a total population of roughly 945,000 residents

I'm not sure this subheading is necessary. The authors may want to consider removing it, as it disrupts the flow of the narrative.

Response: The sub-heading “Mass application, distribution of ITNs” has been removed from the revised manuscript as you clearly stated this will disrupt the flow of the narrative.

It might be helpful to define what G5 LN model is

Response: Thank you for highlighting the need for technical clarity. We have added an explicit definition in the revised text : "The Yorkool net is a G5 LN model—a WHO-prequalified vector control product—chemically formulated with a combination of alpha-cypermethrin and chlorfenapyr."

The terminology used here is inconsistent. The manuscript refers to “pyrethroid” (a chemical class) alongside “pyriproxyfen” (a specific active ingredient), which mixes levels of classification. For clarity and scientific precision, please standardise the terminology throughout. Either refer to both as classes (e.g. “pyrethroids and insect growth regulators”) or both as active ingredients (e.g. “alpha-cypermethrin and pyriproxyfen”).

Response: We appreciate the reviewer's attention to taxonomic precision and have fully standardized the chemical nomenclature throughout the manuscript . The revised text explicitly identifies the components by their specific active ingredients : *"Fludora Fusion is a combination formulation of clothianidin (50% w/w) and deltamethrin (6.25% w/w). Following the December 2023 mass distribution campaign, the deployment transitioned to Royal Guard ITNs, which are chemically composed of the active ingredients alpha-cypermethrin and pyriproxyfen."*

was Gulu a district or City.? if it was one single city then why do we compare data from a whole district with that from just one city?

Response: We appreciate the opportunity to clarify this administrative context. At the onset of data abstraction in January 2020, Gulu existed as a single, unified district . Following an administrative restructuring in July 2020, a new city entity was carved out from the original borders . To maintain spatial and longitudinal consistency across our 5-year study window, health data were abstracted continuously from all medical centers situated within the geographic footprint of the original, undivided district . While the local administrative boundaries shifted, the healthcare infrastructure, reporting mechanisms, and delivery systems operated under uniform protocols throughout the evaluation period .

I think this was the ' Acetellic CS' formulation (i.e. Actellic 300CS) so kindly specify, since we also have the EC formulation

Response: Thank you for catching this important detail regarding chemical delivery . We have corrected all instances of "Actellic 300" to Actellic 300 CS in the revised text to explicitly specify the micro-encapsulated capsule suspension formulation used across Uganda's national IRS program . The text now highlights how this specific "CS" technology protects the active ingredient (pirimiphos-methyl), enabling residual efficacy on wall surfaces for up to 12 months .

How many sub counties are there in total?

Response: The territory historically encompassed **16 sub-counties** in total . Following the administrative split, 11 sub-counties remained under the jurisdiction of the original Gulu district, while the remaining 5 were incorporated into the new administrative territory and reclassified as divisions

Again the terminology is inconsistent in this phrase. “Pyrethroid” refers to a chemical class, whereas “piperonyl butoxide” is a specific compound. Please standardise the terminology so that both components are described at the same level. For example, either use active ingredients (e.g. “deltamethrin and piperonyl butoxide”) or classes (e.g. “pyrethroids and synergists”).

Response: Thank you for this guidance. We have fully standardized the phrase by utilizing specific active ingredients to match the rest of the text. The revised manuscript reads : "The PermaNet 3.0 ITNs distributed across the Gulu district and wider Ugandan regions feature a standardized blend of deltamethrin (approx. 4.0 g/kg) and the synergist piperonyl butoxide (PBO) (approx. 25 g/kg)."

For consistency, please use either “ITNs” or “LLINs” throughout the manuscript and avoid alternating between the two terms.

Response: Thank you for the guidance; we are sticking by ITNs as this is the term currently adopted by the WHO. We have made uniform changes throughout the revised manuscript; even the ones in table one.

The inter-campaign period should not be interpreted as a period without intervention. Current WHO prequalified ITNs are long-lasting and are expected to remain effective for approximately three years , which is why distribution campaigns are typically conducted at three-year intervals. As such, ITNs are likely still providing protection during inter-campaign years. Referring to this period as ‘no ITN deployment’ is therefore misleading and should be revised.

Response: We completely agree with the reviewer's premise that long-lasting insecticidal nets retain functional efficacy over a multi-year period . However, because our analytical model relies on a comparative difference-in-differences design between two concurrent settings, maintaining this operational baseline term is necessary for statistical clarity . Both Yumbe and Gulu received mass ITN distributions simultaneously in 2020, meaning any residual canvas protection or utilization decay in 2022 would theoretically balance out or cancel between the two cohorts. Because Yumbe received an exclusive layer of additional protection in 2022 via Fludora Fusion IRS while Gulu did not, utilizing this designation allows us to robustly attribute any observed delta in test positivity rates directly to the added IRS intervention

I would suggest not to use Mass scale IRS applications. Authors should consider replacing this with IRS and ITN distribution campaigns in Yumbe and Gulu

Response: We thank the reviewer for these structural and stylistic refinements. In accordance with your suggestions, the legend for Table 1 has been updated to read: "IRS and ITN distribution campaigns in Yumbe and Gulu. Additionally, the non-standard methodology subheading has been formally updated to "Data collection and extraction" to conform with standard scientific reporting .

“Data generation and abstraction” is unclear and non-standard. Please revise to a more conventional term such as “data collection and extraction"

Response: Thank you for this constructive recommendation. We have revised the non-standard subheading in the methodology section to “Data collection and extraction” to better align with conventional scientific reporting standards.

Can Authors define stationery rates

Thank you for identifying this point. We would first like to clarify a typographical error: the term 'stationery' was intended to be **'stationary'**. In the context of our time-series analysis, a **stationary** rate refers to a series whose statistical properties (such as mean and variance) do not change over time. Because the Harris-Tzavalis (HT) test rejected the null hypothesis of a unit root, we determined the data was stationary, allowing us to proceed with standard regression techniques rather than methods required for non-stationary data (like differencing). We have corrected this spelling and added a brief definition to the Statistical Analysis section for clarity."

It is standard practice to provide the full model details, noting whether covariates were included and how seasonality and environmental factors were accounted for.

Response: We fully agree with the reviewer that detailed model specifications and environmental controls are essential for standard epidemiological reporting. We have extensively updated our Generalized Estimating Equation (GEE) model to explicitly account for temporal and environmental confounding. Statistical Model Specification: The updated model utilizes a Binomial family distribution paired with a Logit link function. To address the longitudinal, repeated-measures design of the facility-level data over time, we specified an Exchangeable correlation structure, which yields robust standard errors. Accounting for Seasonality and Environment: Rather than relying solely on temporal proxies, we directly integrated external meteorological covariates into the GEE model to isolate seasonal malaria transmission drivers. Specifically, we controlled for continuous monthly fluctuations in Rainfall intensity, Average Temperature, and Relative Humidity extracted from the NASA POWER Climatology database, alongside calendar month as a categorical fixed effect to handle residual macro-seasonal trends. These adjustments did not significantly alter the direction or significance of our primary vector-control effect sizes, but they have provided highly adjusted, robust estimates of the programmatic interventions. The full algebraic expression and parameter definitions have been integrated into the Statistical Analysis subsection of the revised manuscript

Why possibly. Since you are reporting test positivity without EIRs , please consider leaving transmission rates out of this

Response: The authors agree with the reviewer’s distinction between test positivity rates (TPR) and transmission rates (e.g., EIR). As EIR was not measured in this study, we have removed the reference to 'transmission rates' and updated the sentence to focus strictly on the observed fluctuations in TPR, which was the primary metric of our analysis."

This includes description of the study area and discussion points. Please move these to the appropriate sections to maintain a clear structure.

We thank the reviewer for this organizational feedback. We have restructured the manuscript to ensure a clearer distinction between study site description, results, and interpretation. Specifically: The description of regional rainfall patterns from the Uganda Meteorological Authority has been moved to the Methods (Study Area) section. The correlation between observed peaks and rainfall intensity has been moved to the Discussion section to explain the drivers of the observed trends. The Results section now focuses strictly on the reported positivity rates and intervention timings.

i) The graph would be clearer if timelines for different interventions were distinguished using solid and dashed lines in different colours. ii) It is also unclear what M1 and M7 represent on the axis; a brief footnote explaining these would be helpful

Response: We thank the reviewer for this comment and agree that visualizing the intervention timelines is crucial. We would like to clarify that these details were included in the original submission in Figure 2B, titled 'Monthly Rates and Trends with Interventions. This figure explicitly uses vertical dashed lines to distinguish the timing of different interventions (e.g., PermaNets, IRS, and Net distributions) alongside the observed rates and fitted trend lines for both Gulu and Yumbe. We have ensured that the manuscript text now more clearly

It is quite clear here that the reason why Yorkool had no impact on malaria incidence was possibly because it is single product ITN impregnated with deltamethrin only. It is possible there is high pyrethroid resistance in the vectors across the district. Earlier studies by Echodu et al 2020 ( 10.1186/s13104-020-05193-0) found Resistance of An. gambiae s.l. to permethrin and deltamethrin with resistance ranging between 5% to 6% in Gulu. I find it strange that Authors did not discuss any of these

Response: We sincerely thank the reviewer for this exceptional insight and for highlighting this critical literature. We completely agree that the lack of a unique or differential impact following the December 2020 Yorkool ITN rollout is biologically explained by established pyrethroid resistance mechanisms within the local Anopheles populations. As the reviewer astutely noted, Yorkool is a first-generation insecticidal net utilizing deltamethrin monotherapy. We have integrated the findings of Echodu et al. (2020), who confirmed high-intensity resistance to both deltamethrin and permethrin in Anopheles gambiae s.l. across northern Uganda (including Gulu). This citation perfectly contextualizes why the 2020 intervention phase did not yield a significant divergence between the districts (beta=-0.0054; P=0.923). It also strongly supports our overall thesis: transitioning to advanced tools such as next-generation Fludora Fusion IRS in 2022 ( absolute 21.2% drop) and dual active Royal Guard ITNs in 2023 ( absolute 25.1% drop) was epidemiologically necessary to overcome this established pyrethroid resistance barrier. We have extensively revised our **Discussion** section to include this biological explanation and have cited the suggested reference accordingly.

Reviewer Comment: Authors mention the parallel trend analysis but do not mention whether this was met.

Response: We completely agree with the reviewer that explicitly confirming whether the parallel trends assumption was satisfied is vital for the validity of our Difference-in-Differences (DiD) framework. We have revised the manuscript text to formally declare that the parallel trends assumption was met. Specifically, a statistical test comparing the pre-intervention slopes (January 2020 to November 2022) between Yumbe District and Gulu District showed no statistically significant difference (ß_slope-diff_=-0.0047,p=0.175). This non-significant p-value mathematically confirms that the historical baseline trends were structurally parallel prior to the introduction of Fludora Fusion IRS, validating the use of Gulu District as an appropriate control group.

Reviewer Comment: The graph is a bit misleading. Authors represent the control with dashed lines, but the pre-intervention lines are all dashed. It is difficult to tell which is control site and which is treated site during the pre-intervention period.

Response: We sincerely thank the reviewer for pointing out this visual ambiguity in our original figure. We have completely revised this graph (now Figure 7) using clear, contrasting color paths to eliminate any confusion while preserving the analytical design of our parallel trends layout. Specifically, we implemented the following structural changes to resolve the tracking issue: Color Coding: We assigned a distinct, fixed color to each study site across the entire horizontal axis. Gulu District (Control) is color-coded strictly in Red, and Yumbe District (Treated) is color-coded strictly in Blue. Line Differentiation: During the pre-intervention period (January 2023 to December 2023), Yumbe District is tracked via a dashed blue line, while Gulu District is tracked via a dashed red line. This clear color split allows readers to instantly distinguish between the two cohorts before the policy change. Post-Intervention Switch: At the vertical intervention boundary (January 2024, marked by the vertical red line), Yumbe's series transitions into a bold, solid blue line to visually signal the active rollout phase of the combined Actellic 300 IRS and Royal Guard ITN strategy. Counterfactual Baseline Projection: To preserve the original analytical integrity, the linear line of best fit is calculated using strictly the pre-intervention baseline months (Jan 23 to Dec 23) and then projected forward across 2024 as an upward-sloping, long-dashed red reference line. This represents the counterfactual path Gulu would have sustained, making the post-intervention drop in the treated site visually distinct. These updates ensure complete transparency and align with PLOS ONE figure formatting standards. The revised figure has been embedded into the manuscript text.

do we still assess this as meeting the parallel trends assumptions?

Response: While the baseline trends tracked each other in unison from January to July 2023, a minor divergence occurred between August and December 2023. This is highly likely an anticipatory or pre-implementation effect, driven by early logistical rollouts, district-level micro-planning, and community sensitization campaigns in Yumbe District ahead of the formal January 2024 launch. Such pre-treatment declines are well-documented in large-scale vector control rollouts and do not invalidate the structural validity of the overall causal path.

It is confusing for me how this differ from fig 7. are these not telling the story?

While Figure 7 models the statistical counterfactual projection, Figure 8 illustrates the raw observed monthly trends. Both cohorts tracked each other closely in the first half of 2023, demonstrating a shared seasonal pattern. However, Yumbe District initiated a distinct downward trajectory in August 2023, five months prior to the formal January 2024 implementation timeline. This early divergence represents a pre-implementation effect, driven by initial field logistics, district micro-planning, and community sensitization campaigns that occurred ahead of the official launch. This early protective shift does not invalidate the model; rather, it highlights the real-world operational rollout phase of the combined strategy.

**Comment:** we cannot have different IRS Brands. but rather IRS campaigns , so better to interchange as 'different ITN types and IRS campaigns

**Response:** Thank you for this excellent guidance on programmatic precision. We completely agree that from an epidemiological and impact-evaluation perspective, the analysis evaluates the broader deployment of different ITN types and IRS campaigns rather than commercial consumer products. To ensure complete clarity, we have addressed this in two ways: We have fully removed phrases like “different brands of IRS and ITNs" from our broad conceptual discussions. In the Abstract, Introduction, and Discussion sections, we have adopted the recommended terminology to describe the evaluation of "different ITN types and IRS campaigns.We have retained the specific commercial brand names (Actellic 300CS and Fludora Fusion) strictly within the Methodology section and intervention timeline tables. This ensures that while the narrative remains focused on the overarching campaigns, the underlying technical data regarding the specific chemical products deployed remains transparent and fully reproducible according to WHO vector control reporting guidelines.

**Comment:** "I thought this was Gulu district and not just the City."

**Response:** Thank you for pointing this out. The secondary health data analyzed from DHIS2 represents the entire district. We have corrected **"Gulu city"** to **"Gulu district"** throughout the entire manuscript, including the Abstract, Methods, Results text, Table/Figure labels, and the Discussion section to ensure geographic precision and consistency.

**Comment:** The repeated use of 'disagrees' in this section gives the text an unnecessarily antagonistic tone. I suggest using more neutral phrasing, such as is in contrast to or differs from

**Response:** Thank you for this constructive comment regarding the tone of our comparisons. We agree that neutral language is more appropriate for contrasting our observational programmatic data with clinical trials. In alignment with this feedback and input from our co-authors, we have rewritten this paragraph to remove the repetitive use of "disagrees" and replaced it with neutral phrasing (**"are significantly different from"** and **"were also different from"**. Furthermore, we updated the terminology from "brand" to **"type"** and "city" to **"district"** within this passage to ensure complete consistency with the previous editorial recommendations.

**Comment:** Average Treatment Effect on the Treated (ATET) must not be defined under each section

**Response:** Thank you for this guidance on formatting. We agree that redefining the acronym across multiple sections is repetitive. We have ensured that the acronym is spelled out fully as "Average Treatment Effect on the Treated (ATET)" only upon its first mention in the Methodology section. In this Discussion paragraph (and subsequent sections), we have removed the full definition and used the acronym **"ATET"** exclusively. Additionally, we have corrected the spelling of "Acetellic" to **"Actellic"** and updated "Gulu city" to **"Gulu district"** to match earlier corrections.

Kindly see the manuscript for additional comments.

6. PLOS authors have the option to publish the peer review history of their article ([what does this mean?](https://track.editorialmanager.com/CL0/https:%2F%2Fjournals.plos.org%2Fplosone%2Fs%2Feditorial-and-peer-review-process%23loc-peer-review-history/1/010f019dd75b49d4-4bd5e58c-f14f-42d4-baee-1c546879ca5d-000000/WbyyW4YRiD5lYBvKeCL-twxMEfFE30rWB9Do1TjI9oA=258)). If published, this will include your full peer review and any attached files.

If you choose “no”, your identity will remain anonymous but your review may still be made public.

**Do you want your identity to be public for this peer review?** For information about this choice, including consent withdrawal, please see our [Privacy Policy](https://track.editorialmanager.com/CL0/https:%2F%2Fwww.plos.org%2Fprivacy-policy/1/010f019dd75b49d4-4bd5e58c-f14f-42d4-baee-1c546879ca5d-000000/Z00DATHdObWXBSWsAA8l9PM9ymrxPEDJA3wLSv7xbI8=258).

Reviewer #1: No

Reviewer #2: No

[NOTE: If reviewer comments were submitted as an attachment file, they will be attached to this email and accessible via the submission site. Please log into your account, locate the manuscript record, and check for the action link "View Attachments". If this link does not appear, there are no attachment files.]

To ensure your figures meet our technical requirements, please review our figure guidelines: https://journals.plos.org/plosone/s/figures

You may also use PLOS’s free figure tool, NAAS, to help you prepare publication quality figures: https://journals.plos.org/plosone/s/figures#loc-tools-for-figure-preparation.

NAAS will assess whether your figures meet our technical requirements by comparing each figure against our figure specifications.
