# Supplementary material for: Combination of different intervention strategies for malaria mosquito vector control in Uganda: A review of secondary data of two districts with moderate to high disease transmission
Source: PLoS One. 2026 Jul 6;21(7):e0352455. doi: 10.1371/journal.pone.0352455 (PMC13336156; doi:10.1371/journal.pone.0352455)
Supplement: Peer review history

# Peer review: Decision Letter 0

**DOI:** 10.1371/journal.pone.0352455.r001

**Authors:** Jan Rychtář

28 Apr 2026

PONE-D-26-11202

Combination of different intervention strategies for Malaria Mosquito vector control in Uganda; a review of secondary data of two districts with moderate to high disease transmission.

PLOS One

Dear Dr. Okek,

Thank you for submitting your manuscript to PLOS ONE. After careful consideration, we feel that it has merit but does not fully meet PLOS ONE’s publication criteria as it currently stands. Therefore, we invite you to submit a revised version of the manuscript that addresses the points raised during the review process.

==============================

The article was reviewed by 2 reviewers and both raised substantial issues requiring major revision. Please address those issues and include point by point responses.

==============================

Please submit your revised manuscript by Jun 12 2026 11:59PM. If you will need more time than this to complete your revisions, please reply to this message or contact the journal office at plosone@plos.org. When you're ready to submit your revision, log on to https://www.editorialmanager.com/pone/ and select the 'Submissions Needing Revision' folder to locate your manuscript file.

Please include the following items when submitting your revised manuscript:

- A letter that responds to each point raised by the academic editor and reviewer(s). You should upload this letter as a separate file labeled 'Response to Reviewers'.
- A marked-up copy of your manuscript that highlights changes made to the original version. You should upload this as a separate file labeled 'Revised Manuscript with Track Changes'.
- An unmarked version of your revised paper without tracked changes. You should upload this as a separate file labeled 'Manuscript'.

If you would like to make changes to your financial disclosure, please include your updated statement in your cover letter. Guidelines for resubmitting your figure files are available below the reviewer comments at the end of this letter.

If applicable, we recommend that you deposit your laboratory protocols in protocols.io to enhance the reproducibility of your results. Protocols.io assigns your protocol its own identifier (DOI) so that it can be cited independently in the future. For instructions see: https://journals.plos.org/plosone/s/submission-guidelines#loc-laboratory-protocols. Additionally, PLOS ONE offers an option for publishing peer-reviewed Lab Protocol articles, which describe protocols hosted on protocols.io. Read more information on sharing protocols at https://plos.org/protocols?utm_medium=editorial-email&utm_source=authorletters&utm_campaign=protocols.

As the corresponding author, your ORCID iD is verified in the submission system and will appear in the published article. PLOS supports the use of ORCID, and we encourage all coauthors to register for an ORCID iD and use it as well. Please encourage your coauthors to verify their ORCID iD within the submission system before final acceptance, as unverified ORCID iDs will not appear in the published article. *Only*  the individual author can complete the verification step; PLOS staff *cannot*  verify ORCID iDs on behalf of authors.

We look forward to receiving your revised manuscript.

Kind regards,

Jan Rychtář

Academic Editor

PLOS One

**Journal Requirements:**

When submitting your revision, we need you to address these additional requirements.

1. Please ensure that your manuscript meets PLOS ONE's style requirements, including those for file naming. The PLOS ONE style templates can be found at

https://journals.plos.org/plosone/s/file?id=wjVg/PLOSOne_formatting_sample_main_body.pdf and

https://journals.plos.org/plosone/s/file?id=ba62/PLOSOne_formatting_sample_title_authors_affiliations.pdf

2. Please note that funding information should not appear in any section or other areas of your manuscript. We will only publish funding information present in the Funding Statement section of the online submission form. Please remove any funding-related text from the manuscript.

3. Thank you for stating the following in your Competing Interests section:

“Not applicable.”

Please complete your Competing Interests on the online submission form to state any Competing Interests. If you have no competing interests, please state "The authors have declared that no competing interests exist.", as detailed online in our guide for authors at http://journals.plos.org/plosone/s/submit-now

This information should be included in your cover letter; we will change the online submission form on your behalf.

4. Please provide a complete Data Availability Statement in the submission form, ensuring you include all necessary access information or a reason for why you are unable to make your data freely accessible. If your research concerns only data provided within your submission, please write "All data are in the manuscript and/or supporting information files" as your Data Availability Statement.

5. Your ethics statement should only appear in the Methods section of your manuscript. If your ethics statement is written in any section besides the Methods, please delete it from any other section.

6. Please upload a new copy of Figure 1 as the detail is not clear. Please follow the link for more information:  https://journals.plos.org/plosone/s/figures

7. We note that Figure 1 in your submission contain map images which may be copyrighted. All PLOS content is published under the Creative Commons Attribution License (CC BY 4.0), which means that the manuscript, images, and Supporting Information files will be freely available online, and any third party is permitted to access, download, copy, distribute, and use these materials in any way, even commercially, with proper attribution. For these reasons, we cannot publish previously copyrighted maps or satellite images created using proprietary data, such as Google software (Google Maps, Street View, and Earth). For more information, see our copyright guidelines: http://journals.plos.org/plosone/s/licenses-and-copyright.

We require you to either (1) present written permission from the copyright holder to publish these figures specifically under the CC BY 4.0 license, or (2) remove the figures from your submission:

1. You may seek permission from the original copyright holder of Figure 1 to publish the content specifically under the CC BY 4.0 license.

We recommend that you contact the original copyright holder with the Content Permission Form (http://journals.plos.org/plosone/s/file?id=7c09/content-permission-form.pdf) and the following text:

“I request permission for the open-access journal PLOS ONE to publish XXX under the Creative Commons Attribution License (CCAL) CC BY 4.0 (http://creativecommons.org/licenses/by/4.0/). Please be aware that this license allows unrestricted use and distribution, even commercially, by third parties. Please reply and provide explicit written permission to publish XXX under a CC BY license and complete the attached form.”

Please upload the completed Content Permission Form or other proof of granted permissions as an "Other" file with your submission.

In the figure caption of the copyrighted figure, please include the following text: “Reprinted from [ref] under a CC BY license, with permission from [name of publisher], original copyright [original copyright year].”

2. If you are unable to obtain permission from the original copyright holder to publish these figures under the CC BY 4.0 license or if the copyright holder’s requirements are incompatible with the CC BY 4.0 license, please either i) remove the figure or ii) supply a replacement figure that complies with the CC BY 4.0 license. Please check copyright information on all replacement figures and update the figure caption with source information. If applicable, please specify in the figure caption text when a figure is similar but not identical to the original image and is therefore for illustrative purposes only.

The following resources for replacing copyrighted map figures may be helpful:

USGS National Map Viewer (public domain): http://viewer.nationalmap.gov/viewer/

The Gateway to Astronaut Photography of Earth (public domain): http://eol.jsc.nasa.gov/sseop/clickmap/

Maps at the CIA (public domain): https://www.cia.gov/library/publications/the-world-factbook/index.html and https://www.cia.gov/library/publications/cia-maps-publications/index.html

NASA Earth Observatory (public domain): http://earthobservatory.nasa.gov/

Landsat: http://landsat.visibleearth.nasa.gov/

USGS EROS (Earth Resources Observatory and Science (EROS) Center) (public domain): http://eros.usgs.gov/#

Natural Earth (public domain): http://www.naturalearthdata.com/

8. Please ensure that you refer to Figure 6 in your text as, if accepted, production will need this reference to link the reader to the figure.

9. Please include captions for your Supporting Information files at the end of your manuscript, and update any in-text citations to match accordingly. Please see our Supporting Information guidelines for more information: http://journals.plos.org/plosone/s/supporting-information.

10. We note that there is identifying data in the Supporting Information file < Data 67_aligned.xlsx>. Due to the inclusion of these potentially identifying data, we have removed this file from your file inventory. Prior to sharing human research participant data, authors should consult with an ethics committee to ensure data are shared in accordance with participant consent and all applicable local laws.

11. If the reviewer comments include a recommendation to cite specific previously published works, please review and evaluate these publications to determine whether they are relevant and should be cited. There is no requirement to cite these works unless the editor has indicated otherwise.

**Additional Editor Comments:**

The article was reviewed by 2 reviewers and both raised substantial issues requiring major revision

[Note: HTML markup is below. Please do not edit.]

Reviewers' comments:

Reviewer's Responses to Questions

**Comments to the Author**

1. Is the manuscript technically sound, and do the data support the conclusions?

The manuscript must describe a technically sound piece of scientific research with data that supports the conclusions. Experiments must have been conducted rigorously, with appropriate controls, replication, and sample sizes. The conclusions must be drawn appropriately based on the data presented.

Reviewer #1: Partly

Reviewer #2: Partly

**********

2. Has the statistical analysis been performed appropriately and rigorously?

Reviewer #1: Yes

Reviewer #2: Yes

**********

3. Have the authors made all data underlying the findings in their manuscript fully available?

The PLOS Data policy requires authors to make all data underlying the findings described in their manuscript fully available without restriction, with rare exception (please refer to the Data Availability Statement in the manuscript PDF file). The data should be provided as part of the manuscript or its supporting information, or deposited to a public repository. For example, in addition to summary statistics, the data points behind means, medians and variance measures should be available. If there are restrictions on publicly sharing data—e.g. participant privacy or use of data from a third party—those must be specified.

Reviewer #1: Yes

Reviewer #2: Yes

**********

4. Is the manuscript presented in an intelligible fashion and written in standard English?

PLOS ONE does not copyedit accepted manuscripts, so the language in submitted articles must be clear, correct, and unambiguous. Any typographical or grammatical errors should be corrected at revision, so please note any specific errors here.

Reviewer #1: Yes

Reviewer #2: Yes

**********

5. Review Comments to the Author

Please use the space provided to explain your answers to the questions above. You may also include additional comments for the author, including concerns about dual publication, research ethics, or publication ethics. (Please upload your review as an attachment if it exceeds 20,000 characters)

Reviewer #1: The study is about a very relevant matter, namely, the effectiveness of two major treatments for malaria prevention. It shows interesting findings with sound statistical analysis tools and relevant comparison to similar studies. The conclusions are imporant and merit publication. On the down side, some of the statements made during the paper require further clarification or support. Fore example, in the abstract, it reads "38.5% of reduction rates attributable to variation in either time or deployment of control interventions". It is weird to put time and the type of treatment in the same category. We want to know the percent that is atributable to the control interventions. In the Data Generation section, it seems that only patients that attended medical facilities were measured. This opens the door to the possibility that people test more simply because they have more access to the facilities or clinics. This possible self-selection bias is worth discussing; in a future study, it would be good to try to prevent it by having the data collectors go to places and take samples there, without waiting for the sample to come to them. After graph 2a, it says disapprove, where probably it meant to say disprove. Some comparisons and measures are expressed in ambiguous ways. For example, after Fig 7, it says "the intervention (Acetellic 300

IRS+Royal guard ITNS) is associated with a 16.6 percentage point reduction in malaria

test positivity rates in Yumbe district compared Gulu City". Shall we understand that the intervention was done in both Yumbe and Gulu and the decrease in Yumbe was 16.6 greater than in Gulu? It would be nice to provide confidence intervals for the reduction in each city before and after the intervention.

Other examples of statements that require clarification are the following:

1) "Our findings disagree from a WHO report which

found Clinical trials and pilot studies on dual-insecticide nets to improve malaria control

by 20–50% compared with standard pyrethroid-only nets." With what do your findings disagree exactly? With then umber 20-50%?

2) " Our findings further disagrees

with the additional WHO report that clinical trials in the United Republic of Tanzania

and Benin demonstrated that the pyrethroid-chlorfenapyr nets significantly reduced

malaria infections in children between the ages of 6 months and 10 years." Again, with what part exactly does your study disagree and why?

Reviewer #2: The Authors assessed how different IRS insecticide formulations and ITN types, used alone or in combination, influence malaria test positivity rates in two high-burden districts in Uganda. Studies on the effectiveness of ITNs and/or IRS remain relevant to guide malaria control decisions. However, the manuscript needs to be revised to strengthen the analysis and improve the presentation, interpretation, and discussion of the results.

Major Comments

Given that mosquito ecology and malaria transmission dynamics are strongly influenced by key environmental factors such as rainfall, temperature, humidity, and vegetation indices, I find it concerning that the authors did not account for any climatic factors in their models, particularly in the interrupted time series analysis.

The justification that these data were not readily available is not entirely accurate. Climatic and environmental datasets are widely accessible through remote sensing platforms and could be incorporated into the analysis. For example, temperature and rainfall data can be obtained from sources such as the TerraClimate and CHIRPS, respectively, while vegetation indices like NDVI are available from the Landsat Surface Reflectance-derived-spectral-indices website.

Failing to account for these time-varying factors introduces a high risk of confounding and makes it difficult to attribute observed changes to the interventions. This also limits the validity of the conclusions.

Here is a summary of some additional issues:

#1.The authors consider inter-ITN campaign periods as “no intervention.” This is incorrect because WHO-prequalified ITNs typically remain effective for about 3 years, except in specific cases such as PBO ITNs (with reported durability that averages about 18-24 months in some studies), and their protective effect is expected to last throughout the nets' lifetime. Therefore, observations during these periods should not be classified as no intervention, contrary to the current approach. It is also not clear how the authors incorporated interventions or accounted for intervention coverage in their models.

#2. The parallel trends assumption appears uncertain (particularly in Fig 8). A formal pre-trend test (e.g. treatment × time interaction restricted to the pre-intervention period) should be conducted and reported.

#3. The discussions need revision to strengthen the paper. In this version, the authors mainly compare their findings with other studies but offer limited interpretation and minimal discussion of the implications. From the current analysis and results presented it is clear that the effectiveness of interventions is heavily influenced by the insecticide class and formulation or ITN type. I feel this is an important point that should be emphasised.

#4. One of the main findings is that IRS with Fludora fusion contributed to declines in malaria cases in this study area. This reported impact of Fludora Fusion contrasts with findings from other settings in Uganda (https://doi.org/10.1371/journal.pgph.0003254; https://doi.org/10.1371/journal.pgph.0000676 and https://doi.org/10.21203/rs.3.rs-7518583/v1) where clothianidin-based IRS has shown limited impact. I think this is a significant find, assuming the results do not change after the recommended revisions/analysis and should be discussed in depth. The authors should (i) situate findings within the broader literature, ii) explore possible explanations (e.g. prior IRS exposure, ecological differences) iii) report and interpret the magnitude of effect more explicitly.

#5. It is unclear whether Gulu refers to an entire district or a single city. The authors have used city and district interchangeably. This creates some confusion and raises concerns about comparability. We cannot compare data from a district and that of a township/city.

Minor comments:

#6. The Results section includes elements of study description and discussion. These should be moved to the appropriate sections to maintain a clear and logical structure.

#7. A few observations on terminology, consistency, and tone

- Terminology should be standardised throughout the manuscript. The authors should consistently use either active ingredients, product formulations, or class-level terms, rather than mixing these. For example, alternating between Actellic 300CS and pirimiphos-methyl, or SumiShield/Fludora and clothianidin/deltamethrin, creates confusion. Similarly, there is inconsistency in the level of classification used. In some sections, chemical classes (e.g. “pyrethroid”) are presented alongside specific active ingredients (e.g. “pyriproxyfen”). This should be standardised to either class-level or active-ingredient-level terminology for clarity and consistency

- Authors should consider using either “ITNs” (recommended) or “LLINs” consistently throughout. And should also avoid repeated definitions of IRS and ITNs once introduced, and ensure abbreviations are used consistently after definition.

- Replace words phrasing like “mass scale IRS applications” with standard terminology, either as IRS campaigns or mass ITN distribution

#8. Figures

- Figure 1: Authors should consider using distinct colour shading especially for Gulu and Yumbe.

- Figure 2A:

1. Use clearer distinctions between intervention periods (e.g. colour and line type).

2. Define “M1” and “M7” in a footnote and also add footnotes indicating timing of ITN distributions.

- Figure 8 looks similar to Figure 7. It could be helpful for authors to clarify how they differ or to consider consolidating them if they are redundant.

#9. Tables

- Table 4: revise headings to “Lower 95% CI” and “Upper 95% CI”.

- It would be helpful to Standardise decimal places across all tables and text.

Kindly see the manuscript for additional comments.

**********

6. PLOS authors have the option to publish the peer review history of their article (what does this mean?). If published, this will include your full peer review and any attached files.

If you choose “no”, your identity will remain anonymous but your review may still be made public.

**Do you want your identity to be public for this peer review?**  For information about this choice, including consent withdrawal, please see our Privacy Policy.

Reviewer #1: No

Reviewer #2: No

**********

[NOTE: If reviewer comments were submitted as an attachment file, they will be attached to this email and accessible via the submission site. Please log into your account, locate the manuscript record, and check for the action link "View Attachments". If this link does not appear, there are no attachment files.]

To ensure your figures meet our technical requirements, please review our figure guidelines: https://journals.plos.org/plosone/s/figures

You may also use PLOS’s free figure tool, NAAS, to help you prepare publication quality figures: https://journals.plos.org/plosone/s/figures#loc-tools-for-figure-preparation.

NAAS will assess whether your figures meet our technical requirements by comparing each figure against our figure specifications.

Attachment Submitted filename: Reviewer Comments on Manuscript Number PONE-D-26-11202.pdf

Attachment Submitted filename: PONE-D-26-11202_Review Feedback.pdf

# Author comment: Author response to Decision Letter 1

**DOI:** 10.1371/journal.pone.0352455.r002

22 May 2026

PONE-D-26-11202

Combination of different intervention strategies for Malaria Mosquito vector control in Uganda; a review of secondary data of two districts with moderate to high disease transmission.

PLOS One

Dear Dr. Okek,

Thank you for submitting your manuscript to PLOS ONE. After careful consideration, we feel that it has merit but does not fully meet PLOS ONE’s publication criteria as it currently stands. Therefore, we invite you to submit a revised version of the manuscript that addresses the points raised during the review process.

==============================

The article was reviewed by 2 reviewers and both raised substantial issues requiring major revision. Please address those issues and include point by point responses.

==============================

Please submit your revised manuscript by Jun 12 2026 11:59PM. If you will need more time than this to complete your revisions, please reply to this message or contact the journal office at plosone@plos.org. When you're ready to submit your revision, log on to https://www.editorialmanager.com/pone/ and select the 'Submissions Needing Revision' folder to locate your manuscript file.

Please include the following items when submitting your revised manuscript:

• A letter that responds to each point raised by the academic editor and reviewer(s). You should upload this letter as a separate file labeled 'Response to Reviewers'.

• A marked-up copy of your manuscript that highlights changes made to the original version. You should upload this as a separate file labeled 'Revised Manuscript with Track Changes'.

• An unmarked version of your revised paper without tracked changes. You should upload this as a separate file labeled 'Manuscript'.

If you would like to make changes to your financial disclosure, please include your updated statement in your cover letter. Guidelines for resubmitting your figure files are available below the reviewer comments at the end of this letter.

If applicable, we recommend that you deposit your laboratory protocols in protocols.io to enhance the reproducibility of your results. Protocols.io assigns your protocol its own identifier (DOI) so that it can be cited independently in the future. For instructions see: https://journals.plos.org/plosone/s/submission-guidelines#loc-laboratory-protocols. Additionally, PLOS ONE offers an option for publishing peer-reviewed Lab Protocol articles, which describe protocols hosted on protocols.io. Read more information on sharing protocols at https://plos.org/protocols?utm_medium=editorial-email&utm_source=authorletters&utm_campaign=protocols.

As the corresponding author, your ORCID iD is verified in the submission system and will appear in the published article. PLOS supports the use of ORCID, and we encourage all coauthors to register for an ORCID iD and use it as well. Please encourage your coauthors to verify their ORCID iD within the submission system before final acceptance, as unverified ORCID iDs will not appear in the published article. Only the individual author can complete the verification step; PLOS staff cannot verify ORCID iDs on behalf of authors.

We look forward to receiving your revised manuscript.

Kind regards,

Jan Rychtář

Academic Editor

PLOS One

Journal Requirements:

When submitting your revision, we need you to address these additional requirements.

1. Please ensure that your manuscript meets PLOS ONE's style requirements, including those for file naming. The PLOS ONE style templates can be found at

https://journals.plos.org/plosone/s/file?id=wjVg/PLOSOne_formatting_sample_main_body.pdf and

https://journals.plos.org/plosone/s/file?id=ba62/PLOSOne_formatting_sample_title_authors_affiliations.pdf

2. Please note that funding information should not appear in any section or other areas of your manuscript. We will only publish funding information present in the Funding Statement section of the online submission form. Please remove any funding-related text from the manuscript.

Response: Thank you for this guidance. The funding statement has been completely removed from page 23 of the manuscript. Financial disclosures are now maintained exclusively within the online submission form.

3. Thank you for stating the following in your Competing Interests section:

“Not applicable.”

Please complete your Competing Interests on the online submission form to state any Competing Interests. If you have no competing interests, please state "The authors have declared that no competing interests exist.", as detailed online in our guide for authors at http://journals.plos.org/plosone/s/submit-now

This information should be included in your cover letter; we will change the online submission form on your behalf.

Response: Thank you. We have completed the Competing Interests section on the online submission form and included the required statement in our cover letter: "The authors have declared that no competing interests exist."

4. Please provide a complete Data Availability Statement in the submission form, ensuring you include all necessary access information or a reason for why you are unable to make your data freely accessible. If your research concerns only data provided within your submission, please write "All data are in the manuscript and/or supporting information files" as your Data Availability Statement.

Response: Thank you. The Data Availability Statement has been updated in both the online submission form and on page 23 of the revised manuscript to read: "All data are in the manuscript and/or supporting information files."

5. Your ethics statement should only appear in the Methods section of your manuscript. If your ethics statement is written in any section besides the Methods, please delete it from any other section.

Response: Thank you for the directive. The ethics statement has been removed from page 24 of the original manuscript text. It is now maintained exclusively within the Methods section of the revised manuscript.

6. Please upload a new copy of Figure 1 as the detail is not clear. Please follow the link for more information: https://journals.plos.org/plosone/s/figures

7. We note that Figure 1 in your submission contain map images which may be copyrighted. All PLOS content is published under the Creative Commons Attribution License (CC BY 4.0), which means that the manuscript, images, and Supporting Information files will be freely available online, and any third party is permitted to access, download, copy, distribute, and use these materials in any way, even commercially, with proper attribution. For these reasons, we cannot publish previously copyrighted maps or satellite images created using proprietary data, such as Google software (Google Maps, Street View, and Earth). For more information, see our copyright guidelines: http://journals.plos.org/plosone/s/licenses-and-copyright.

We require you to either (1) present written permission from the copyright holder to publish these figures specifically under the CC BY 4.0 license, or (2) remove the figures from your submission:

1. You may seek permission from the original copyright holder of Figure 1 to publish the content specifically under the CC BY 4.0 license.

We recommend that you contact the original copyright holder with the Content Permission Form (http://journals.plos.org/plosone/s/file?id=7c09/content-permission-form.pdf) and the following text:

“I request permission for the open-access journal PLOS ONE to publish XXX under the Creative Commons Attribution License (CCAL) CC BY 4.0 (http://creativecommons.org/licenses/by/4.0/). Please be aware that this license allows unrestricted use and distribution, even commercially, by third parties. Please reply and provide explicit written permission to publish XXX under a CC BY license and complete the attached form.”

Please upload the completed Content Permission Form or other proof of granted permissions as an "Other" file with your submission.

In the figure caption of the copyrighted figure, please include the following text: “Reprinted from [ref] under a CC BY license, with permission from [name of publisher], original copyright [original copyright year].”

2. If you are unable to obtain permission from the original copyright holder to publish these figures under the CC BY 4.0 license or if the copyright holder’s requirements are incompatible with the CC BY 4.0 license, please either i) remove the figure or ii) supply a replacement figure that complies with the CC BY 4.0 license. Please check copyright information on all replacement figures and update the figure caption with source information. If applicable, please specify in the figure caption text when a figure is similar but not identical to the original image and is therefore for illustrative purposes only.

The following resources for replacing copyrighted map figures may be helpful:

USGS National Map Viewer (public domain): http://viewer.nationalmap.gov/viewer/

The Gateway to Astronaut Photography of Earth (public domain): http://eol.jsc.nasa.gov/sseop/clickmap/

Maps at the CIA (public domain): https://www.cia.gov/library/publications/the-world-factbook/index.html and https://www.cia.gov/library/publications/cia-maps-publications/index.html

NASA Earth Observatory (public domain): http://earthobservatory.nasa.gov/

Landsat: http://landsat.visibleearth.nasa.gov/

USGS EROS (Earth Resources Observatory and Science (EROS) Center) (public domain): http://eros.usgs.gov/#

Natural Earth (public domain): http://www.naturalearthdata.com/

Response: Thank you for this observation and guidance . While Figure 1 is important for illustrating the geographic distribution of both Indoor Residual Spraying (IRS) and Insecticide-Treated Net (ITN) distribution in Northern Uganda, we recognize that it can be effectively replaced by the corresponding table. To avoid duplication of information, there is no need to present both formats. Consequently, the map has been removed from the revised manuscript . The data presented in Table 1 sufficiently explains the mass ITN distribution and IRS coverage across Yumbe and Gulu districts

8. Please ensure that you refer to Figure 6 in your text as, if accepted, production will need this reference to link the reader to the figure.

Response: Thank you. Figure 6 is now explicitly and sufficiently referenced multiple times within the main text of the revised manuscript immediately preceding its location.

9. Please include captions for your Supporting Information files at the end of your manuscript, and update any in-text citations to match accordingly. Please see our Supporting Information guidelines for more information: http://journals.plos.org/plosone/s/supporting-information.

10. We note that there is identifying data in the Supporting Information file < Data 67_aligned.xlsx>. Due to the inclusion of these potentially identifying data, we have removed this file from your file inventory. Prior to sharing human research participant data, authors should consult with an ethics committee to ensure data are shared in accordance with participant consent and all applicable local laws.

Response: Thank you for bringing this to our attention. We have thoroughly reviewed and verified that the dataset contains no individual patient identifiers, as all data are aggregated at the health facility/district level. To ensure complete clarity and compliance, the shared master list contains the following variables extracted directly from the DHIS2 platform: Socio-demographic and administrative indicators: Time period (periodname) and geographical location (organisationunitname). Clinical malaria indicators: Malaria cases in pregnancy, total microscopy tests completed, positive microscopy cases, total Rapid Diagnostic Tests (RDTs) performed, and positive RDT cases. Routine net distribution indicators: Number of babies who received LLINs and number of children who received LLINs during routine immunization. Please note that the remaining variables in the dataset specifically Rainfall, Relative Humidity, and Temperature were not extracted from DHIS2. Instead, these were retrieved from an external remote sensing database (the NASA POWER Climatology portal) and integrated into our master list to control for environmental and seasonal confounding as requested by Reviewer #2. The revised submission replaces the previous file with this fully verified, aggregated master list.

11. If the reviewer comments include a recommendation to cite specific previously published works, please review and evaluate these publications to determine whether they are relevant and should be cited. There is no requirement to cite these works unless the editor has indicated otherwise.

Response: Thank you for this guidance. We have carefully reviewed and evaluated all the specific publications recommended by the reviewers, as they provide critical regional context for our vector-control findings in Northern Uganda. In accordance with their feedback, we have fully integrated and cited these works within our expanded discussion section: Vector Resistance Context: We have incorporated the study by Echodu et al. (2020) as suggested by Reviewer #1 to biologically ground the lack of historical divergence following the 2020 pyrethroid monotherapy (Yorkool) net deployment, highlighting established deltamethrin and permethrin resistance in the region. Clothianidin Formulation Efficacy: We have thoroughly evaluated and cited the studies from Eastern Uganda (Epstein et al., 2022 and Kamya et al., 2024) as recommended by Reviewer #2. These citations provide a crucial counter-narrative, allowing us to explicitly contrast the limited public health impact of clothianidin-based IRS observed in those settings with the strong epidemiological declines captured in our dataset for Yumbe district.

Additional Editor Comments:

The article was reviewed by 2 reviewers and both raised substantial issues requiring major revision

[Note: HTML markup is below. Please do not edit.]

Reviewers' comments:

Reviewer's Responses to Questions

Comments to the Author

1. Is the manuscript technically sound, and do the data support the conclusions?

The manuscript must describe a technically sound piece of scientific research with data that supports the conclusions. Experiments must have been conducted rigorously, with appropriate controls, replication, and sample sizes. The conclusions must be drawn appropriately based on the data presented.

Reviewer #1: Partly

Reviewer #2: Partly

________________________________________

2. Has the statistical analysis been performed appropriately and rigorously?

Reviewer #1: Yes

Reviewer #2: Yes

________________________________________

3. Have the authors made all data underlying the findings in their manuscript fully available?

The PLOS Data policy requires authors to make all data underlying the findings described in their manuscript fully available without restriction, with rare exception (please refer to the Data Availability Statement in the manuscript PDF file). The data should be provided as part of the manuscript or its supporting information, or deposited to a public repository. For example, in addition to summary statistics, the data points behind means, medians and variance measures should be available. If there are restrictions on publicly sharing data—e.g. participant privacy or use of data from a third party—those must be specified.

Reviewer #1: Yes

Reviewer #2: Yes

________________________________________

4. Is the manuscript presented in an intelligible fashion and written in standard English?

PLOS ONE does not copyedit accepted manuscripts, so the language in submitted articles must be clear, correct, and unambiguous. Any typographical or grammatical errors should be corrected at revision, so please note any specific errors here.

Reviewer #1: Yes

Reviewer #2: Yes

________________________________________

5. Review Comments to the Author

Please use the space provided to explain your answers to the questions above. You may also include additional comments for the author, including concerns about dual publication, research ethics, or publication ethics. (Please upload your review as an attachment if it exceeds 20,000 characters)

Reviewer #1: The study is about a very relevant matter,

Attachment Submitted filename: Plosone_DHIS2.docx

# Peer review: Decision Letter 1

**DOI:** 10.1371/journal.pone.0352455.r003

**Authors:** Jan Rychtář, Jan Rychtář

10 Jun 2026

Combination of different intervention strategies for Malaria Mosquito vector control in Uganda; a review of secondary data of two districts with moderate to high disease transmission.

PONE-D-26-11202R1

Dear Dr. Okek,

We’re pleased to inform you that your manuscript has been judged scientifically suitable for publication and will be formally accepted for publication once it meets all outstanding technical requirements.

Within one week, you’ll receive an e-mail detailing the required amendments. When these have been addressed, you’ll receive a formal acceptance letter and your manuscript will be scheduled for publication.

An invoice will be generated when your article is formally accepted. Please note, if your institution has a publishing partnership with PLOS and your article meets the relevant criteria, all or part of your publication costs will be covered. Please make sure your user information is up-to-date by logging into Editorial Manager at Editorial Manager® and clicking the ‘Update My Information' link at the top of the page. For questions related to billing, please contact billing support.

If your institution or institutions have a press office, please notify them about your upcoming paper to help maximize its impact. If they’ll be preparing press materials, please inform our press team as soon as possible -- no later than 48 hours after receiving the formal acceptance. Your manuscript will remain under strict press embargo until 2 pm Eastern Time on the date of publication. For more information, please contact onepress@plos.org.

Kind regards,

Jan Rychtář

Academic Editor

PLOS One

Additional Editor Comments (optional):

All comments addressed

Reviewers' comments:

Reviewer's Responses to Questions

**Comments to the Author**

1. If the authors have adequately addressed your comments raised in a previous round of review and you feel that this manuscript is now acceptable for publication, you may indicate that here to bypass the “Comments to the Author” section, enter your conflict of interest statement in the “Confidential to Editor” section, and submit your "Accept" recommendation.

Reviewer #2: All comments have been addressed

**********

2. Is the manuscript technically sound, and do the data support the conclusions?

The manuscript must describe a technically sound piece of scientific research with data that supports the conclusions. Experiments must have been conducted rigorously, with appropriate controls, replication, and sample sizes. The conclusions must be drawn appropriately based on the data presented.

Reviewer #2: Yes

**********

3. Has the statistical analysis been performed appropriately and rigorously?

Reviewer #2: Yes

**********

4. Have the authors made all data underlying the findings in their manuscript fully available?

The PLOS Data policy requires authors to make all data underlying the findings described in their manuscript fully available without restriction, with rare exception (please refer to the Data Availability Statement in the manuscript PDF file). The data should be provided as part of the manuscript or its supporting information, or deposited to a public repository. For example, in addition to summary statistics, the data points behind means, medians and variance measures should be available. If there are restrictions on publicly sharing data—e.g. participant privacy or use of data from a third party—those must be specified.

Reviewer #2: Yes

**********

5. Is the manuscript presented in an intelligible fashion and written in standard English?

PLOS ONE does not copyedit accepted manuscripts, so the language in submitted articles must be clear, correct, and unambiguous. Any typographical or grammatical errors should be corrected at revision, so please note any specific errors here.

Reviewer #2: Yes

**********

6. Review Comments to the Author

Please use the space provided to explain your answers to the questions above. You may also include additional comments for the author, including concerns about dual publication, research ethics, or publication ethics. (Please upload your review as an attachment if it exceeds 20,000 characters)

Reviewer #2: The authors have addressed the main queries raised. This version is a much-improved version. I would however advise that authors do a thorough check for minor typos and consistency of trade/brand names of ITNs (eg. "PermaNets ITN " vs "PermaNet ITN")

I have no additional comments

**********

7. PLOS authors have the option to publish the peer review history of their article (what does this mean?). If published, this will include your full peer review and any attached files.

If you choose “no”, your identity will remain anonymous but your review may still be made public.

**Do you want your identity to be public for this peer review?**  For information about this choice, including consent withdrawal, please see our Privacy Policy.

Reviewer #2: No

**********

# Editor report: Acceptance letter

**DOI:** 10.1371/journal.pone.0352455.r004

**Authors:** Jan Rychtář, Jan Rychtář

PONE-D-26-11202R1

PLOS One

Dear Dr. Okek,

I'm pleased to inform you that your manuscript has been deemed suitable for publication in PLOS One. Congratulations! Your manuscript is now being handed over to our production team.

At this stage, our production department will prepare your paper for publication. This includes ensuring the following:

* All references, tables, and figures are properly cited

* All relevant supporting information is included in the manuscript submission,

* There are no issues that prevent the paper from being properly typeset

You will receive further instructions from the production team, including instructions on how to review your proof when it is ready. Please keep in mind that we are working through a large volume of accepted articles, so please give us a few days to review your paper and let you know the next and final steps.

Lastly, if your institution or institutions have a press office, please let them know about your upcoming paper now to help maximize its impact. If they'll be preparing press materials, please inform our press team within the next 48 hours. Your manuscript will remain under strict press embargo until 2 pm Eastern Time on the date of publication. For more information, please contact onepress@plos.org.

You will receive an invoice from PLOS for your publication fee after your manuscript has reached the completed accept phase. If you receive an email requesting payment before acceptance or for any other service, this may be a phishing scheme. Learn how to identify phishing emails and protect your accounts at https://explore.plos.org/phishing.

If we can help with anything else, please email us at customercare@plos.org.

Thank you for submitting your work to PLOS ONE and supporting open access.

Kind regards,

PLOS ONE Editorial Office Staff

on behalf of

Dr. Jan Rychtář

Academic Editor

PLOS One
